# Giant spin-orbit magnetic state readout enhanced by a magnetic tunnel junction

**DOI:** 10.1038/s41467-026-73382-9

**Published:** 2026-05-26

**Authors:** Yan Huang, Kun Zhang, Guo Liu, Xiaobai Ning, Shiyang Lu, Shijie Xu, Qing Yang, Wenlong Cai, Renyou Xu, Yuxuan Yao, Yu He, Jinkai Wang, Bo Li, Haozhe Yang, Kewen Shi, Kaihua Cao, Chao Zhao, Yue Zhang, Weisheng Zhao

**Affiliations:** 1 State Key Laboratory of Spintronics, Hangzhou International Innovation Institute & School of Integrated Circuit Science and Engineering, Beihang University, Hangzhou, PR China; 2 Fert Beijing Research Institute, MIIT Key Laboratory of Spintronics, School of Integrated Circuit Science and Engineering, Beihang University, Beijing, PR China; 3 Integrated Circuit and Intelligent Instruments Innovation Center, Qingdao Research Institute, Beihang University, Qingdao, PR China; 4 State Key Laboratory of Spintronics Devices and Technologies, School of Integrated Circuits, Nanjing University, Suzhou, PR China

**Keywords:** Electronic devices, Magnetic devices, Information storage

## Abstract

Magnetoelectric spin-orbit (MESO) logic, composed of a voltage-controlled magnetoelectric writing module and a spin-orbit readout module, is highly expected to substitute the silicon-based transistors and enable energy-efficient and scalable computing. Nevertheless, the output voltage of readout module based on spin-to-charge conversion is far less than the minimum magnetoelectric writing voltage, which greatly restricts the cascading function of MESO logic. Here, we first propose a magnetic tunnel junction (MTJ)-enhanced MESO logic to implement giant readout signal. Up to 1.5 mV output voltage is obtained, marking a significant improvement of approximately two orders of magnitude compared to previous findings. We ascribe the substantial enhancement to current modulation by junction resistance and the spin filtering effect of MgO-based MTJ. Moreover, the naturally integrated MTJ and MESO enables instantaneous and nonvolatile data exchange between computing module and external unit. Our work not only enhances output signal of readout module for direct cascading of MESO logic but also refines the design architecture, marking a pivotal stride forward in propelling MESO technology toward practical applications.

## Abstract (web summary)

This work proposes an MTJ-enhanced magnetoelectric spin-orbit (MESO) logic architecture for giant spin-orbit magnetic state readout. The device achieves a room-temperature output voltage up to 1.5 mV and instant and efficient data exchange between computing and memory units.

## Introduction

With technology nodes shrinking, conventional complementary metal-oxide-semiconductor (CMOS) technology faces a growing hurdle in scaling down due to leakage current in the OFF state, imposing a limit on the overall system energy efficiency^1,2^. As a solution, emerging nonvolatile memories, featuring normally-OFF/instantly-ON computation, can overcome the static power brought by leakage current^3–7^. However, because these technologies are not supported to generate an effective driving force, they are hard to drive the subsequent data writing and construct cascaded logic circuits^2,7^.

Magnetoelectric spin-orbit (MESO) logic, utilizing voltage-controlled magnetoelectric (ME) effect for data writing and spin-orbit coupling (SOC) for magnetic state reading, can be directly cascaded and is regarded as a compelling alternative to CMOS toward 100-mV logic^8,9^. In a MESO logic, a single magnetic layer is used to store bit data, as well as couple writing block (ME block) and reading block (SO block)^8,10^. To date, the quasi-static ferroelectric switching of ME writing module has been achieved down to about 150 mV using ultrathin La-doped BiFeO_3_, with a pathway to get down to 100 mV^8,11,12^. However, there is a notable challenge that the spin-to-charge conversion (SCC) voltage of readout module is far less than 100 mV, which greatly restricts the cascading function of MESO logic. Typically, this signal measures in the tens or hundreds of milliohms, which results in an output voltage of less than 20 μV^7,13^. The stark discrepancy between the voltages required for writing and those available for reading presents a wide gap to the practical implementation of MESO logics. Although it is predicted using emerging quantum materials such as topological insulators (TIs) and two-dimensional electron gases (2DEGs) could enlarge the SCC signal due to their high SCC efficiency^14–19^, experimental results at room temperature (R.T.), to date, have not met the anticipated expectations^20,21^. In addition, the processing compatibility for massive production of these quantum materials has not been totally addressed. Therefore, more brand-new enhancement mechanism of output signal of MESO, different from utilizing high SOC materials, is highly desired to promote the MESO technology towards practical applications.

**Fig. 1:**
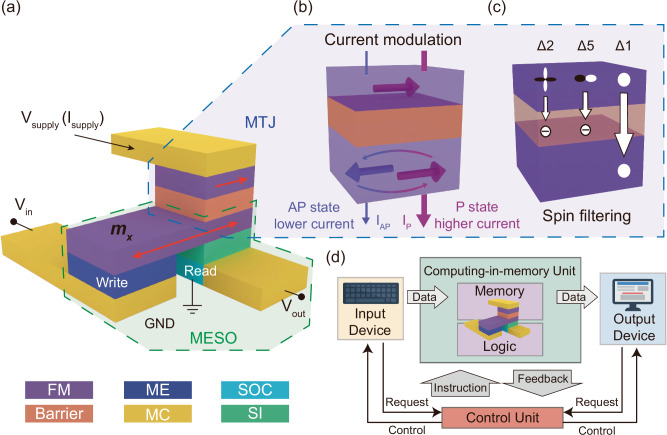
Concept of MTJ-enhanced MESO logic. **a** Structure schematic of MTJ-enhanced MESO. The device formed with a magnetoelectric (ME) layer, two ferromagnetic (FM) layers, a spin injection (SI) layer, a spin-orbit-coupling (SOC) layer, a tunnel barrier and metal connect (MC). **b** Principles of tunneling current modulation by MTJ resistance. The tunneling current shrinks when MTJ changes from P state to AP state. **c** Principles of spin filtering effect, where ∆_1_ band electron with nearly 100% spin polarization is allowed to be injected. **d** Data-exchange architecture of “MTJ + MESO” schemes.

Here, we propose for the first time the integration of magnetic tunnel junction (MTJ) and MESO by sharing one ferromagnetic (FM) layer as illustrated in Fig. 1a. The proposed device characterizes the same writing process as conventional MESO logic, i.e., V_in_ charges the capacitor of multiferroic material (depicted in dark blue) and when the voltage on ME layer exceeds its threshold, the ferroelectric polarization, interface exchange bias between ME layer and FM layer, and magnetization of FM layer switch in sequence. MTJ in proposed device acts as an amplifier in reading process, where two different magnetic state of free layer is converted into separated transverse charge signal V_out_. As shown in Fig. 1b, on the one hand, the injection tunneling current experiences a sudden increase upon the transition of MTJ from antiparallel (AP) to parallel (P) state under a constant voltage supply V_supply_, i.e., from I_AP_ to I_P_. This current alteration can induce an extra voltage signal superposed to the SCC output signal, and amplify the output signal of MESO. On the other hand, the high spin polarization (SP) rate effectuated by the spin filtering mechanism inherent in CoFeB/MgO/CoFeB sandwiched-structure can improve the SCC output voltage. As shown in Fig. 1c, Δ_1_ band electrons in sandwiched CoFeB/MgO/CoFeB tunnel easily when magnetizations of two CoFeB layers are parallel alignment, and hard when they are antiparallel, while Δ_2_ and Δ_5_ band electrons count little^22–25^. Consequently, tunneling current across an MTJ could be highly polarized to significantly enhance SP rate. In addition, we also simplified the MTJ-enhanced MESO logic device into an equivalent circuit to analyze the its operation in the cascaded structure and clarify the writing process in circuit level (Supplementary Note 1). Compared with other MTJ-integrated spin logic devices (e.g., all spin logic and domain-wall-based logic) which utilize spin torques to control input and flowing charges to transmit information, MTJ-enhanced MESO logic is more energy-efficient thanks to ultralow voltage operation^26–28^.

Thanks to the current modulation and spin filtering by MTJ, as well as high performance W SOC channel, our experimental findings at R.T. reveal a giant output signal, which is significantly enhanced to an impressive 1.5 mV, about two orders of magnitude greater than that reported in previous literatures^7–10,13,21^. Furthermore, the integrated MTJ can function as a cache memory to provide infinite bandwidth for MESO logic as shown in Fig. 1d, addressing the data exchange issue of computing unit and external memory from the perspective of architecture level, a problem that has been overlooked in previous researches. The proposed “MESO + MTJ” scheme can enhance the output signal and optimize the design architecture, offering a promising direction for future development of MESO technology.

## Results

### Structural, magnetic and electrical properties

**Fig. 2:**
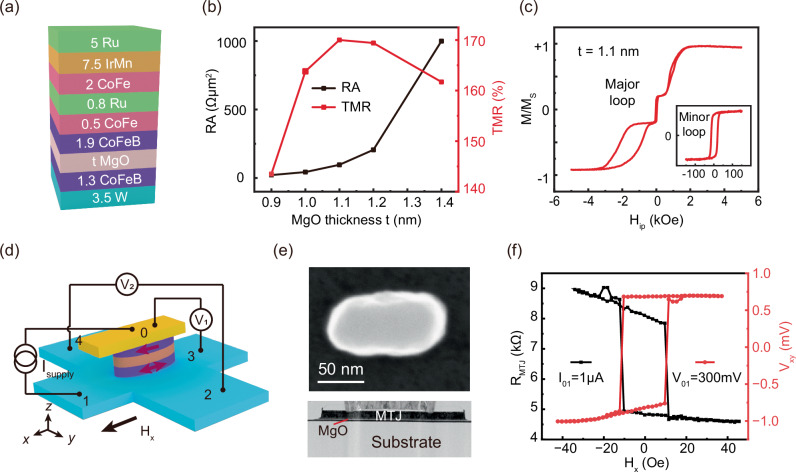
Structure optimization and basic device properties. **a** Schematic of multilayer film stacks. All numbers are in nm with varied MgO thickness t. **b** RA and extracted film TMR ratio as a function of MgO thickness t. **c** In-plane hysteresis loop of stacks in (**a**) with 1.1 nm MgO. Inset: minor loop of free layer behavior. **d** Schematic of MTJ-enhanced reading module and device measurement set-up, where TMR and SCC signal are simultaneously obtained. **e** Top view (upper panel) of a 60 nm × 130 nm junction and side view (lower panel). The well-crystallized MgO can be easily recognized. **f** Junction resistance under 1 μA supply current and transverse voltage under 300 mV supply voltage versus x axis magnetic field along.

At the outset of our experiments, we examined the impact of the MgO layer thickness on the tunneling magnetoresistance (TMR) ratio, which reveals film quality and SP rate. The functional stacks depicted in Fig. 2a, consisting of W (3.5)/CoFeB (1.9)/MgO (t)/CoFeB (1.9)/CoFe (0.5)/Ru (0.8)/CoFe (2)/IrMn (7.5)/Ru (5), are deposited on bottom electrode Ta (25)/CuN (20) (numbers in parentheses denotes film thickness in nm) with varying MgO thickness *t*. After post annealing at 400°C, we determined resistance-area product (RA) and TMR by using current in-plane tunneling (CIPT) measurement at R.T. The results are shown in Fig. 2b. The RA value increases with increasing MgO thickness, while the TMR ratio exhibits an initial increase followed by a decrease, reaching its maximum when the MgO thickness is 1.1 nm. This optimal TMR value suggests a higher quality of the film and a greater degree of SP^29^. Note that, TMR is expected to exhibit oscillations as ***t*** keeps increasing due to coherent tunneling and superposition effect of wave functions^30–32^.

We then selected a 1.1 nm MgO layer for subsequent experiments, and deposited MTJ stacks directly on thermally oxidized silicon substrate without Ta (25)/CuN (20). Figure 2c illustrates the hysteresis loop of full functional stacks deposited on thermally-oxidized silicon substrate using vibrating sample magnetometer. By applying an in-plane magnetic field along x direction, distinct magnetic behaviors were observed. The minor loop inset in Fig. 2c indicates that the coercive field of the free layer is approximately 10 Oe.

After a series of electron beam and ultraviolet lithography, as well as etching steps, we have successfully fabricated a junction device featuring multiple electrodes. Figure 2d illustrates the schematic of proposed MTJ-enhanced MESO readout block. The device features functional layers of two FM, a SOC, and a tunneling barrier layers. A cross-shape bottom electrode is used to generate transverse output voltage which can be measured between lead 2 and 4, with power supply across lead 0 and 1^7^. Generally, this reading process can be divided into two distinct phases: the spin polarization phase and the SCC phase. During the spin polarization phase, a constant supply source, denoted as V_supply_ or I_supply_, is applied perpendicularly to the device stacks and injecting a spin polarized current^7^. The injection efficiency is characterized by SP rate. Typically, SP rates are approximately 0.37 for Ni, 0.45 for Co, and 0.43 for Fe, with NiFe and CoFeB exhibiting higher values of 0.48 and 0.53, respectively^33–35^. By applying an external magnetic field and sweeping it along x axis, vertical voltage V_1_ (V_MTJ_) and transverse voltage V_2_ (V_xy_) are both supposed to appear abrupt changes when free layer magnetization changes, indicating TMR and SCC output signals.

In Fig. 2e, the upper panel shows the top view a junction with an area about 60 nm × 130 nm, while the lower panel gives the side view of junction in which the MgO barrier can be recognized clearly. High-resolution transmission electron microscope image of MTJ stacks also resolves the continuous lattice fringes of MgO barrier, indicating good crystallization (Supplementary Note 2). TMR and SCC signals are shown in Fig. 2f. The low resistance state, corresponding to the alignment of the two magnetic layers at P state, is measured to be around ${R}_{{MTJ}}^{P}$ = 4.7 kΩ. Meanwhile, the high resistance state exhibits a resistance of around ${R}_{{MTJ}}^{{AP}}\,$= 8 kΩ, corresponding to AP state. Consequently, TMR ratio is calculated to be 70% according to the equation of ${TMR}=({R}_{{MTJ}}^{{AP}}-{R}_{{MTJ}}^{P})/{R}_{{MTJ}}^{P}\times 100\%$. The TMR loss compared with CIPT results can be attributed to damage during nanofabrication. Notably, there are some anomalous points when the external field is around ± 20 Oe, which may be caused by irregular shapes and probable defects and pinnings at the edge of the junction. Meanwhile, we measure transverse voltage V_xy_ versus H_x_ (V_xy_−H_x_ loop) under a constant voltage supply of 300 mV. As expected, there are abrupt changes in transverse signal V_xy_, precisely at the points where free layer magnetization reverses. Notably, the measurement value of V_xy_ always contains offset signal originating from device structural asymmetry and other undesirable factors. ∆V_xy_, representing the difference between two magnetic states, is more reasonable to evaluate the output voltage (detailed discussion in Supplementary Note 3). By utilizing MTJ modulation and high SP rate, the observed output voltage ∆V_xy_ at 300 mV supply voltage is approximately 1.5 mV. This result is at least two orders larger than those in previous reports (less than 15 μV in ref. ^7^ and 1.5 μV in ref. ^13^).

### SCC signal under current source

We performed output signal under a constant current source to precisely evaluate the SCC signal without resistance modulation. The SCC result is intrinsically guaranteed by the anomalous spin-polarized velocity arising from a momentum-space Berry phase of Bloch electrons^36^. Reciprocally, charge-to-spin conversion (CSC), the inverse process of the SCC, can also be assessed using the same device. The charge flowing along W channel would cause spin accumulation and diffusion at and cross interfaces, known as direct spin Hall effect (SHE)^36–39^, which can be detected by magnetic electrodes^7,40^. Through applying current across lead 2 and 4, and measuring voltage between lead 0 and 1, SHE can be evaluated as CSC signal.

**Fig. 3:**
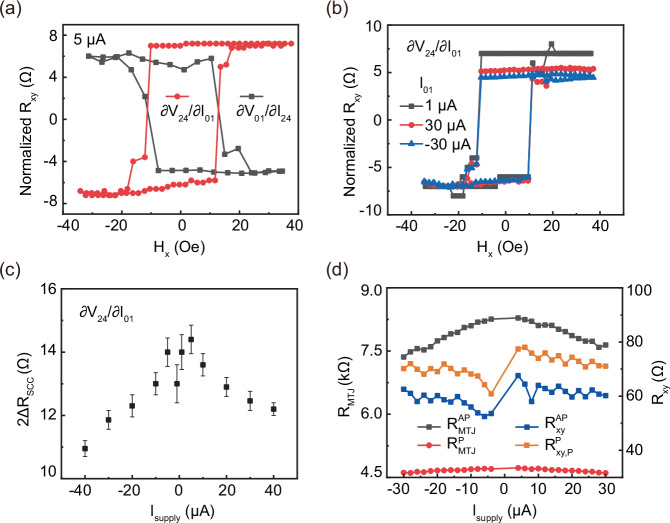
Output signal characterization under current source. **a** SCC signal $\partial {V}_{24}/\partial {I}_{01}$ and its reciprocal CSC signal $\partial {V}_{01}/\partial {I}_{24}$ as a function of external field with 5 μA supply current. **b** Normalized transverse resistance versus external field under different supply current. **c** 2∆R_SCC_ as a function of supply current. **d** MTJ resistance and transverse output resistance at different state as a function of supply current.

In Fig. 3a, we present the SCC curve $\partial {V}_{24}/\partial {I}_{01}$ and its inverse, CSC curve $\partial {V}_{01}/\partial {I}_{24}$ at 5 μA supply current. The CSC curve appears with opposite polarity to SCC curve, consistent with previous reports^7,40^. This indicates a giant SCC signal *2∆R*_*SCC*_ around 14.5 Ω, which is nearly 50 times larger than previous reports (0.3 Ω)^7^. However, the CSC signal is about 11 Ω, slightly smaller than SCC signal. We attribute this difference to the spin diffusion and relaxation in z-direction, compared with spin drift in SCC situation^7,40^. The measurement set-up in Fig. 2d also brings various Hall signals mixing in the transverse voltage V_xy_, such as conventional direct Hall effect, planar Hall effect and anomalous Hall effect^41,42^. Details are discussed in Supplementary Note 4. These Hall signals count less than 0.1 Ω in our nanodot structure, and affect little for the evaluation of final output signal.

The spin polarization phase and the SCC phase are both theoretically bias dependent. At first, the spin injection facilitated by an MTJ could be modulated by its bias-dependent SP^24^. Moreover, considering the ISHE mechanism in SCC phase, the transverse voltage loop of V_2_ shall invert polarity when the supply current direction is reversed, that is, ${V}_{2}({-I}_{{supply}},{H}_{x})={-V}_{2}({I}_{{supply}},{H}_{x})$, resulting no polarity change in resistance versus field curve, that is, ${R}_{{xy}}({-I}_{{supply}},{H}_{x})={R}_{{xy}}({I}_{{supply}},{H}_{x})$^43^. As a result, if one obtains ${R}_{{xy}}({-I}_{{supply}},{H}_{x})={-R}_{{xy}}({I}_{{supply}},{H}_{x})$, this result may be dominated by a current-symmetric term rather than SCC. The second is normally neglected by most researches, but it plays a significant role in SCC signal assessment.

Therefore, we measured different SCC signals by applying currents of varying amplitude and direction, to investigate the bias impact. The SCC signals at ±30 μA appear with the same polarity as expected, but are approximately 2.5 Ω lower than that at 1 μA, as shown in Fig. 3b. Moreover, the signal at +30 μA seems larger than signal at −30 μA. A detailed examination reveals a significant degradation in the SCC signal as supply current is increased, as depicted in Fig. 3c. Interestingly, similar phenomena have been previously reported in W/MgO/CoFeB tri-layer spin-tunneling structure^40^. The maximum SCC signal of *2∆R*_*SCC*_ is around 14.5 Ω at 5 μA supply. Obviously, when the current amplitudes are equal, the signal obtained under negative bias is slightly smaller than that under positive bias. These observations may imply a bias-dependent SP rate. That is to say, positive and negative biases activate different energy ranges of electronic states for tunneling, and regulate the effective barrier height at the two interfaces, amplifying disparities in interface transmission probabilities^44,45^.

Consequently, we explored the bias current dependence of the junction resistance R_MTJ_, and transverse resistance R_xy_. We use ${R}_{{MTJ}}^{P}$ (${R}_{{MTJ}}^{{AP}}$) and ${R}_{{xy}}^{P}$ (${R}_{{xy}}^{{AP}}$) to represent the junction and transverse resistances when MTJ is at P (AP) state, respectively. The findings are summarized in Fig. 3d, where it is noted that while ${R}_{{MTJ}}^{{AP}}$ exhibits a pronounced variation with bias current, ${R}_{{MTJ}}^{P}$ remains relatively unchanged. For the transverse resistance R_xy_, both ${R}_{{xy}}^{P}$ and ${R}_{{xy}}^{{AP}}$ exhibit monotonically decreasing trend with the increasing supply current, suggesting an additional mechanism between the SCC and TMR signals. Generally, the SCC signal in our MTJ-enhanced readout module contain two components of information, i.e. energetic electrons contribution from reference layer and equilibrium electrons mostly from free layer^40^. The energetic electrons are more energy-sensitive and thus attribute to the bias-dependent signal. These analyses collectively suggest that the utilization of highly spin-polarized electrons tunneling from CoFeB/MgO/CoFeB structures holds greater promise than those from a single FM layer in the development of spin-injection-based devices.

### MTJ-enhanced output signals under voltage source

**Fig. 4:**
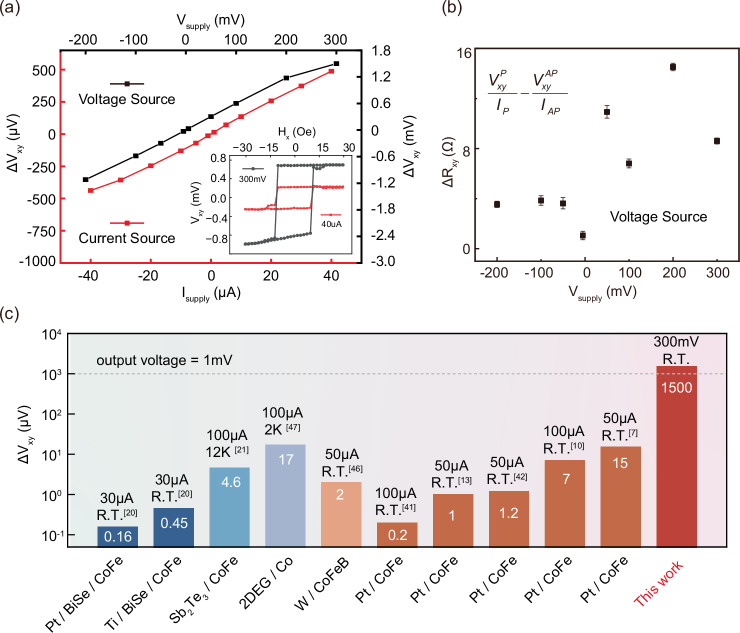
Output signals under different sources. **a** Output voltage comparison between voltage supply and current supply. Inset: Transverse voltage under 300 mV v.s. that under 40 μA. **b** Resistance outputs at different supply voltages. (**c**) Benchmark of output signals of MESO-like device for different materials and configurations until now.

Then, we compare the output voltage ∆V_xy_ under current source and voltage source. When the applied current source is substituted with a constant voltage V_supply_, the injection current would experience a sudden decrease upon the transition of MTJ from P to AP state.This alteration consequently affects the output voltage ∆V_xy_. As illustrated in the inset of Fig. 4a, the maximum observed output voltage ∆V_xy_ is approximately 1.5 mV corresponding to a 300 mV supply voltage, while the largest ∆V_xy_ for current source is about 0.4 mV for 40 μA supply. For a supply voltage of 300 mV, the tunneling current under the P state measures approximately 51 μA. Note that the corresponding current density (6.5 × 10^9^ A/m^2^) is far away from the critical value to induce spin-transfer-torque switching of magnetization in our devices. Given that ∆V_xy_ is proportional to the amplitude of the injection tunneling current, under a supply current of 51 μA, ∆V_xy_ is estimated to be around 0.51 mV, also significantly lower than 1.5 mV. These findings effectively validate the regulation impact of TMR effect when the device operates under a voltage source.

A comprehensive list of output voltages for various applied currents and voltages is provided in Fig. 4a. It is observed that larger applied source generates larger output voltage, and the voltage source can yield larger output voltage than current source when the current flow is comparable. Moreover, we could calculate the transverse resistance difference under voltage source. As shown in Fig. 4b, the relationship between ∆R_xy_ and voltage supply exhibits no regularity, quite different from that depicted in Fig. 3c. This is because of offset Hall voltage. When this offset is subtracted, ∆R_xy_ trend vs. voltage supply is similar with Fig. 3c, but with larger value (see Supplementary Note 5), meaning an extra term brought by TMR signal, in agreement with our analysis.

To clarify the regulation impact of MTJ, we calculate the relationship between ∆V_xy_ and MTJ parameters under a voltage source as follows,

1 $$\triangle {V}_{{xy}}=\frac{(1+{TMR}){R}_{{xy}}^{P}-{R}_{{xy}}^{{AP}}}{{R}_{{MTJ}}^{{AP}}}{V}_{{supply}}$$

The detailed formula derivation process can be found in Supplementary Note 6. For an MTJ, the parameter I_AP_ is directly determined by the thickness of the MgO barrier. Meanwhile, the TMR ratio critically depends on the overall quality of the MTJ structure. Therefore, to achieve a substantial increase in ∆V_xy_ under a constant voltage source, it is essential to optimize two key factors: (1) the MgO barrier must be sufficiently thin to enhance tunneling efficiency, and reduce ${R}_{{MTJ}}^{{AP}}$; (2) the crystalline quality of the MTJ must be carefully optimized to ensure large TMR ratio. These demands prefer MgO thickness at first peak of TMR oscillation curve, which also brings maximum conversion efficiency from V_supply_ to V_out_ (see Supplementary Note 7).

Figure 4c lists some impressive progress in MESO-like readout modules reported so far, containing different SOC materials (heavy metals, TIs and 2DEGs)^7,10,13,20,21,46,47^. Some of them provide output resistance signal ∆R_xy_ with a given current I_supply_, but no output voltages ∆V_xy_. As ∆V_xy_ is more critical and essential for practical application, we derive their voltage signals as ∆V_xy_ = I_supply_×∆R_xy_ and make a comparison. Although TIs and 2DEGs may remain theoretical superiority thanks to topological surface states and Rashba surface, experimental outputs have no advantages compared with W or Pt based device. One possible reason is their topological state are not stable at R.T. W-based device outputs 3-times larger than Pt-based device as expected, when they are patterned with same size^47^. Interestingly, Pt-based SO device has been reported the most, yet its results vary greatly. The most possible reason is Pt’s resistivity in reports varies a lot, from tens of μΩ·cm to hundreds of μΩ·cm^7^. Among all of these proposals, our work not only outperforms all others by a significant margin, but also enhances the output voltage from the microvolt level to the millivolt level, marking a new record. Towards applications, short-term stability is important for electronic device. In our proposal, the measurement is based on magnetic state readout process, which is quite different from writing stability. The reading stability of our device is similar with that of MTJ, always much better than writing stability^48^. We carried out thousand times of hysteresis loop measurements during 30 days, and results indicate there is no performance degradation (Supplementary Note 8).

### Mechanism Explorations

In this part, we explored possible mechanisms and factors to induce the giant readout signal. As we mentioned in Part 2.1, this reading process is divided into spin polarization phase and SCC phase. For SCC phase, the key factor is performance of W electrode. We performed spin-torque ferromagnetic resonance (ST-FMR) measurement on W/CoFeB (5 nm) heterojunction, with different W thickness (Supplementary Note 9). It was found that 3.5 nm W presents as large spin Hall angle as −0.26, promising for high SCC signal.

**Fig. 5:**
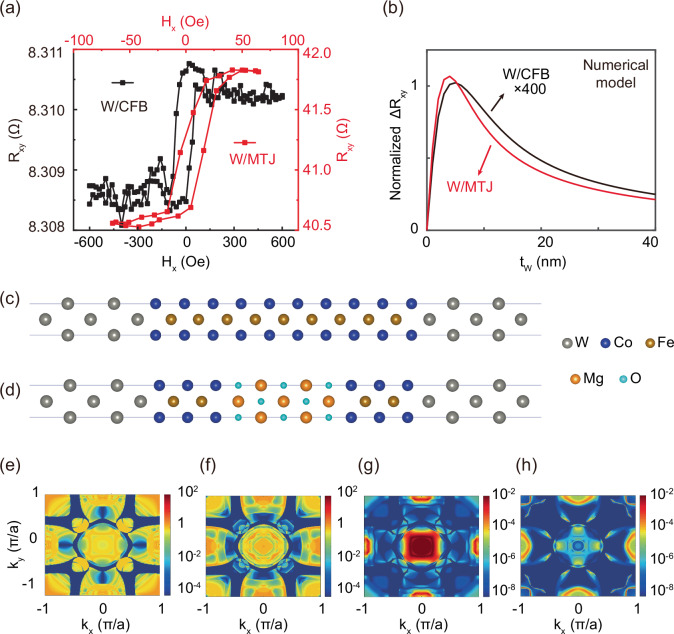
Control samples and mechanism analysis. **a** Output resistance signal comparisons between W/MTJ device and W/CoFeB-bilayer device using microdot device. **b** Numerical simulations for the bilayer and multilayer structure using physical parameters in experiments. **c**, **d** Layer structures of W/CoFe/W and W/CoFe/MgO/CoFe/W for ab-initio calculations. **e**, **f** Majority-to-majority and minority-to-minority transport for W/CoFe/W. **g**, **h** Majority-to-majority and minority-to-minority transport for W/CoFe/MgO/CoFe/W.

To investigate spin polarization phase, we compared the output transverse signal in proposed MTJ-enhanced device and traditional FM/SOC-bilayer device. We designed control experiments using 1 μm × 2.5 μm microdot devices with the same channel width of 3 μm. The output signals at I_supply_ = 10 μA of these two samples are shown in Fig. 5a. Sample 1 is a bilayer device composed of W (6)/CoFeB (5), yielding about 2.5 mΩ output signal (Details in Supplementary Note 10). Sample 2 is an MTJ-enhanced device with the above-mentioned film stack structure, which presents approximately 1.2 Ω signal. This difference is quite reasonable according to the numerical model^7^: (1) SP rate of sample 2 is about several times larger than that of sample1; (2) Because there is a phase change when the thickness of W changes from 6 nm to 3.5 nm, the W resistivity in sample 1 is about 60 μΩ · cm and that in sample 2 is about 367 μΩ · cm; (3) The θ_SH_ decreases for W from 0.26 at 3.5 nm to 0.04 at 6 nm^49,50^. These factors give about hundreds times of difference between these two scenarios, as depicted in Fig. 5b, quite matching the experiments. More details about the numerical model are presented in Supplementary Note 11.

To further explain the point about SP rates, we established W/CoFe/W and W/CoFe/MgO/CoFe/W atomic-level structure to perform calculation as shown in Figs. 5c, d. Detailed structure in the simulation is presented in Methods and Supplementary Note 12. K-resolved transmission spectra in Figs. 5e, f illustrate the majority-to-majority tunneling and minority-to-minority tunneling in W/CoFe/W, both generally spreading over the Brillouin zone. In contrast, majority-to-majority tunneling in W/CoFe/MgO/CoFe/W (P state) is concentrated within a square region around Γ (Fig. 5g), vanishing in the minority-to-minority spectrum (Fig. 5h). Given the definition of spin polarization rate ${SP}=({G}_{\uparrow \uparrow }^{\uparrow }-{G}_{\uparrow \uparrow }^{\downarrow })/({G}_{\uparrow \uparrow }^{\uparrow }+{G}_{\uparrow \uparrow }^{\downarrow })$, where ${G}_{\uparrow \uparrow }^{\uparrow }$ is the majority-to-majority spin conductance and ${G}_{\uparrow \uparrow }^{\downarrow }$ is the minority-to-minority spin conductance^51^, the calculated value gives about 92% SP rate for W/CoFe/MgO/CoFe/W structure, about 4 times larger than that for W/CoFe/W. It is evident that several monolayer MgO acts as spin filter to enlarge the difference between majority and minority spin transmission.

In addition, for measurements under voltage source V_supply_, MTJ not only increases SP, but also modulates injection current between I_AP_ and I_P_. The current difference

2 $$\triangle I={I}_{P}-{I}_{{AP}}={V}_{{supply}}\left(\frac{1}{{R}_{{MTJ}}^{P}}-\frac{1}{{R}_{{MTJ}}^{{AP}}}\right)={TMR}\cdot {I}_{{AP}}$$

brings different spin current injection to W channel and induces extra transverse output. This extra term directly enhances ∆V_xy_ and, after subtracting offset voltage, ∆R_xy_ is also enhanced (see Supplementary Note 5).

## Discussion

Our proposed “MESO + MTJ” scheme effectively overcomes key bottlenecks in MESO logic systems by simultaneously amplifying output signals and redefining data-exchange architectures. This dual advancement manifests in two pivotal aspects:

On the one hand, the output voltage can be further improved to 100 mV to enable the direct cascading of multiple MESO devices. According to Eq. 1, output voltage conversion efficiency ∆V_xy_/V_supply_ is highly related to MTJ properties. With higher TMR, and lower MTJ resistance at AP state, comes higher conversion efficiency. With proper stack design and interface optimization, TMR at R.T. of MTJ can be increased high up to 631%^31^. Meanwhile, high TMR ratio means high spin polarization of injection current. By adapting strong SOC material and scaling down the device size, the SCC process can be further improved. These methods are promising to improve the output voltages to the order of 100 mV.

On the other hand, the “MESO + MTJ” design addresses the critical issue of data exchange between computing unit and peripheral memory from device level (Supplementary Note 13). This device can operate as a logic unit with an intrinsically-integrated high-speed cache memory, as a shared free layer CoFeB is used for logic and memory simultaneously, which resolves the bandwidth mismatch between logic unit and external memory^52^. Moreover, it enables the extraction of any intermediate process results while simultaneously monitoring its resistance state during computation, which can improve the computation efficiency and reliability.

In addition, CMOS-compatible fabrication of our MTJ-enhanced MESO device is a key point before its practical application. Recently, La-doped BiFeO_3_ (LBFO) ME films have been successfully grown using magnetron sputtering^53^. Large exchange bias in LBFO/CoFeB heterostructure at room temperature and excellent surface roughness of LBFO have been demonstrated. Thus, in the future studies, the LBFO-based ME writing module and MTJ-enhanced readout module can be integrated together via micro-nano processing technology to construct MTJ-enhanced MESO logic.

In conclusion, our proposal offers a viable pathway toward advancing the real-world implementation of MESO logic technology.

## Methods

### Film growth and characterization

All the films used in this work were deposited on 8-inch thermally oxidized silicon substrate, using ultrahigh vacuum *dc/rf* magnetron sputtering system. The substrate was first pre-cleaned for 60 s by Ar^+^ ion beam. The CoFe, CoFeB, and IrMn denote Co_70_Fe_30_, Co_60_Fe_20_B_20_, and Ir_20_Mn_80_ alloy with nominal target compositions, respectively. After deposition, the stacks were in-situ annealed at 400 °C for 1 h under an external field of 1 T along x direction to define the reference layer direction and form highly oriented crystalline CoFeB/MgO/CoFeB MTJs. The static in-plane anisotropy magnetization behavior was examined using a vibrating sample magnetometer system.

### Device fabrication

For a nanoscale MTJ-enhanced spin-orbit device, we first patterned 500 nm × 2.5 μm cross-shape channels using standard electron beam lithography (EBL) with maN2403 negative tone photoresist and Ar^+^ ion beam etching (IBE). During milling, we monitored the secondary-ion mass spectra. After that, a second round of EBL and IBE procedures were used to define elliptical junctions. Thanks to high-resolution and high-aspect-ratio negative tone photoresist, we successfully formed a photoresist pillar with tens-nanometer length and hundreds-nanometer height. The junction etching was divided into two phases: first, we defined its appearance by small angle (15°) etching until the bottom 3.5-nm-thick W signal appears; second, large angle (75°) etching was utilized to meticulously clean the sidewall re-depositions across the barrier. Rather than removing the photoresist immediately, we covered the pillars with a 50-nm-thick silicon nitride (Si_3_N_4_) passivation layer, by using inductively-coupled-plasma chemical vapor deposition, and then followed by lift-off process. Vias through bottom electrodes were formed by standard ultraviolet lithography and inductively coupled plasma reactive ion etching. Ti (10 nm)/ Au (100 nm) electrodes are finally formed by E-beam evaporation (EBE).

For the microscale SO device, i.e., CoFeB/W readout device and CoFeB/MgO/CoFeB/W MTJ-enhanced readout device, standard ultraviolet lithography and etching process were used to pattern the bottom electrode and the microdot pillar. Then, 50-nm-thick Si_3_N_4_ passivation layer covered the pillars, and followed by lift-off process. Finally, Ti (10 nm)/Au (100 nm) electrodes are formed by EBE. For bilayer ST-FMR measurement, the device was formed by standard ultraviolet lithography and etching process, with Ti(10 nm)/ Au(100 nm) electrodes using EBE and lift-off process.

### Device characterization

Electronic transport measurements of MTJ-enhanced readout device were conducted on our self-developed probe station, using a Keithley 2182 nanovoltmeter and a Keysight B1500A semiconductor device parameter analyzer under varying in-plane magnetic field. The B1500A is used for direct resistance measurement while 2182 nanovoltmeter is more sensitive and used for transverse voltage measurement. ST-FMR measurement of the bilayer heterojunction was performed using a Keysight MXG N5183B analog signal generator and SR830 lock-in amplifier.

### Ab-initio simulation

The atomic structures are relaxed by Vienna Ab-initio Simulation package (VASP) so that the residual forces are minimized under 0.01 eV/Å^54^. Next, transport calculations are performed by NanoDCAL, which is based on density functional theory (DFT) and Keldysh non-equilibrium Greens function (NEGF)^55^. The physical quantities are represented using a linear combination of atomic orbitals (LCAO) basis with double-zeta plus polarization (DZP) functions. A 20 × 20 × 1 k-point mesh is employed in the self-consistent calculation, with the Hamiltonian matrix converged to a tolerance of 10^−4^ eV. Next, the spin-resolved conductance calculation of 500 × 500 × 1 was performed to calculate TMR and polarization. The Perdew-Burke-Ernzerhof generalized gradient approximation (PBE-GGA) is selected to describe the exchange-correlation potential, and the cutoff energy of the real space grids is fixed as 3000 eV.

## Supplementary information

Supplementary Information

Transparent Peer Review file

## Data Availability

The data that support the plots within this paper and the other findings of this study are available from the corresponding author upon reasonable request.
