# Supplementary material for: Giant spin-orbit magnetic state readout enhanced by a magnetic tunnel junction
Source: Nat Commun. 2026 May 26;17:6838. doi: 10.1038/s41467-026-73382-9 (PMC13388924; doi:10.1038/s41467-026-73382-9)
Supplement: Supplementary file 1 — Supplementary Information [file 41467_2026_73382_MOESM1_ESM.pdf]

Supplementary Information

Giant Spin-Orbit Magnetic State Readout Enhanced by a Magnetic Tunnel Junction

Yan Huang[1,2,3,†], Kun Zhang[1,2,3,†,], Guo Liu[1,2,†], Xiaobai Ning[1,2,†], Shiyang Lu[2,3,†], Shijie Xu[1,2,†], Qing Yang[2,4,†], Wenlong Cai[2], Renyou Xu[2], Yuxuan Yao[2], Yu He[2], Jinkai Wang[1,2], Bo Li[2], Haozhe Yang[2], Kewen Shi[2], Kaihua Cao[2,3], Chao Zhao[2], Yue Zhang[1,2,3,*], and Weisheng Zhao[1,2,3,*]*

[1]State Key Laboratory of Spintronics, Hangzhou International Innovation Institute & School of Integrated Circuit Science and Engineering, Beihang University, Hangzhou 311115, P. R. China.

[2]Fert Beijing Research Institute, MIIT Key Laboratory of Spintronics, School of Integrated Circuit Science and Engineering, Beihang University, Beijing 100191, P. R. China.

[3]Integrated Circuit and Intelligent Instruments Innovation Center, Qingdao Research Institute, Beihang University, Qingdao 266101, P. R. China.

[4]State Key Laboratory of Spintronics Devices and Technologies, School of Integrated Circuits, Nanjing University, Suzhou 215163, P. R. China.

[†]These authors contribute equally to this work.

[*]Correspondence to zhang_kun@buaa.edu.cn; yz@buaa.edu.cn; weisheng.zhao@buaa.edu.cn

Table of Contents

Supplementary Note 1. Equivalent circuit diagram of MTJ-enhanced MESO logic device

Supplementary Note 2. High-resolution transmission electron microscope image of MTJ stacks

Supplementary Note 3. Offset voltage in Hall-like measurements

Supplementary Note 4. Evaluation of various Hall effects

Supplementary Note 5. Impacts of offset Hall voltage under voltage source

Supplementary Note 6. Derivation process of MTJ-modulation effect

Supplementary Note 7. Discussions on supply-out conversion efficiency

Supplementary Note 8. Characterization of short-term stability

Supplementary Note 9. Characterization of spin Hall angle of W

Supplementary Note 10. Details of bilayer microdot readout device

Supplementary Note 11. Detailed description of the numerical model

Supplementary Note 12. Detailed structure of MTJ in the simulation

Supplementary Note 13. Processing logic using "MTJ+MESO" unit

Supplementary Note 1. Equivalent circuit diagram of MTJ-enhanced MESO logic device

The equivalent circuit of MTJ-enhanced MESO logic is shown in **Supplementary Fig. 1**. The core components contain capacitor of ME layer C_{ME}, resistance of MTJ R_{MTJ}, controlled current source that converts vertical current I_{supply} to horizontal current I_{SOC}. $R_{connect1}$ and $R_{connect2}$ denote connection resistance, R_{FM} denotes FM layer resistance between ME and SO modules, and $R_{SOC,v}$ and $R_{SOC,h}$ denote vertical and horizontal resistance of spin-orbit coupling (SOC) layer. V_{out} representing the two different magnetic state of free layer is evaluated by the parameter ΔV_{xy}, generated by inherently spin to charge conversion (SCC) mechanism and connected to next level V_{in}. V_{out} is separated with GND by SOC layer which functions as controlled current source with finite internal resistance. In cascaded devices, the voltage (V_{in} - V_A) applied to ME, is equal to (V_{out} - V_A). Due to SOC and inverse spin Hall effect, when FM is spin right, for example, the converted charge flows forward, leading to positive cascading voltage (V_{out} - V_A), *i.e.* $+\Delta V_{xy}/2$, and When FM is spin left, the converted charge flows backward, leading to negative cascading voltage (V_{out} - V_A), *i.e.* $-\Delta V_{xy}/2$. Therefore, for an ideal device with no asymmetry, inhomogeneities and parasitic effects, the voltage between V_{out} and V_A is $-\Delta V_{xy}/2$ or $\Delta V_{xy}/2$.

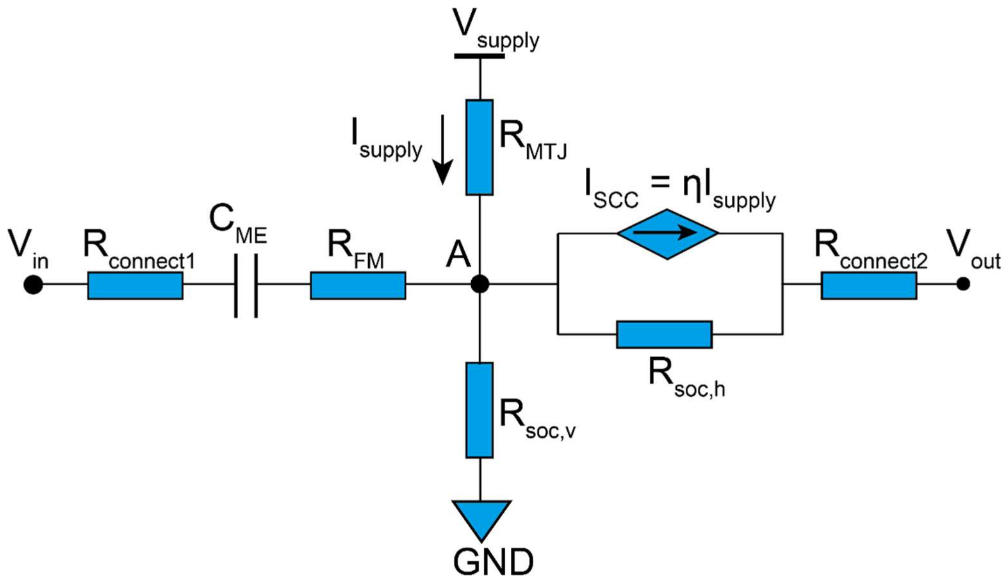

Supplementary Fig. 1. The equivalent circuit diagram of MTJ-enhanced MESO logic device.

Supplementary Note 2. High-resolution transmission electron microscope image of MTJ stacks

High-resolution transmission electron microscope (HRTEM) image of MTJ stacks is presented as **Supplementary Fig. 2**. The image clearly resolves the continuous lattice fringes of MgO barrier layer within the MTJ stack. Additionally, interfaces between different layers are well-defined.

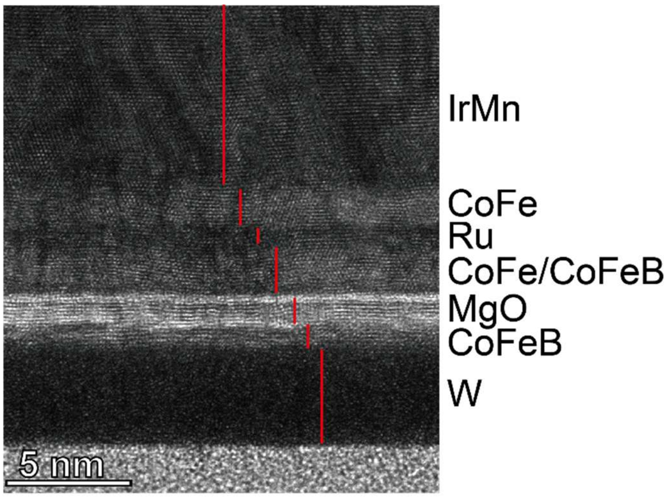

Supplementary Fig. 2. HRTEM image of MTJ stacks

Supplementary Note 3. Offset voltage in Hall-like measurements

The SCC measurement is a Hall-like measurement, which could bring offset voltage V_{offset} in results originating from device structural asymmetry, material/interface inhomogeneities, parasitic effects in the measurement system, and limitations of calibration methods[1]. The offset voltage is not related to magnetic state, and shall be excluded in final results.

We derive the expression of V_{offset} from equivalent circuit when ignoring various Hall effect. **Supplementary Fig. 3a** is the simplified measurement set-up, where current is injection from lead 0 to 1, and voltage is measured between lead 2 and 4. **Supplementary Fig 3b** is the equivalent circuit, where R_{ij} is the equivalent resistance between lead i and j. Let the conductance be $G_{ij} = 1/R_{ij}$ for etch resistance. According to Kirchhoff's law, we have

$$G_{01}(V_0 - V_1) + G_{02}(V_0 - V_2) + G_{04}(V_0 - V_4) = I_{supply} \quad \text{(S1)}$$

$$G_{12}(V_2 - V_1) + G_{02}(V_2 - V_0) - G_{24}(V_2 - V_4) = 0 \quad \text{(S2)}$$

$$-G_{24}(V_4 - V_2) + G_{04}(V_4 - V_0) + G_{14}(V_4 - V_1) = 0 \quad \text{(S3)}$$

As lead 1 is connected to GND, we have V1 = 0. Equations (S1) ~ (S3) can be simplified as

$$(G_{01} + G_{02} + G_{04})V_0 - G_{02}V_2 - G_{04}V_4 = I_{supply} \quad \text{(S4)}$$

$$G_{02}V_0 - (G_{02} + G_{12} + G_{24})V_2 + G_{24}V_4 = 0 \quad \text{(S5)}$$

$$G_{04}V_0 + G_{24}V_2 - (G_{04} + G_{14} + G_{24})V_4 = 0 \quad \text{(S6)}$$

Let the conductance matrix be **G**, voltage vector **V** = $[V_0\ V_2\ V_4]^T$, **I** = $[I_{suppy}\ 0\ 0]^T$. The system is **GV= I**, where

$$\mathbf{G} = \begin{bmatrix} G_{01} + G_{02} + G_{04} & -G_{02} & -G_{04} \\ G_{02} & -(G_{02} + G_{12} + G_{24}) & G_{24} \\ G_{04} & G_{24} & -(G_{04} + G_{14} + G_{24}) \end{bmatrix}$$

Using Cramer's Rule, finally we have

$$V_0 = I_{supply}[(G_{02} + G_{12} + G_{24})(G_{04} + G_{14} + G_{24}) - G_{24}^2]/\det(\mathbf{G}) \quad \text{(S7)}$$

$$V_2 = -I_{supply}[G_{02}(G_{04} + G_{14} + G_{24}) + G_{04}G_{24}]/\det(\mathbf{G}) \quad \text{(S8)}$$

$$V_4 = -I_{supply}[G_{04}(G_{02} + G_{12} + G_{24}) + G_{02}G_{24}]/\det(\mathbf{G}) \quad \text{(S9)}$$

where $\det(\mathbf{G})$ is the determinant of **G**.

Offset voltage V_{offset} is given by

$$V_{offset} = V_2 - V_4 = I_{supply}[G_{04}G_{12} - G_{02}G_{14}]/\det(\mathbf{G}) \quad \text{(S10)}$$

When the device is ideal, G_{04} is equal to G_{02}, and G_{12} is equal to G_{14}, thus $V_{offset} = 0$, meaning there is no offset voltage. Otherwise, there exists offset voltage disturbing, and this disturb is independent of electron spins and magnetic state.

Now we take various Hall effects into consideration. The measurement voltage V_{xy} consists of various Hall voltage V_{Halls} and offset voltage V_{offset}, *i.e.* $V_{xy} = V_{Halls}+V_{offset}$. As V_{Halls} reflects the magnetic state. Thus, the difference between $V_{Halls,max}$ and $V_{Halls,min}$ (denoting two different magnetic states) is critical for cascading. As $\Delta V_{xy} = V_{xy,max} - V_{xy,min} = (V_{Halls,max}+V_{offset}) - (V_{Halls,min}+V_{offset}) = V_{Halls,max} - V_{Halls,min}$, we characterize ΔV_{xy} as key parameter to evaluate the output.

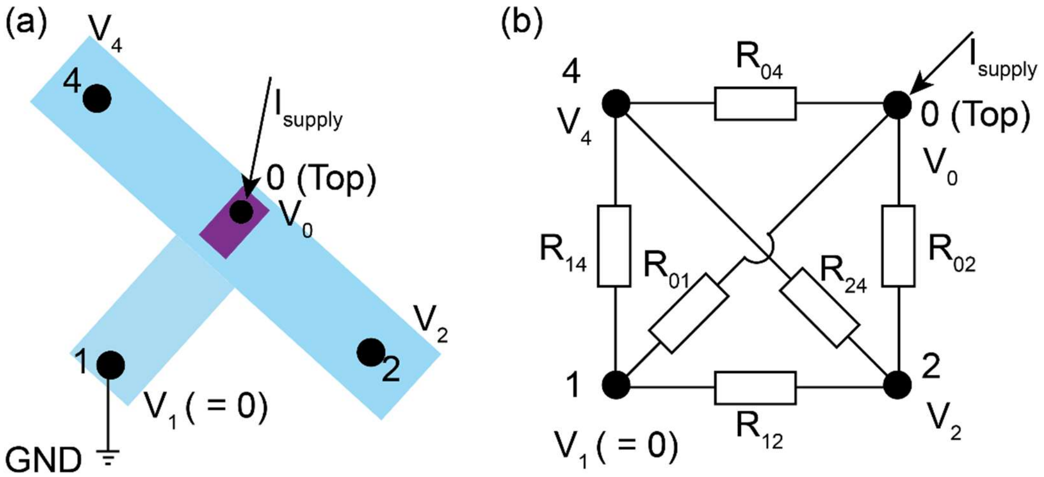

Supplementary Fig. 3. Measurement diagram and equivalent circuit. (a) Schematic of measurement. (b) equivalent circuit of measurement diagram.

Supplementary Note 4. Evaluation of various Hall effects

Our multiterminal device facilitates the comprehensive evaluation of various Hall effect signals. We recall this sketch in **Supplementary Fig. 4a**, and inset shows the top view of the cross W-channel. At first, the conventional direct Hall effect (DHE) voltage arises in the W layer influenced by the out-of-plane (OOP) stray field arising from free layer CoFeB, as shown in **Supplementary Fig. 4b**. When free layer magnetization switches, the OOP stray field is inversed, inducing DHE voltage to reverse[2]. We assess DHE by applying a current from lead 1 to 3 with voltage measured between lead 2 and 4. The DHE result (V_{24} in **Supplementary Fig. 4e**, red curve) shows no distinct steps during the magnetization switching of CoFeB free layer with magnetic field, indicating a negligible DHE signal.

Secondly, the planar Hall effect (PHE), arising from SOC in magnetic materials, where the magnetization direction distorts the electron orbitals and creates an anisotropic scattering environment. As a result, electrons traveling through the material are deflected toward the direction of least resistance—typically perpendicular to the magnetization axis—leading to a transverse voltage, as shown in **Supplementary Fig. 4c**. PHE components are observed in V_{24} as peaks in **Supplementary Fig. 4e** (red curve, @around 13 Oe). The PHE affects the evaluation of the spin-to-charge conversion (SCC) signal when we focus on field region of -20 Oe ~ +20 Oe, but are deemed non-impactful when the field exceeds H_c and thus can be ignored[3]. The bottom electrode resistance R_{BE} fluctuates near the average value 2275 Ω and 2260 Ω for R_{13} (black curve) and R_{24} (blue curve), respectively.

Moreover, anomalous Hall effect (AHE), due to the symmetry between OOP injection current and in-plane magnetizations, as shown in Supplementary Fig. 4d, which can be evaluated by numerical model or finite element simulation[3]. We measured its resistivity ρ_{AHE} at first, from the Hall signal by sweeping OOP field between ±2.5T, using a full-film cross device. The result is shown in **Supplementary Fig. 4f**. We evaluated the upper boundary of ρ_{AHE} by attributing the AHE resistance difference ($2\Delta R_{AHE}$=2 Ω) totally to the bottom 1.9-nm-thick CoFeB and derived a value of $\rho_{AHE}=\Delta R_{AHE}\cdot t_{CoFeB}$= 0.19 μΩ·cm, comparable with previous study[3]. Considering ρ_{CoFeB} is about 70 μΩ·cm in our study, the anomalous Hall angle

is less than 1%. These parameters would give a negligible influence (less than 70 mΩ) compared with the total output resistance signal presented in the main text.

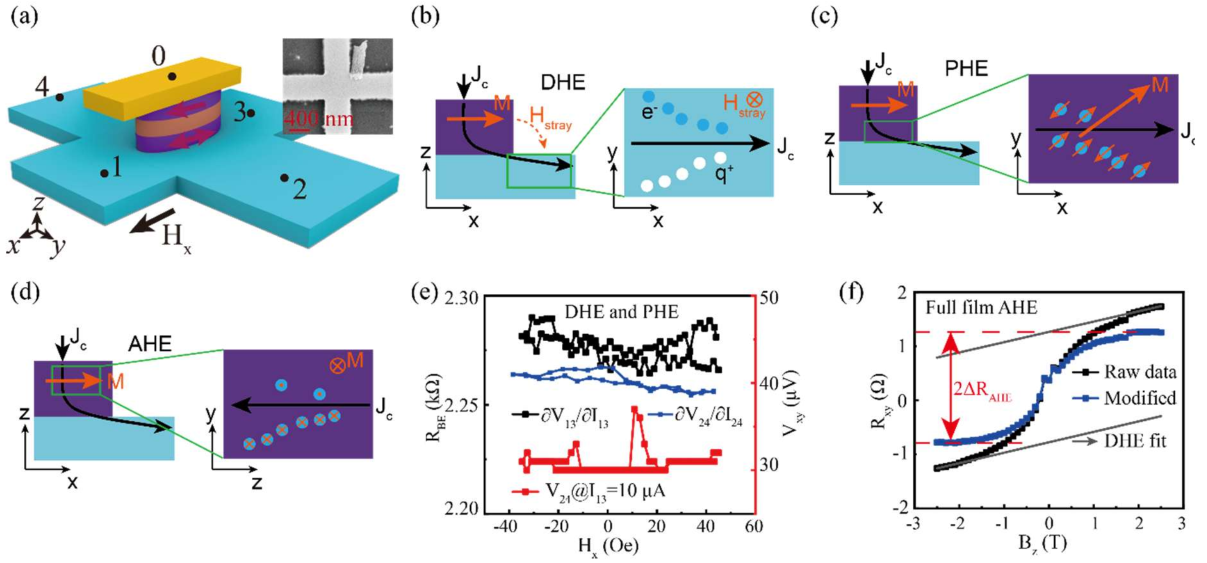

Supplementary Fig. 4. Evaluation of various Hall effects. (a) Device sketch. Inset: top view of the cross-W channel. (b) Schematic diagram of AHE. (c) Schematic diagram of PHE. (d) Schematic diagram of AHE. (e) DHE and PHE signal (red curve), as well as bottom electrode resistance (black and blue curve). (f) AHE signal of full film.

Supplementary Note 5. Impacts of offset Hall voltage under voltage source

ΔV_{xy} and ΔR_{xy} under voltage source as shown in **main text Figure 4a** and **Figure 4b (recalled as Supplementary Fig. 5a and Supplementary Fig. 5b)** are directly obtained using measurement raw data, where ΔR_{xy} contains offset voltage contribution as discussed **Supplementary Note 4**. Take data under 200 mV and -200 mV supply as example. Here, we show two different estimations. As shown in **Supplementary Fig. 5c**, raw transverse voltage data under 200 mV and -200 mV spreads across 1.2 mV to 2.8 mV and -3.6 mV to -2.0 mV, respectively, corresponding to ΔR_{xy} around 15 Ω@200 mV and 4 Ω@-200 mV (**Supplementary Fig. 5d**). However, when we exclude V_{offset} from raw data, the modified output transverse voltage spreads across -0.8 mV to 0.8 mV for both 200 mV and -200 mV supply, as shown in **Supplementary Fig. 5e**. As a result, the modified R_{xy} v.s. H_x loops are more closed for opposite voltage supply as shown in **Supplementary Fig. 5f**. The Corresponding modified ΔR_{xy} is 42.4 Ω@200 mV and 39.2 Ω@-200 mV. We show the modified R_{xy} v.s. H_x with supply voltage from -200 mV to 300 mV in **Supplementary Fig. 5g** and ΔR_{xy} trend vs supply voltage as **Supplementary Fig. 5h**. For modified Hall resistance signal, ΔR_{xy} curve is almost smooth for both positive and negative supply voltage. And, this trend is agreement with that shown in **Figure 3c** in the main text.

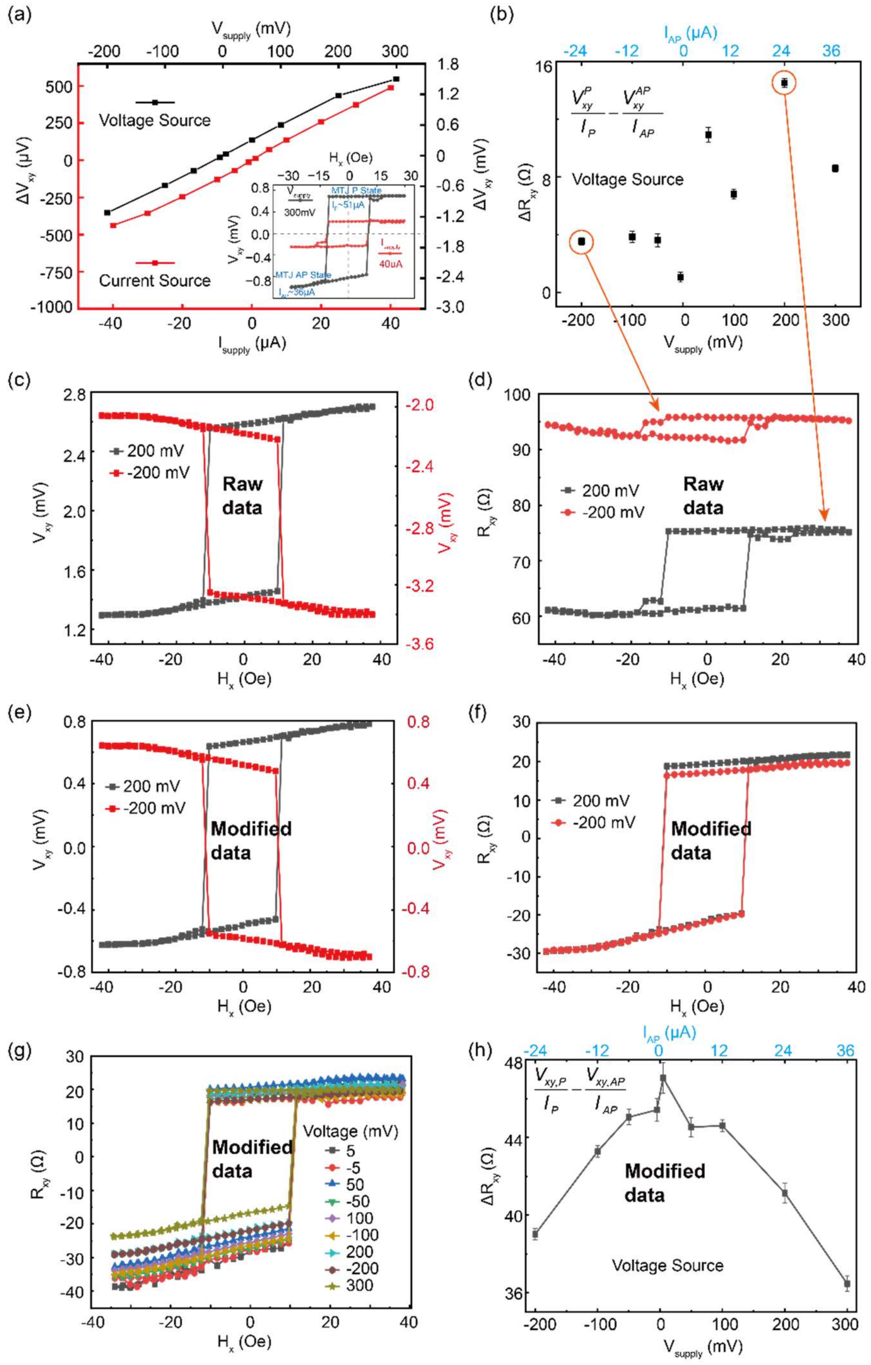

Supplementary Fig. 5. Offset Hall voltage influence. (a) Output voltage comparison between voltage supply and current supply. Inset: Transverse voltage under 300 mV v.s. that under 40 µA. (b) Resistance outputs from raw data at different supply voltages. (c) Raw data of transverse voltage v.s. field at ±200 mV supply. (d) Transverse resistance v.s. field from measurement raw data at ±200 mV supply. (e) Modified data of transverse voltage v.s. field at ±200 mV supply. (f) Transverse resistance v.s. field from modified data at ±200 mV supply. (g) Transverse resistance v.s. field at different supply voltages. (h) Resistance outputs from modified data at different supply voltages.

Supplementary Note 6. Derivation process of MTJ-modulation effect

Under a constant voltage source V_{supply}, the injection current under antiparallel (AP) and parallel (P) state of magnetic tunnel junction (MTJ) can be calculated as following:

$$I_{AP}=\frac{V_{supply}}{R_{MTJ}^{AP}},\ I_P=\frac{V_{supply}}{R_{MTJ}^{P}}$$

where R_{MTJ}^{AP} and R_{MTJ}^{P} are the resistance of MTJ under AP and P states.

Moreover, the transverse resistance output voltage ΔV_{xy} can be calculated by the following equation,

$$\Delta V_{xy}=V_{xy}^{P}-V_{xy}^{AP}=I_P R_{xy}^{P}-I_{AP}R_{xy}^{AP} \quad (1)$$

Here, R_{xy}^{AP} and R_{xy}^{P} represents the transverse resistance under AP and P states, respectively. Then, equation 1 can be transformed to the following,

$$\Delta V_{xy}=\frac{V_{supply}}{R_{MTJ}^{P}}R_{xy}^{P}-\frac{V_{supply}}{R_{MTJ}^{AP}}R_{xy}^{AP}$$

$$=V_{supply}\left(\frac{R_{xy}^{P}}{R_{MTJ}^{P}}-\frac{R_{xy}^{AP}}{R_{MTJ}^{AP}}\right)$$

$$=V_{supply}\frac{R_{xy}^{P}R_{MTJ}^{AP}-R_{xy}^{AP}R_{MTJ}^{P}}{R_{MTJ}^{P}R_{MTJ}^{AP}}$$

$$=\frac{R_{xy}^{P}R_{MTJ}^{P}+R_{xy}^{P}\left(R_{MTJ}^{AP}-R_{MTJ}^{P}\right)-R_{xy}^{AP}R_{MTJ}^{P}}{R_{MTJ}^{P}R_{MTJ}^{AP}}V_{supply}$$

$$=\left(\frac{R_{xy}^{P}-R_{xy}^{AP}}{R_{MTJ}^{AP}}+\frac{R_{xy}^{P}\left(R_{MTJ}^{AP}-R_{MTJ}^{P}\right)}{R_{MTJ}^{P}R_{MTJ}^{AP}}\right)V_{supply}$$

$$\left(\text{Considering that } TMR=\frac{R_{MTJ}^{AP}-R_{MTJ}^{P}}{R_{MTJ}^{P}}\right)$$

$$=\left(\frac{R_{xy}^{P}-R_{xy}^{AP}}{R_{MTJ}^{AP}}+\frac{R_{xy}^{P}TMR}{R_{MTJ}^{AP}}\right)V_{supply}$$

$$=\frac{(1+TMR)R_{xy}^{P}-R_{xy}^{AP}}{R_{MTJ}^{AP}}V_{supply}$$

Assuming that $\Delta R_{xy}=R_{xy}^{P}-R_{xy}^{AP}$, thus $\Delta V_{xy}=I_{AP}\Delta R_{xy}+TMR\cdot I_{AP}R_{xy}^{P}$. The first term is equal to the signal under a current source of I_{AP}, and the second term is an extra signal by MTJ current modulation.

Supplementary Note 7. TMR implications for supply-out conversion efficiency

Supply-out conversion efficiency η_I(for current source)/ η_V(for current source), defined as output current/voltage divide input current/voltage, is more critical and essential for practical application. In the readout module of MESO logic device, η_I is given by $\eta_I=\frac{I_{out}}{I_{supply}}\times100\%=\frac{\Delta V_{xy}/R_{24}}{2I_{supply}}\times100\%=\frac{\Delta R_{xy}}{2R_{24}}\times100\%$, where $R_{24}=\partial V_{24}/\partial I_{24}$ is resistance of bottom electrode. According to previous reports[4], $\Delta R_{xy}$is proportional to spin polarization rate SP, η_I thus increases with TMR increasing. Things are different for supply voltage source. Efficiency between output voltage and supply voltage for voltage source is defined as $\eta_V = \frac{\Delta V_{xy}/2}{V_{supply}} \times 100\% = \frac{(1+TMR)R_{xy}^{P}-R_{xy}^{AP}}{2R_{MTJ}^{AP}} \times 100\%$. To get higher η_V, one should employ stacks with higher TMR and lower R_{MTJ}^{AP}, which means using MgO thickness at first peak of TMR oscillation curve.

In conclusion, increasing TMR ratio can always increase the efficiency of MESO output under supply current source, but for voltage source, MESO output efficiency reaches maximum at first peak of TMR oscillation curve.

Supplementary Note 8. Characterization of short-term stability

At very beginning, it is necessary to declare that the measurement in the main text is a reading process for magnetic state, and the relevant property is reading stability. Different from writing stability, where the device state is measured every hundreds or thousands of stimulations by a constant large current/voltage, reading stability of our proposal is determined by performing hundred-times of hysteresis loop measurements. Short-term reading stability is always much better than writing stability, as stimulated voltage is lower[5].

In experiments, with power supply across lead 0 and 1 and sweeping in-plane field H_x, transverse voltage is measured between lead 2 and 4. Similarly, we monitored junction resistance as reference. We carried out 100 times measurements per day at one same device and repeated for 30 days. Notably, at first, we used current supply of 5 μA (for normal readout as other researchers did) and changed to voltage supply of 300 mV at day 16 (for maximum readout).

Supplementary Fig. 6a shows the short-term stability at supply current of 5 μA. At 1st day and 1st test cycle, MTJ resistance ($R_{MTJ} \sim H_x$) and transverse voltage ($V_{xy} \sim H_x$) versus H_x loop (denotes in black square) indicates $R_P \sim 4.75$ kΩ, $R_{AP} \sim 8$ kΩ, TMR~70%, transverse output voltage $\Delta V_{xy} \sim 0.07$ mV. At 15th day and 1500th test cycle, R_{MTJ} (V_{xy}) ~ H_x loop present little changes. **Supplementary Fig. 6b** shows the short-term stability at supply voltage of 300 mV. At 16th day and 1501th test cycle, R_{MTJ} (V_{xy}) ~ H_x loop indicates $R_P \sim 4.7$ kΩ, $R_{AP} \sim 7.2$ kΩ, TMR~53%, transverse output voltage $\Delta V_{xy} \sim 1.65$ mV. TMR decrease than that at 5 μA is attributed to bias dependent spin tunneling[6]. At 30th day and 3000th test cycle, $R_{MTJ} \sim H_x$ loop present little changes, and V_{xy} dropped by 0.01mV. This small drop can be attributed to measurement bias. **Supplementary Fig. 6c** presents the total trend of R_P, R_{AP} and ΔV_{xy} during 30-day measurements. R_{AP} experiences a drop at day 16, and ΔV_{xy} increases, because supply source is change from 5 μA to 300 mV. There is no obvious performance degradation after 3000 cycles test in 30 days, indicating the excellent stability.

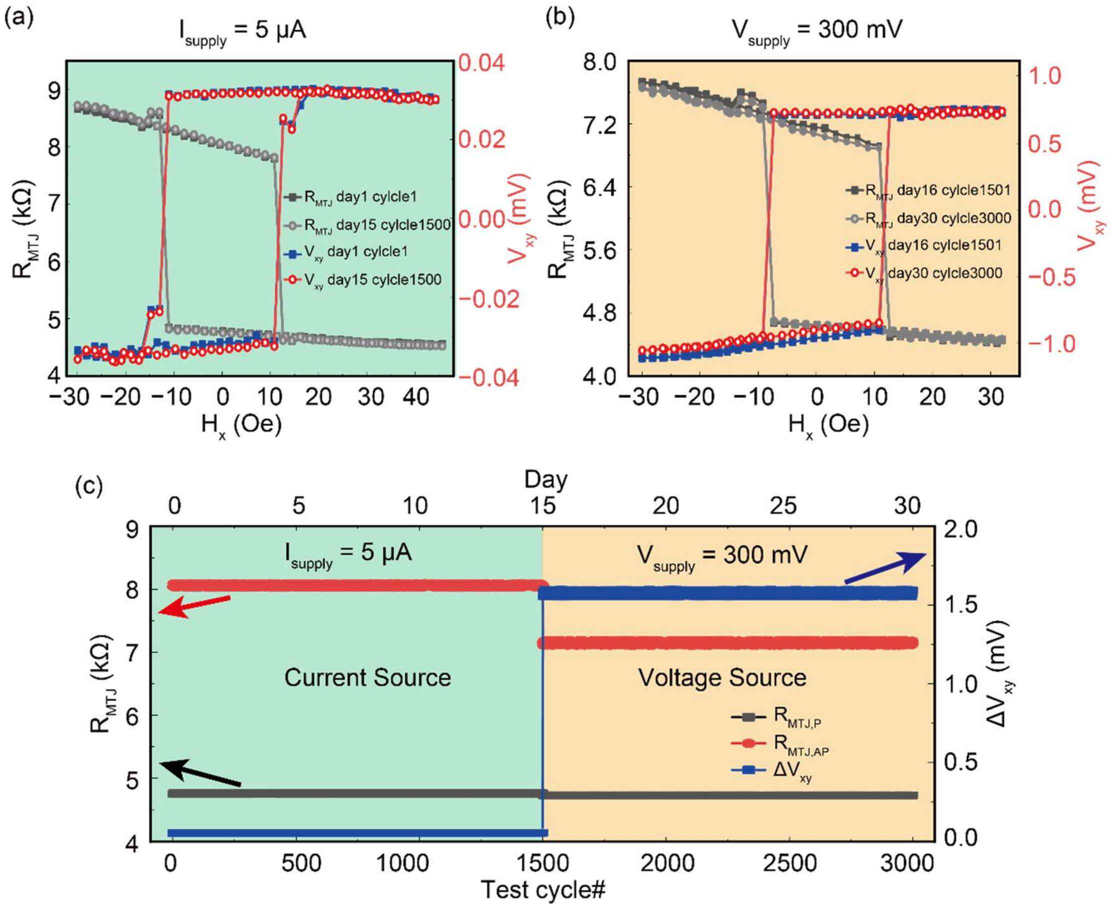

Supplementary Fig. 6. Evaluation of short-term stability. (a) Short-term stability at supply current of 5 μA. (b) short-term stability at supply voltage of 300 mV. (c) Total trend of R_P, R_{AP} and ΔV_{xy} during 30-day measurements.

Supplementary Note 9. Characterization of spin Hall angle of W

We determined the SCC efficiency of channel W, *i.e.* effective spin Hall angle θ_{SH}^{eff}, utilizing spin-torque ferromagnetic resonance (ST-FMR) measurement on W (t)/CoFeB (5) heterojunction (with various thickness t in nm)[7]. **Supplementary Fig. 7a** outlines the principle of ST-FMR, *i.e.,* a microwave frequency (RF) charge current I_{RF} exerts an oscillating spin torque on CoFeB to induce magnetization precession, leading to the oscillation of the bilayer resistance due to the anisotropic magnetoresistance (AMR). When the applied in-plane field H_{ext} and the microwave Oersted field H_{RF} satisfy the ferromagnetic resonance condition, sharp peaks emerge in the measured DC voltage V_{mix}. **Supplementary Fig. 7b** illustrates the top view of a 20 μm ST-FMR device and measurement set-up. The external filed H_{ext} is applied at 45 degrees to the RF current. By separating the symmetric and antisymmetric term from the DC voltage as shown in **Supplementary Fig. 7c**, one can identify the current-induced damping-like torque (in-plane, $T_{//}$) and field-like torque (out-of-plane, $T_{\perp}$), respectively. By performing ST-FMR measurements and fitting V_{mix} versus H_{ext} across different frequency as shown in **Supplementary Fig. 7d**, θ_{SH}^{eff} can be generally determined. The inset in **Supplementary Fig. 7d** gives θ_{SH}^{eff} dependence on frequency, giving a value about -0.26, matching with previous reports for several-nanometer-thick W. Furthermore, **Supplementary Fig. 7e** illustrates W resistivity ρ and θ_{SH}^{eff} as a function of thickness t. For $t < 4$, ρ and θ_{SH}^{eff} indicate that it is in β phase, and for $t > 5$, it is α phase[8,9].

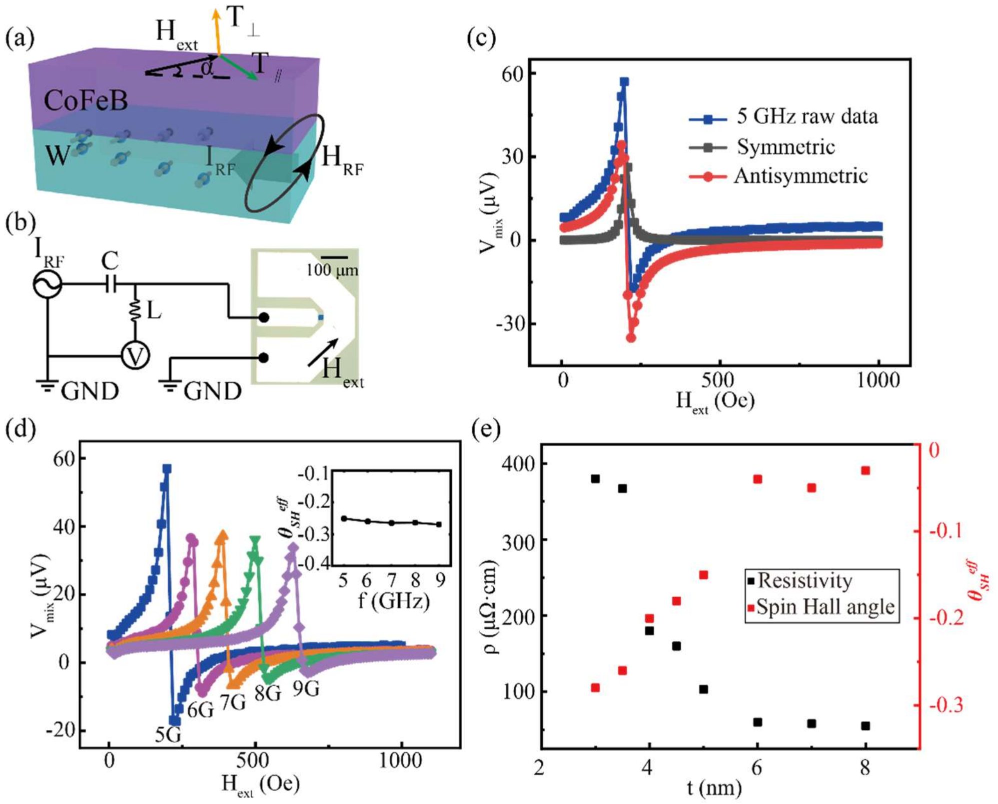

Supplementary Fig. 7. Evaluation of spin Hall angle of W channel. (a) Illustration of the ST-FMR mechanism. (b) Measurement set-up of ST-FMR. (c) ST-FMR result at 5 GHz excitation current and extractions of current-induced damping-like torque (symmetric term) and field-like torque (antisymmetric term). (d) ST-FMR results at different frequency. Inset: the deduced effective spin Hall angle. (e) Resistivity and effective spin Hall angle of W as a function of thickness.

Supplementary Note 10. Details of bilayer microdot readout device

Bilayer microdot readout device is used to determine what kind of SOC material in using and as control experiments of MTJ-enhanced readout device. We compared output signals in 2 samples: Pt(6)/CoFeB(5) and W(6)/CoFeB(5). The microdot design is shown in **Supplementary Fig. 8a**. Pt or W thickness is chosen to be 6 nm for over etching process, as signals of CoFeB, W and Pt are all hard to be detected during etching as schematically shown in **Supplementary Fig. 8b**. Device under microscope is shown in **Supplementary Fig. 8c**, presenting about 1 μm× 2.5 μm microdot with 3-μm-width channel. We found that signal with W is larger than that with Pt, and their polarities are opposite as shown in **Supplementary Fig. 8d**. The bilayer device W(6)/CoFeB(5) is also used as a control one in main manuscript, as a comparison of W/MTJ device.

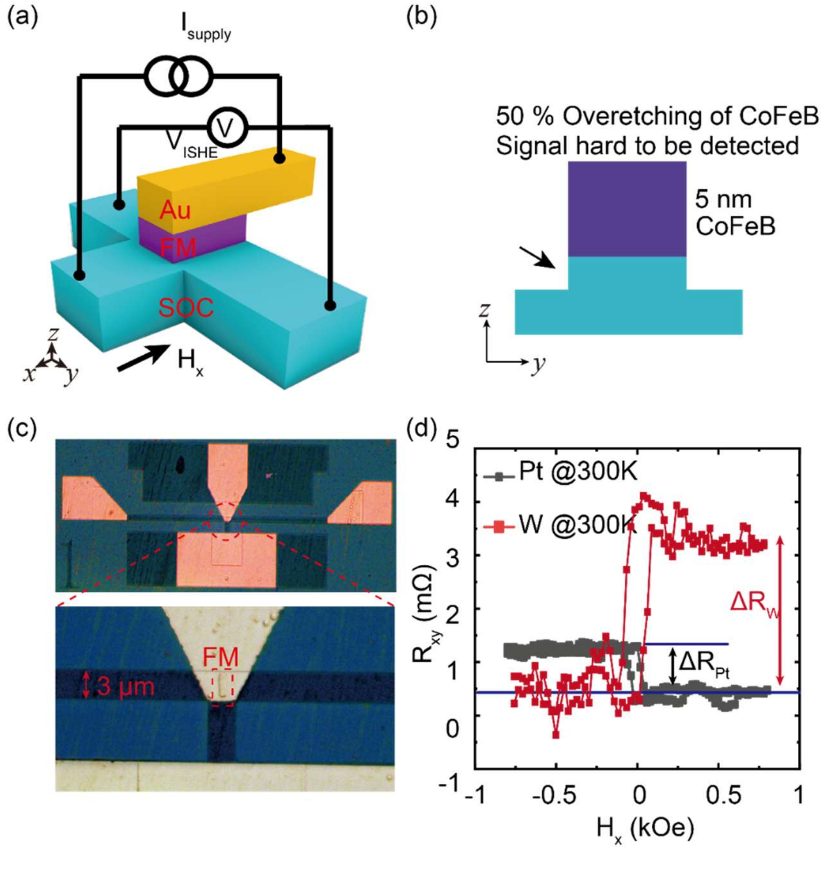

Supplementary Fig. 8. Bilayer microdot readout device. (a) Measurement set-up. (b) Device etching process. (c) Top view of microdot device. (d) Signal of Pt/CFB and W/CFB.

Supplementary Note 11. Detailed description of the numerical model

Following the existing one-dimensional spin diffusion numerical model[4] as formula (2), and concerning the physical measurements in our experiments, we give the definition of parameters and their typical values in **Supplementary Table 1**. Here, SOC and FM is short for spin-orbit coupling layer and ferromagnetic layer, respectively. These values would yield about 400 times difference between W/CFB-bilayer device and MTJ-enhanced device as shown in the main text **Fig 5b**.

$$\Delta R_{SCC} = \frac{SP\cdot\theta_{SH}\lambda_{SOC}}{(\frac{t_{FM}}{\rho_{FM}}+\frac{t_{SOC}}{\rho_{SOC}})w_{SOC}} \times \frac{1-\frac{1}{\cosh(\frac{t_{SOC}}{\lambda_{SOC}})}}{\tanh(\frac{t_{SOC}}{\lambda_{SOC}})+\frac{\lambda_{SOC}\rho_{SOC}}{\lambda_{FM}\rho_{FM}^{*}}\tanh(\frac{t_{FM}}{\lambda_{FM}})} \quad (2)$$

Supplementary Table 1| Typical values used in numerical calculations

Parameter	Definition	Value	Ref.
**SP**	Spin polarization rate	$SP_{CoFe} = 0.2$<br>$SP_{MTJ} = 0.92$	ab-initio calculation of this work
$\boldsymbol{\theta_{SH}}$	Spin Hall angle	$\theta_{SH\_\alpha W} = -0.04$<br>$\theta_{SH\_\beta W} = -0.26$	Experiment of this work
$\boldsymbol{\lambda_{SOC}}$ ($\boldsymbol{\lambda_{FM}}$)	Spin diffusion length of SOC (FM) material	$\lambda_W = 2.1$ nm<br>$\lambda_{CoFe} = 2$ nm	10
$\boldsymbol{t_{SOC}}$ ($\boldsymbol{t_{FM}}$)	Thickness of SOC (FM) material	$t_{\alpha W} = 6$ nm<br>$t_{\beta W} = 3.5$ nm<br>$t_{CoFe\_bilayer} = 5$ nm<br>$t_{CoFe\_MTJ} = 1.9$ nm	Experiment of this work
$\boldsymbol{\rho_{SOC}}$ ($\boldsymbol{\rho_{FM}}$)	Resistivity of SOC (FM) material	$\rho_{\alpha W} = 60$ μΩ·cm<br>$\rho_{\beta W} = 367$ μΩ·cm<br>$\rho_{CoFe} = 75$ μΩ·cm	Experiment of this work
$\boldsymbol{\rho_{FM}^{*}}$	Effective resistivity of FM material	$\rho_{CoFe}/(1-SP^2)$	4

Supplementary Note 12. Detailed structure of MTJ in the simulation

To investigate the difference in spin polarization between CoFeB and MTJ, we consider W/CoFe/W and W/CoFe/MgO/CoFe/W structures in the ab-initio calculations, as shown in **Fig. 5c** and **5d**. To minimize the lattice mismatch, we employ a face-centered cubic structure for W [100] deposited on the CoFe [100] surface, while the tunneling layer MgO is oriented along [110]. The in-plane lattice constants of the two structures are set to 2.83 Å, consistent with bulk CoFe. The W/CoFe/W structure contains 19 monolayers (19 ML) of CoFe between the W leads, while the scattering region of W/CoFe/MgO/CoFe/W is composed of 5 ML CoFe, 5 ML MgO, and 5 ML CoFe. After relaxation, the optimized distances are 1.64 Å between the interfacial W and Co layers, as well as 1.97 Å between MgO and Co. As a result, the scattering region lengths of the two structures are comparable, with 26.33 Å for W/CoFe/W and 26.51 Å for W/CoFe/MgO/CoFe/W.

Supplementary Note 13. Processing logic using "MTJ+MESO" unit

To investigate the prospective application of our "MTJ+MESO" unit, we designed a processing logic as shown in **Supplementary Fig. 9a**. The underlayer is composed of MESO array for low-power logic operation and the top layer is an MTJ array for memory operation. The MESO array and MTJ array is intrinsically-integrated using our proposed device and thus there is no bandwidth mismatch during computing. We show a detailed part of this design in **Supplementary Fig. 9b**, where the bottom MESO logic array forms one-bit full adder. The logic array and memory array share the same transistor for selector and power supply. Word lines (WL) and bit lines (BL) are connected to peripheral column decoder and row decoder. Here, the top MTJ array serves as magnetic random-access memory (MRAM). Through this MRAM, the data can be easily transferred to the computation logic of MESO array while the calculation results can be read out to external units. Moreover, the naturally integrated MTJ and MESO enables instantaneous and nonvolatile data exchange between computing module and external memory. In addition, this architecture allows for the extraction of any intermediate process results when required, which significantly diminishes the reliance on registers in specific computational programs. Moreover, the memory array can be utilized as error correction by monitoring its resistance state during computing. On this basis, more novel operation modes and architectures can be explored.

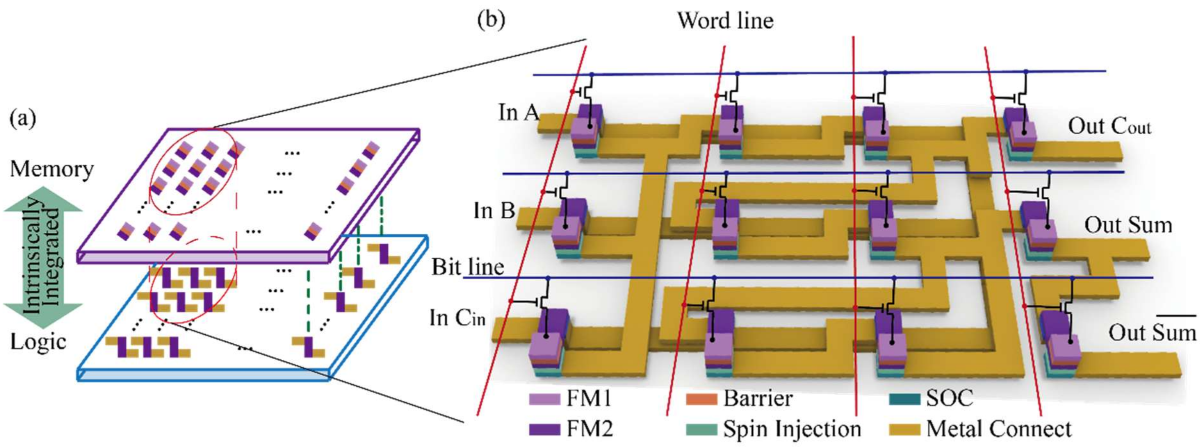

Supplementary Fig. 9. Processing unit using MTJ-enhanced MESO logic. (a) Intrinsically-integrated logic array and memory array with vertical distribution. (b) Detailed view of a one-bit full adder logic and memory unit. FM1 in light purple and FM2 in deep purple represent two ferromagnetic layers. A and B are two one-bit operands, C_{in} is input carry bit, Sum is sum for A, B and C_{in}, and C_{out} is output carry bit.

References

1. Polley, A., Ramaswamy, S. M. & Haroun, B. S. Residual offset in silicon Hall-effect sensor: Analytical formula, stress effects, and implications for octagonal Hall plate geometry. *IEEE Sens. J.* **20**, 11283 (2020).
2. Choi, W. Y. et al. All-electrical spin-to-charge conversion in sputtered Bi_xSe_{1-x}. *Nano. Lett.* **22,** 7992 (2022).
3. Groen, I., Pham, V. T., Leo, N., Marty, A., Hueso, L. E. & Casanova, F. Disentangling spin, anomalous, and planar Hall effects in ferromagnet–heavy-metal nanostructures. *Phys. Rev. Appl.* **15**, 044010 (2021).
4. Pham, V. T. et al. Spin–orbit magnetic state readout in scaled ferromagnetic/heavy metal nanostructures. *Nat. Electron.* **3**, 309 (2020).
5. Kuroiwa, T., Takenaga, T., Sadeh, B., Kobayashi, H. & Sato, K. Read-cycle endurance of magnetic random access memory elements. *IEEE Trans. Magn.* **40,** 2631 (2004).
6. Xiang, X. H., Zhu, T., Du, J., Landry, G. & Xiao, J. Q. Effects of density of states on bias dependence in magnetic tunnel junctions. *Phys. Rev. B* **66,** 174407 (2002).
7. Liu, L., Moriyama, T., Ralph, D. C. & Buhrman, R. A. Spin-torque ferromagnetic resonance induced by the spin Hall effect. *Phys. Rev. Lett.* **106,** 36601 (2011).
8. Sriram, K., Mondal, R., Pradhan, J., Haldar, A. & Murapaka, C. Structural phase engineering of (α+β)-W for a large spin Hall angle and spin diffusion length. *J. Phys. Chem. C* **127,** 22704 (2023).
9. Demasius, K.-U. et al. Enhanced spin-orbit torques by oxygen incorporation in tungsten films. *Nat. Commun.* **7,** 10644 (2016).
10. Wang, H. L., Du, C. H., Pu, Y., Adur, R., Hammel, P. C. & Yang, F. Y. Scaling of spin Hall angle in 3d, 4d, and 5d metals from $Y_3Fe_5O_{12}$/metal spin pumping. *Phys. Rev. Lett.* **112,** 197201 (2014).
