# Supplementary material for: Giant spin-orbit magnetic state readout enhanced by a magnetic tunnel junction
Source: Nat Commun. 2026 May 26;17:6838. doi: 10.1038/s41467-026-73382-9 (PMC13388924; doi:10.1038/s41467-026-73382-9)
Supplement: Supplementary file 2 — Transparent Peer Review file [file 41467_2026_73382_MOESM2_ESM.pdf]

nature portfolio

# Peer Review File

## Giant Spin-Orbit Magnetic State Readout Enhanced by a Magnetic Tunnel Junction

Corresponding Author: Professor Weisheng Zhao

**This file contains all reviewer reports in order by version, followed by all author rebuttals in order by version.**

Version 0:

Reviewer comments:

Reviewer #1

(Remarks to the Author)
This paper presents an MTJ-enhanced magnetoelectric spin-orbit (MESO) logic that addresses the inherently weak readout voltage of conventional MESO devices. By integrating a CoFeB/MgO/CoFeB magnetic tunnel junction with the MESO structure, the authors leverage tunneling-magnetoresistance modulation and spin-filtering effects to boost the spin-to-charge conversion efficiency. The proposed scheme achieves a record 1.5 mV readout voltage, about two orders of magnitude higher than prior reports, while enabling nonvolatile in-situ data exchange between logic and memory. Although this approach significantly strengthens output signal and scalability toward practical MESO logic, several issues need to be addressed before publication.

1. The explanation of how the proposed MTJ-enhanced MESO logic operates is insufficient. Detailed explanations should be added regarding: 1) How Vout is output when Vin is input, 2) The mechanism enabling switching of the MTJ free layer, 3) The mechanism generating the Vout output, and 4) The mechanism by which TMR increases the difference in Vout.

2. For cascading logic, the voltages of Vin and Vout are required, and shouldn't Vout be connected to the Vin of the next MESO logic? In this case, shouldn't the analysis proceed using Vout rather than delta Vout (Vxy, V2)?

3. In Fig. 1, Vout is connected to GND. How can the Vout voltage and delta Vout (Vxy) occur as shown in equation (1)? From the circuit design perspective, it seems Vout should remain connected to GND.

4. The definition of transverse output voltage is unclear. In page 4 line 103-104, transverse outout voltage is mentioned to be the voltage between lead 2 and 3. However, in Fig. 2(d), the transverse voltage V2 (Vxy) is the voltage between lead 2 and 4.

Reviewer #2

(Remarks to the Author)
Recommendation: This paper is probably publishable, but major revision is needed;
Comments:
In this work, the authors proposed an integrated design in which "the MTJ shares a ferromagnetic layer with the MESO structure," successfully boosting the output voltage of the readout module to 1.5 mV—an improvement of two orders of magnitude over conventional schemes. This effectively addresses the key bottleneck in the cascading of MESO logic, underscoring the clear application potential of this research direction. Therefore, it is recommended for possible publication in this journal after some mandatory revisions based on the following comments.

1.The authors claim to be the "first to propose an MTJ-enhanced MESO logic," yet they fail to clearly differentiate their work from previous studies on the integration of MTJs with spintronic devices—such as whether a shared magnetic layer is adopted or whether the design specifically targets the MESO cascading challenge. It would be valuable to include a comparative analysis with one or two representative prior works to better articulate the originality of the proposed integration architecture.
2.Regarding the characterization of MgO crystallinity, the authors mention that "the MgO barrier is well-crystallized," but this claim is only indirectly supported by the cross-sectional view in Fig. 2e. Direct characterization data is lacking.

3.It is recommended to include short-term stability test results or a discussion on future reliability study plans. This would significantly enhance the persuasiveness of the work from an application-oriented perspective.
4.In Figure 3, the spin-to-charge conversion signal exhibits asymmetry under positive and negative biases, which the authors attribute to the bias-dependent spin polarization rate. Are there more direct simulation data to support this explanation?
5.It is suggested that more details of device fabrication and simulation (such as the Hubbard U value) be provided in the Methods section.
6.The authors mention that the MTJ enables "instantaneous data exchange", but have not tested the timing characteristics of magnetic state switching. The lack of direct data undermines the rigor of the work.
7.In Fig. 2b, “the RA value increases with increasing MgO thickness, while the TMR ratio exhibits an initial increase followed by a decrease”.As the thickness of the insulating layer increases, scattering intensifies, and the TMR should keep rising. Why does the TMR first increase and then decrease? What are the implications of the increased TMR (Tunneling Magnetoresistance) ratio and spin polarization in the MTJ structures for the efficiency of the MESO logic system?
8.In Fig.5c and 5d, the data of the analog devices are overly simplistic, lacking a large number of parameters. There is even no information on the lattice matching of the scattering region and the electrode region, as well as the spacing between various parts after device optimization, etc. Good work should be reproducible, and the author needs to complete all the data supplementation.
9.The subheading of Part 2.3 is: Mechanism Explorations. However, only the matching states of the wave functions of the majority electrons and the minority electrons in the Brillouin zone are described.This part merely describes the phenomenon and does not provide sufficient explanation of the physical mechanism. The author should improve this aspect.

Reviewer #3

(Remarks to the Author)
In this manuscript, the authors present the integration of MESO and MTJ devices, achieving an output voltage of approximately 1.5 mV. This result indicates a measurable improvement in readout performance and represents a step toward the practical implementation of MESO technology. However, the evidence supporting the claimed “robust ISHE readout” remains limited, and additional quantitative analysis would strengthen the manuscript. With further clarification and experimental validation, the work could make a meaningful contribution to the field.

1. From Eq. (1) derived by the author in the main text, Vsupply / RMTJ, AP is equal to IAP, and IAP seems dominant over the ΔVxy in the MTJ device with a voltage supply source. How was the current amplitude under the AP state for the voltage source in Fig. 4a and 4b? The ΔRxy in Fig. 4b is discrete in positive supply voltage, while flat in the negative supply voltage. Consider the variation of SP rate may not be enough to account for the large changes in the output signal, as shown in ref. 36. Does it originate from the voltage dependence of current?

2. The output signal of the MTJ-enhanced device at Isupply = 10μA in Fig. 5a was quite smaller than the value of the EBL device using current sources in Fig. 3. Also, the squareness of the hysteresis loop was decreased in the device depicted in Fig. 5. What is the dimension of the microdot device?

3. The substantially increased output signal originating from the MTJ modulation was investigated. However, in the mechanism explorations, the author claimed the difference between the MTJ sample and the control sample was due to the spin polarization(SP), resistivity, and spin Hall angle(θSH). Given two conflicting statements, please explain which one is the main contributor, and how do you quantify it?

4. The authors should clarify their rationale for selecting W (6 nm) as the control sample instead of W (3.5 nm). Given that increasing the W thickness typically enhances electrical conductivity while reducing the spin-to-charge conversion efficiency. Whether the robust ISHE readout can be maintained when replacing W (3.5 nm) with W (6 nm) in the MTJ-enhanced device?

5. Obviously, the top pinned CoFeB will exert the spin transfer torque of the bottom free layer. Will the supply current switch the magnetization of the bottom CoFeB in the absence of an external field? Is it the reason why the measurements of the current source are only below 40μA?

6. Correct the typos in the manuscript. For example, On Page 7, line 202, the second term in square brackets did not match the Eq. 1 derived in the supplementary materials on Page 4, line 77. Similarly, on Page 15, line 358, correct the unit in Figure 4a inset.

7. The format of the references should keep the same style.

Version 1:

Reviewer comments:

Reviewer #1

(Remarks to the Author)
1. The authors describe the proposed MTJ-enhanced MESO write process as follows: “An input voltage Vin charges the

capacitor of multiferroic material (ME layer, depicted in dark blue), switching the ferroelectric polarization." However, it is unclear how Vin can switch the ferroelectric polarization. The ferroelectric polarization must be able to change depending on Vin, yet Vin is always smaller than Vsupply and larger than GND, while the top terminal of the ME layer is always connected to Vsupply. Therefore, a clear explanation is needed on how the ferroelectric polarization can change depending on Vin (when Vin increases, when Vin decreases).

2. In Figure R1, Vout appears to be simply connected to GND. Figure R3 includes Rsoc,v, but this resistance is expected to be much smaller than RMTJ. In this case, Vout is interpreted similarly to GND. Furthermore, Figure R1(d) states that Vout can be either a positive or negative voltage depending on Vin. The authors also mention that Vout is -ΔVxy/2 or ΔVxy/2. However, since the MTJ (FM layer, SI layer, SOC layer) is connected to Vsupply and GND, Vout cannot be a negative voltage. A clear explanation of this point is considered necessary.

Reviewer #2

(Remarks to the Author)
The authors have addressed all my comments point-by-point in their response, and I am satisfied with their revisions. I therefore recommend the manuscript for acceptance by the journal.

Reviewer #3

(Remarks to the Author)
I would like to thank the authors for their detailed responses. I am pleased to see that the concerns regarding the readout mechanism have been well-addressed. The integration of a Magnetic Tunnel Junction (MTJ) with the spin-orbit (SO) component is indeed a brilliant idea, and the rebuttal clearly demonstrates how this enhances the readout voltage effectively.

However, I still have a significant reservation regarding the magnetoelectric (ME) component of the proposed system. While the readout module is now well-defined, the authors have not sufficiently clarified what specific ME material system is claimed to support this MTJ-based MESO logic.

Specifically, do the authors intend to stick with $BiFeO_3$ (BFO) as the multiferroic element? If BFO is indeed the material of choice, I must point out that the Pulsed Laser Deposition (PLD) process typically required for high-quality BFO is unfriendly to standard CMOS processes. This raises a critical question about the practical scalability of the device: How do the authors envision making such a MESO structure commercially available and compatible with CMOS-acceptable processes?

While the enhanced readout voltage is a major step forward, the feasibility of the ME writing stage is equally critical for the device's viability. I would appreciate a more detailed discussion on the material selection for the ME layer and a proposed roadmap for its integration using industry-standard fabrication techniques.

Version 2:

Reviewer comments:

Reviewer #1

(Remarks to the Author)
Thank you for providing such a sincere and detailed response to my challenging question from a circuit operation perspective. Most of my doubts have been resolved through your answers. However, I disagree with one part of the authors' response, so I am asking again. The authors answered as follows:
"Vout can be negative, which is promised by the SOC mechanism."
"Vout is an electric force generated by a physical mechanism, not a voltage dependent on voltage division."
However, the FM-SI-SOC is connected to Vsupply and GND. Considering the operation within the SOC layer, electrons flow from GND to Vsupply (opposite to the current flow). Yet, electrons aligned with a specific spin due to the spin-Hall effect, centered on node A, can be concentrated and flow toward one side of the SOC layer. Consequently, I believe the Vout can increase or decrease relative to the A node voltage depending on the FM spin direction. However, from this operational perspective, I do not think the SOC mechanism can generate a voltage higher than Vsupply or lower than GND. This is because current flows through the SOC layer in a voltage division configuration.
As the author's response suggests, if the SOC mechanism can generate a voltage lower than GND (similar to a charge pump in a circuit), further explanation would be necessary.

Reviewer #3

(Remarks to the Author)
The authors have satisfactorily addressed the concerns raised in my previous review. The revisions have improved the clarity and rigor of the manuscript, and I find the current version to be strong and suitable for publication. I therefore recommend acceptance in Nature Communications.

Version 3:

Reviewer comments:

Reviewer #1

(Remarks to the Author)
Thank you. I have no further questions regarding this manuscript.

**Open Access** This Peer Review File is licensed under a Creative Commons Attribution 4.0 International License, which permits use, sharing, adaptation, distribution and reproduction in any medium or format, as long as you give appropriate credit to the original author(s) and the source, provide a link to the Creative Commons license, and indicate if changes were made.
In cases where reviewers are anonymous, credit should be given to 'Anonymous Referee' and the source.
The images or other third party material in this Peer Review File are included in the article's Creative Commons license, unless indicated otherwise in a credit line to the material. If material is not included in the article's Creative Commons license and your intended use is not permitted by statutory regulation or exceeds the permitted use, you will need to obtain permission directly from the copyright holder.
To view a copy of this license, visit https://creativecommons.org/licenses/by/4.0/

**Response Letter**

Dear Editor of Nature Communications,

Thank you very much for considering our manuscript entitled "Giant Spin-Orbit Magnetic State Readout Enhanced by a Magnetic Tunnel Junction" (ID: NCOMMS-25-83756A). We are very grateful to the reviewers for taking the time to review our manuscript and providing very helpful review comments, which allowed us to improve the clarity and quality of this article. We have considered in detail the questions and comments of all the reviewers and prepared a revised version of our manuscript. Please find below our point-by-point response to the review comments (indicated in blue color). We have also highlighted the changes made in the main manuscript in blue color so that they can be located easily.

We are looking forward to hearing your favorable response.

Yours sincerely,

Prof. Weisheng Zhao, on behalf of the authors

National Key Laboratory of Spintronics, Hangzhou International Innovation Institute, Beihang University, Hangzhou, China

Email: weisheng.zhao@buaa.edu.cn

**Comments to Authors:**

***Reviewer #1***

***Comment #***

*Reviewer wrote*: This paper presents an MTJ-enhanced magnetoelectric spin-orbit (MESO) logic that addresses the inherently weak readout voltage of conventional MESO devices. By integrating a CoFeB/MgO/CoFeB magnetic tunnel junction with the MESO structure, the authors leverage tunneling-magnetoresistance modulation and spin-filtering effects to boost the spin-to-charge conversion efficiency. The proposed scheme achieves a record 1.5 mV readout voltage, about two orders of magnitude higher than prior reports, while enabling nonvolatile in-situ data exchange between logic and memory. Although this approach significantly strengthens output signal and scalability toward practical MESO logic, several issues need to be addressed before publication.

*Our Response*:

Thank you very much for the recognition of our work as well as your constructive suggestions and comments which helped us improve the quality of this manuscript.

*Reviewer wrote*: *(Question 1)* The explanation of how the proposed MTJ-enhanced MESO logic operates is insufficient. Detailed explanations should be added regarding: 1) How $V_{out}$ is output when $V_{in}$ is input, 2) The mechanism enabling switching of the MTJ free layer, 3) The mechanism generating the $V_{out}$ output, and 4) The mechanism by which TMR increases the difference in $V_{out}$.

Our Response:

We greatly appreciate your constructive suggestions. Operation process is a very important to understand the proposed MTJ-enhance MESO logic, which is not clearly discussed in the first version of manuscript. We explain these concerns point by point as below:

1) How $V_{out}$ is output when $V_{in}$ is input

We clarify this by using a conventional MESO logic device as shown **Figure R1a**, which contains the same writing and reading process as proposed MTJ-enhanced

MESO. MTJ in proposed device only enhances $V_{out}$ but plays no role in the transfer between $V_{in}$ and $V_{out}$.

The writing process happens between the magnetoelectric (ME) layer and ferromagnetic (FM) layer, based on ME effect and convert input charge signal to spin signal, as shown **Figure R1b**. An input voltage $V_{in}$ charges the capacitor of multiferroic material (ME layer, depicted in dark blue), switching the ferroelectric polarization. Due to ME effect, the (anti)ferromagnetism of ME layer changes, and interface exchange bias between ME layer and FM layer reverses, which can further reverse the magnetization of FM layer.

The reading process happens between the FM layer, spin-injection (SI) layer and spin-orbit coupling (SOC) layer, as shown **Figure R1c**. The control signal $V_{supply}$ injects charge current, which is polarized to spin current by FM and SI layers. The spin current is then injected to SOC layer and converted to charge flow, yielding an output voltage $V_{out}$. When the polarity of $V_{in}$ is inverted, FM order reverses, and thus the output $V_{out}$ reverses.

Compared with CMOS transistors, $V_{in}$ in MESO can be regarded as drain voltage, $V_{out}$ as source voltage, $V_{supply}$ as gate voltage. $V_{supply}$ controls transfer between Vin and $V_{out}$. **Figure R1d** shows the ideal transfer between $V_{in}$ and $V_{out}$.

2) The mechanism enabling switching of the MTJ free layer

The free layer switching of MTJ-enhanced MESO is also based on ME effect, as discussed above. MTJ in proposed device acts as an amplifier in reading process and only enhances $V_{out}$, but plays no role in writing process. This switching process is not experimentally demonstrated in this work.

We proposed the concept of MTJ-enhanced MESO logic and demonstrated the MTJ-enhanced readout signal. In our measurements, the free layer magnetization switching is realized by external field, which is helpful and convenient for preliminary concept demonstration.

3) The mechanism generating the $V_{out}$ output

The $V_{out}$ is generated by spin-to-charge conversion (SCC). Theoretically, this signal is originated from inverse spin Hall effects (ISHE) as shown in **Figure R1c**,

which converts injection spins into a transverse electric voltage through asymmetric scattering driven by strong SOC. As a spin current flows, the spin-orbit interaction deflects electrons toward transverse direction, resulting in a measurable charge accumulation.

In our experiments, the measurement results could contain multiple Hall effects. These disturbs by various Hall effects also reflect the magnetic state of free layer, and can be excluded in our measurements, as discussed in **Supplementary Material S4**.

4) The mechanism by which TMR increases the difference in $V_{out}$.

In the proposed MTJ-enhanced MESO logic device as shown in **Figure R1e**, MTJ is integrated in the reading module of MESO, which can amplify the output voltage by TMR-related current modulation and spin filtering effects.

As shown in **Figure R1f**, the injection tunneling current experiences a sudden increase upon the transition of MTJ from antiparallel (AP) to parallel (P) state under a constant voltage supply $V_{supply}$, *i.e.*, from $I_{AP}$ to $I_P$. This current alteration yields a spin injection difference, which can induce an extra voltage signal superposed to the SCC output signal, and amplify the output signal of MESO. As shown in **Figure R1g**, Δ1 band electrons in sandwiched CoFeB/MgO/CoFeB tunnel easily when the magnetization of two CoFeB layers are parallel alignment, and hard when they are antiparallel, while Δ2 and Δ5 band electrons count little. Consequently, tunneling current across an MTJ could be highly polarized to significantly enhance SP rate. In the proposed device, the ideal transfer functions between $V_{in}$ and TMR, $V_{in}$ and $V_{out}$, are shown in **Figure R1h**. An input voltage $V_{in}$ could switch magnetization, which can be detected by TMR, and the output voltage $V_{out}$ reverses with reversing $V_{in}$.

To quantify the $V_{out}$ under current and voltage source and showing the TMR influence directly, we derive $\Delta V_{xy}$ under different source as below:

Under a current source,

$$\Delta V_{xy} = V_{xy}^{P} - V_{xy}^{AP} = I_{supply}R_{xy}^{P} - I_{supply}R_{xy}^{AP} = I_{supply}\Delta R_{xy} \quad \text{(R1)}$$

According to **Supplementary Material S11**,

$$\Delta R_{xy} = 2\Delta R_{SCC} \propto \frac{2SP\cdot\theta_{SH}}{w_{SOC}} \quad \text{(R2)}$$

Where SP is spin polarization rate, $\theta_{SH}$ is spin Hall angle and $w_{SOC}$ is width of SOC material.

As a result, in current source condition, SP increases with TMR, leading to an enhancement of output signal $\Delta R_{xy}$ and $\Delta V_{xy}$, corresponding to spin filtering effect.

Under a voltage source

When MTJ is switched from AP state to P state, current decreases

$$\Delta I = I_P - I_{AP} = V_{supply}\left(\frac{1}{R_{MTJ}^{P}} - \frac{1}{R_{MTJ}^{AP}}\right) = TMR \cdot I_{AP} \quad \text{(R3)}$$

$V_{out}$ changes

$$\Delta V_{xy} = V_{xy}^{P} - V_{xy}^{AP} = I_P R_{xy}^{P} - I_{AP} R_{xy}^{AP} = I_P R_{xy}^{P} - (I_P - \Delta I) R_{xy}^{AP} = (I_{AP} + \Delta I) R_{xy}^{P} - I_{AP} R_{xy}^{AP} \quad \text{(R4)}$$

Assuming that in this condition, $R_{xy}^{P} - R_{xy}^{AP}$ is still equal to $\Delta R_{xy}$ (this is reasonable that $R_{xy}$ changes little when current is large enough as shown in **main text Figure 3d**), then

$$\Delta V_{xy} = I_P \Delta R_{xy}^{at\, I_P} + TMR \cdot I_{AP} R_{xy}^{AP} = I_{AP} \Delta R_{xy}^{at\, I_{AP}} + TMR \cdot I_{AP} R_{xy}^{P} \quad \text{(R5)}$$

Compared equation (R5) with equation (R1), TMR brings another term in output voltage signal (corresponding to current modulation effect).

In conclusion, the proposed MTJ-enhanced MESO logic shares similar working process as conventional MESO. That is, charge to spin conversion in writing process and spin to charge conversion in reading process. MTJ functions only in reading process to amplify output signal.

***Corresponding change in manuscript:*** *Yes*

*Location of Change: Part 1, Paragraph 3, Page #2*

To make clear the MTJ-enhance MESO logic operation process, the below expression has been added in the revised main text: ‘The proposed device characterizes the same writing process with conventional MESO logic, *i.e.*, $V_{in}$ charges the capacitor of multiferroic material (depicted in dark blue), switching in sequence the ferroelectric polarization, interface exchange bias between ME layer and FM layer, and

magnetization of FM layer. MTJ in proposed device acts as an amplifier in reading process, where two different magnetic state of free layer is converted into separated transverse charge signal $V_{out}$.'

*Location of Change: Supplementary Material S6*

The below expression has been added at last of **Supplementary Material S6**: 'Assuming that $\Delta R_{xy} = R_{xy}^{P} - R_{xy}^{AP}$, thus $\Delta V_{xy} = I_{AP}\Delta R_{xy} + TMR \cdot I_{AP}R_{xy}^{P}$. The first term is equal to the signal under a current source of $I_{AP}$, and the second term is an extra signal by MTJ current modulation.'

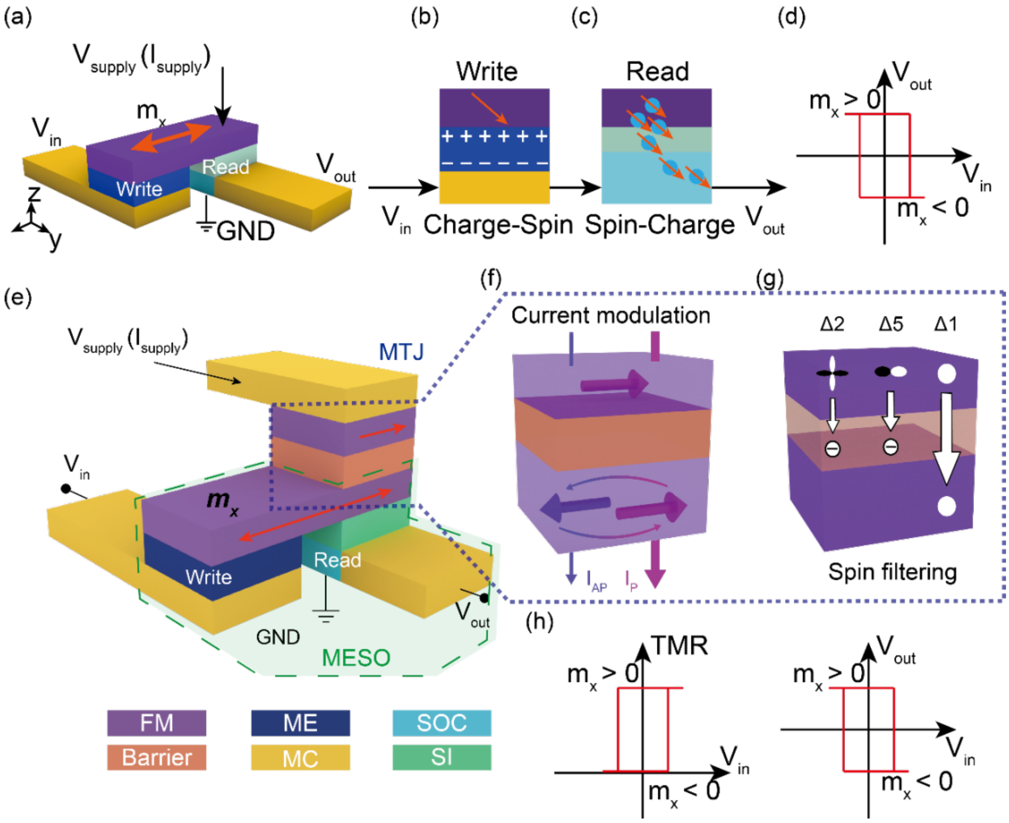


Figure R1. Operation process of MESO and MTJ-enhanced MESO. (a) Schematic of conventional MESO logic device. (b) Writing process of MESO. (c) Reading process of MESO. (d) Ideal transfer function between $V_{in}$ and $V_{out}$ (e) Schematic of proposed MTJ-enhanced MESO logic device. (f) Current modulation by MTJ resistance. (g) Spin filtering by Δ1-band-dominated spin tunneling. (h) Ideal transfer functions between $V_{in}$ and TMR, $V_{in}$ and $V_{out}$, respectively.

*Reviewer wrote*: (*Question 2)* For cascading logic, the voltages of $V_{in}$ and $V_{out}$ are

required, and shouldn't $V_{out}$ be connected to the $V_{in}$ of the next MESO logic? In this case, shouldn't the analysis proceed using $V_{out}$ rather than delta $V_{out}$ ($V_{xy}$, $V_2$)?

*Our Response*:

Thank you for your comments. It is true that $V_{out}$ shall be connected to the $V_{in}$ of the next MESO logic, but $V_{out}$ is evaluated by $\Delta V_{xy}$ rather than $V_{xy}$. This is usually misunderstood. Below is the detailed description.

As we discussed in *Response for Question 1*, the output is based on spin to charge conversion, where the two different magnetic state of free layer is converted into separated transverse charge signal. The experiment set-up (**main text, Figure 2d**) is similar with Hall measurement, which could bring offset voltage $V_{offset}$ in results originating from device structural asymmetry, material/interface inhomogeneities, parasitic effects in the measurement system, and limitations of calibration methods (**IEEE Sens. J., 2020, 20, 11283**). The offset voltage is not related to magnetic state, and shall be excluded in final results.

Next, we derive the expression of $V_{offset}$ from equivalent circuit when ignoring various Hall effect. **Figure R2a** is the simplified measurement set-up, where current is injected from lead 0 to 1, and voltage is measured between lead 2 and 4. **Figure R2b** is the equivalent circuit, where $R_{ij}$ is the equivalent resistance between lead i and j. Let the conductance be $G_{ij} = 1/R_{ij}$ for each resistance. According to Kirchhoff's law, we have

$$G_{01}(V_0 - V_1) + G_{02}(V_0 - V_2) + G_{04}(V_0 - V_4) = I_{supply} \tag{R6}$$

$$G_{12}(V_2 - V_1) + G_{02}(V_2 - V_0) - G_{24}(V_2 - V_4) = 0 \tag{R7}$$

$$-G_{24}(V_4 - V_2) + G_{04}(V_4 - V_0) + G_{14}(V_4 - V_1) = 0 \tag{R8}$$

As lead 1 is connected to GND, we have $V_1 = 0$. Equations (R6) ~ (R8) can be simplified as

$$(G_{01} + G_{02} + G_{04})V_0 - G_{02}V_2 - G_{04}V_4 = I_{supply} \tag{R9}$$

$$G_{02}V_0 - (G_{02} + G_{12} + G_{24})V_2 + G_{24}V_4 = 0 \tag{R10}$$

$$G_{04}V_0 + G_{24}V_2 - (G_{04} + G_{14} + G_{24})V_4 = 0 \tag{R11}$$

Let the conductance matrix be **G**, voltage vector **V** = $[V_0\ V_2\ V_4]^T$, **I** = $[I_{suppy}\ 0\ 0]^T$. The system is **GV = I**, where

$$\mathbf{G} = \begin{bmatrix} G_{01}+G_{02}+G_{04} & -G_{02} & -G_{04} \\ G_{02} & -(G_{02}+G_{12}+G_{24}) & G_{24} \\ G_{04} & G_{24} & -(G_{04}+G_{14}+G_{24}) \end{bmatrix}$$

Using Cramer's Rule, finally we have

$$V_0 = I_{supply}[(G_{02}+G_{12}+G_{24})(G_{04}+G_{14}+G_{24}) - G_{24}^2]/\det(\mathbf{G}) \quad \text{(R12)}$$

$$V_2 = -I_{supply}[G_{02}(G_{04}+G_{14}+G_{24}) + G_{04}G_{24}]/\det(\mathbf{G}) \quad \text{(R13)}$$

$$V_4 = -I_{supply}[G_{04}(G_{02}+G_{12}+G_{24}) + G_{02}G_{24}]/\det(\mathbf{G}) \quad \text{(R14)}$$

where $\det(\mathbf{G})$ is the determinant of **G**.

Offset voltage $V_{offset}$ is given by

$$V_{offset} = V_2 - V_4 = I_{supply}[G_{04}G_{12} - G_{02}G_{14}]/\det(\mathbf{G}) \quad \text{(R15)}$$

When the device is ideal, $G_{04}$ is equal to $G_{02}$, and $G_{12}$ is equal to $G_{14}$, thus $V_{offset} = 0$, meaning there is no offset voltage. Otherwise, there exists offset voltage disturbing, and this disturb is independent of electron spins and magnetic state.

Now we take various signals into consideration. The measurement voltage $V_{xy}$ consists of spin to charge conversion voltage $V_{SCC}$ and offset voltage $V_{offset}$, *i.e.*, $V_{xy} = V_{SCC}+V_{offset}$. As we discussed in *Response for Question 1*, $V_{SCC}$ reflects the magnetic state. Thus, the difference between $V_{SCC,max}$ and $V_{SCC,min}$ (denoting two different magnetic states) is critical for cascading. As $\Delta V_{xy} = V_{xy,max} - V_{xy,min} = (V_{SCC,max}+V_{offset})-(V_{SCC,min}+V_{offset}) = V_{SCC,max} - V_{SCC,min} = \Delta V_{SCC}$, we characterize $\Delta V_{xy}$ as key parameter to evaluate the output signal.

***Corresponding change in manuscript:*** *Yes*

*Location of Change: Part 2.1, Paragraph 4, Page #4*

To avoid misunderstanding of $V_{out}$ evaluation, original expression 'The output voltage $\Delta V_{xy}$ is defined as the difference between two magnetic states' **has been modified to** 'Notably, the measurement value of $V_{xy}$ always contains offset signal originating from device structural asymmetry and other undesirable factors. $\Delta V_{xy}$, representing the difference between two magnetic states, is more reasonable to evaluate the output voltage (detailed discussion in **Supplementary Material S3**).'

*Location of Change: Supplementary Material S3*

We have added **Supplementary Material S3** to explain the offset Hall voltage.

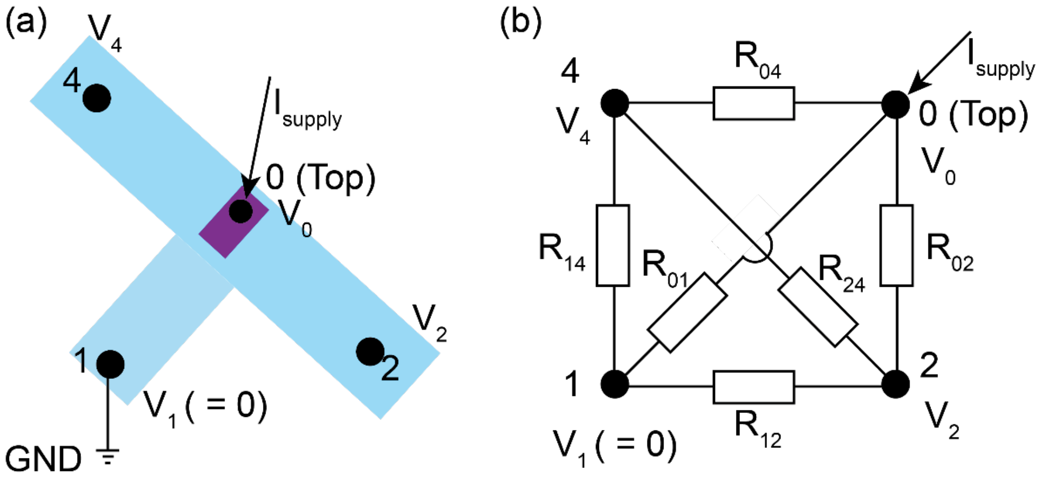


Figure R2. Measurement diagram and equivalent circuit. (a) Schematic of measurement set-up. (b) Equivalent circuit of measurement diagram.

*Reviewer wrote*: (*Question 3)* In Fig. 1, $V_{out}$ is connected to GND. How can the $V_{out}$ voltage and delta $V_{out}$ ($V_{xy}$) occur as shown in equation (1)? From the circuit design perspective, it seems $V_{out}$ should remain connected to GND.

*Our Response*:

Thank you for your constructive suggestions. We are sorry that some descriptions are confusing to electronic circuit researchers. Let us clarify again that: 1) $V_{out}$ is connected to next level $V_{in}$; 2) $V_{out}$ is evaluated by the parameter $\Delta V_{xy}$; 3) $V_{out}$ represents the two different magnetic state of free layer; 4) $V_{out}$ is separated with GND by SOC layer which functions as controlled current source with finite internal resistance; 5) For ideal device, the voltage between $V_{out}$ and GND is $-\Delta V_{xy}/2$ (*e.g.*, magnetization of FM layer along right) or $\Delta V_{xy}/2$ (*e.g.*, magnetization of FM layer along left), which is used for cascading directly.

**Figure R3** is the equivalent circuit diagram of MTJ-enhanced MESO logic device. Core components contain capacitor of ME layer $C_{ME}$, resistance of MTJ $R_{MTJ}$, controlled current source that converts vertical current $I_{supply}$ to horizontal current $I_{SOC}$. Besides, $R_{connect1}$ and $R_{connect2}$ denote connection resistance, $R_{FM}$ denotes FM layer resistance between ME and SO modules, and $R_{SOC,v}$ and $R_{SOC,h}$ denote vertical and horizontal resistance of SOC layer. For an ideal device with no asymmetry,

inhomogeneities and parasitic effects, $V_{out}$ is -$\Delta V_{xy}/2$ or $\Delta V_{xy}/2$.

***Corresponding change in manuscript:*** *Yes*

*Location of Change: Part 1, Paragraph 3, Page #3*

The follow sentences have been added in the revised main text: 'In addition, we also simplified the MTJ-enhanced MESO logic device into an equivalent circuit to analyze the its operation in the cascaded structure (**Supplementary Material S1**)'.

*Location of Change: Supplementary Material S1*

We have added **Supplementary Material S1** to explain the equivalent circuit diagram of MTJ-enhanced MESO.

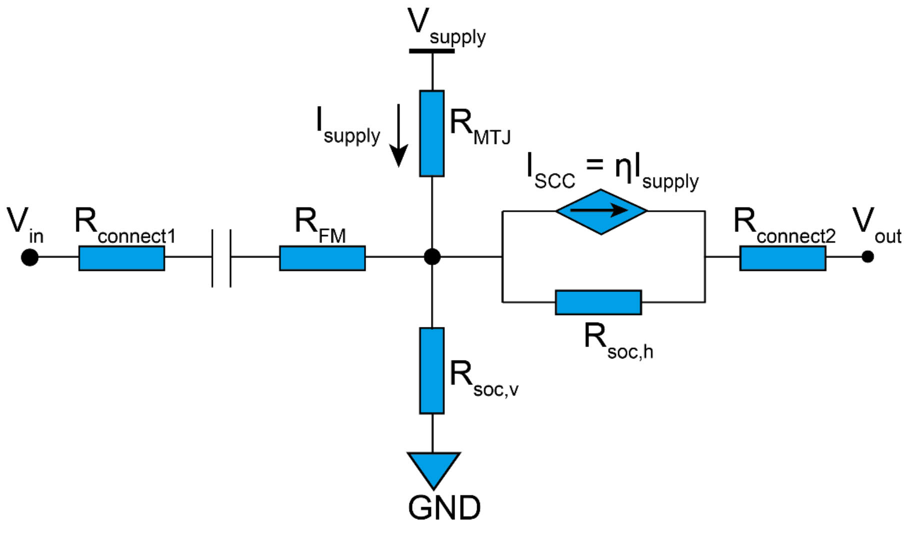


Figure R3. Equivalent circuit diagram of MTJ-enhanced MESO logic device.

*Reviewer wrote*: (*Question 4)* The definition of transverse output voltage is unclear. In page 4 line 103-104, transverse output voltage is mentioned to be the voltage between lead 2 and 3. However, in Fig. 2(d), the transverse voltage $V_2$ ($V_{xy}$) is the voltage between lead 2 and 4.

*Our Response*:

Thank you for your careful comments. This is a typographical error in the main text. Transverse output voltage is measured between lead 2 and 4. The diagram in Fig. 2(d) is correct.

***Corresponding change in manuscript:*** *Yes*

*Location of Change: Part 2.1, Paragraph 3, Page #4*

We have modified the expression ‘A cross-shape bottom electrode is used to generate transverse output voltage which can be measured between lead 2 and 3’ to ‘A cross-shape bottom electrode is used to generate transverse output voltage which can be measured between lead 2 and 4’

We are very grateful for your constructive comments and acknowledgement on this work. Your suggestions helped us improve the clarity and quality of this work. We sincerely hope that the point-by-point response will effectively address your concerns and that the revised manuscript will fully meet your requirements. Thank you again for your patience and detailed comments.

***Reviewer #2***

***Comment #***

*Reviewer wrote*: Recommendation: This paper is probably publishable, but major revision is needed;

In this work, the authors proposed an integrated design in which "the MTJ shares a ferromagnetic layer with the MESO structure," successfully boosting the output voltage of the readout module to 1.5 mV—an improvement of two orders of magnitude over conventional schemes. This effectively addresses the key bottleneck in the cascading of MESO logic, underscoring the clear application potential of this research direction. Therefore, it is recommended for possible publication in this journal after some mandatory revisions based on the following comments.

*Our Response:*

We greatly appreciate your acknowledgement of this work. Thank you for giving us the opportunity to address your concerns and further refine this paper.

*Reviewer wrote*: (*Question 1)* The authors claim to be the "first to propose an MTJ-enhanced MESO logic," yet they fail to clearly differentiate their work from previous studies on the integration of MTJs with spintronic devices—such as whether a shared magnetic layer is adopted or whether the design specifically targets the MESO cascading challenge. It would be valuable to include a comparative analysis with one or two representative prior works to better articulate the originality of the proposed integration architecture.

*Our Response:*

Thank you for your visionary comment. It is valuable to compare our proposal with similar reports about spin logic to emphasize its advantages. According to the method of data transfer between different bits, the schemes of integration MTJs with spintronic devices include all spin logic, domain-wall (DW) -based logic and our MTJ-enhanced MESO logic. In the following, we would carefully discuss these schemes to better articulate the originality of the proposed integration architecture.

First of all, we discuss all spin logic proposed by Behin-Aein B et al., in which the

spin is injected and detected by a FM layer or an MTJ, and transport along a spin-coherent channel (**Nat. Nanotechnol., 2010, 5, 266**). The core design utilizes nanomagnets to store binary information via their bistable magnetization states, and spin-coherent channels to transmit information through spin currents. By leveraging spin-torques, the device achieves information transfer between input and output magnets, with switching energy supplied by an independent voltage source.

Moreover, DW-based spin logic device, using MTJ as input and output detector is another promising spin logic. E. Raymenants et al. reported nanoscale domain wall devices with MTJ read and write (**Nat. Electron., 2021, 4, 392; IEEE IEDM, 2021, 32.3.1**). The input terminal MTJ can be written by spin transfer torque (STT), and inject DW to FM track. DW is then driven by spin orbit torque (SOT), and detected by output terminal MTJ. They also used antiferromagnetic coupled Pt/Co/Ru/Co as DW track to realize high speed DW motion. Besides, spin logic based on magnetic skyrmions, a special class of nanoscale, topologically protected domain structures, shares the similar operation with DW-based spin logic. However, skyrmions is hard to form and modulate, which poses a barrier to its experimental realization.

Then, we would like to discuss the working mode of our proposed MTJ-enhanced MESO device. The writing process of a typical MESO logic happens between the ME layer and FM layer, based on ME effect and convert input charge signal to spin signal. Meanwhile, its readout structure is consisted of FM layer, SI layer and SOC layer. A spin current from FM layer is converted into charge current in the SOC layer. Output signal is generally positively correlated with spin Hall angle $\theta_{SH}$, spin polarization SP, spin diffusion length $\lambda_{soc}$ and positively correlated with device width $w_{soc}$. Until now, various SOC materials and device dimensions have been adapted to realize large output voltage $V_{out}$. For example, Yu-Lon *et al.* experimentally demonstrated that W-based device outputs about 3 times larger than Pt-based device, and device with 95 nm width yields 4 times larger signal than that with 400 nm width (**ACS Appl. Electron. Mater., 2024, 6, 6657**). The maximum voltage they measured is around 2 μV. Topological insulators and two-dimensional electron gases could enlarge the output signal theoretically due to their high spin-to-charge conversion efficiency, but experimental

results at room temperature, to date, have not met the anticipated expectations (**Adv. Func. Mater., 2024, 34, 2307474; Adv. Func. Mater., 2023, 33, 02303878**), which greatly impends the development of MESO logic. Targeting the MESO cascading challenge, we proposed the MTJ-enhanced MESO logic where a MgO-barrier MTJ is integrated with the MESO device. The current modulation and the spin filtering effect of MgO-based MTJ can remarkably enhance the output signal to facilitate direct cascading of multiple devices. Moreover, the naturally integrated MTJ enables instantaneous and nonvolatile data exchange between computing module and external unit.

At last, we would compare the differences of these schemes. It's worth noting that all these schemes can realize data transfer in a matrix through the spin current, magnetic domain and spin-charge conversion. However, for the all spin logic and DW-based spin logic, a large writing current is required to reversal the magnetization or drive domain-wall motion, and the working voltage is about 0.5 V. For a MESO logic, 0.1 V capacitive charging voltage is enough to reversal the FM magnetization through ME effect, meaning MESO logic is more energy efficient. From this point, only MESO logic is a voltage-controlled device that can replace traditional CMOS.

***Corresponding change in manuscript:*** *Yes*

*Location of Change: Part 1, Paragraph 3, Page #3*

The below expression has been added in the revised main text: 'Compared with other MTJ-integrated spin logic devices (*e.g.*, all spin logic and domain-wall-based logic) which utilize spin torques to control input and flowing charges to transmit information, MTJ-enhanced MESO logic is more energy-efficient thanks to ultralow voltage operation[26-28]' and references 26-28 have been added.

*Reviewer wrote*: (*Question 2)* Regarding the characterization of MgO crystallinity, the authors mention that "the MgO barrier is well-crystallized," but this claim is only indirectly supported by the cross-sectional view in Fig. 2e. Direct characterization data is lacking.

*Our Response:*

Thank you for your insightful comment. The crystalline quality of MgO is crucial for spin filtering effect, and the initial demonstration by TMR measurement and large-scale transmission electron microscope (TEM) image is not very convincing.

To address this concern and further figure out directly the characterization of MgO crystallinity, we have supplemented high-resolution TEM (HRTEM) characterization (presented as **Figure R4**). This image clearly resolves the continuous lattice fringes of MgO barrier layer within the MTJ stack. Additionally, interfaces between different layers are well-defined.

In conclusion, both TMR measurement and HRTEM characterization confirms that MgO barrier is well-crystallized and could functions for spin filtering.

***Corresponding change in manuscript:*** *Yes*

*Location of Change: Part 2.1, Paragraph 4, Page #4*

The following sentence has been added in *Part 2.1* 'High-resolution transmission electron microscope image of MTJ stacks also resolves the continuous lattice fringes of MgO barrier, indicating good crystallization (**Supplementary Material S2**).'

*Location of Change: Supplementary Material S2*

We have added **Supplementary Material S2** to present MgO crystallinity.

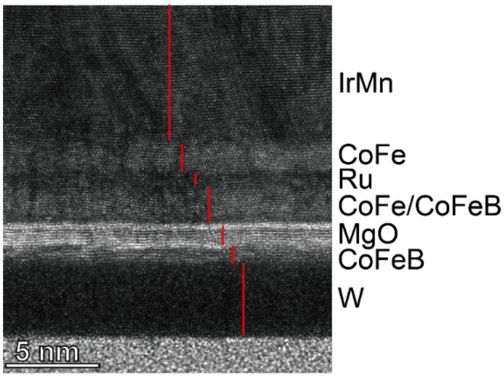


Figure R4. HRTEM image of MTJ stacks.

*Reviewer wrote*: (*Question 3)* It is recommended to include short-term stability test results or a discussion on future reliability study plans. This would significantly enhance the persuasiveness of the work from an application-oriented perspective.

*Our Response*:

Thank you for your constructive comments. Short-term stability test would make our proposal more persuasive for applications, and our device presents good stability, with no performance degradation after cycles test, which is discussed below.

At very beginning, it is necessary to declare that the measurement in the main manuscript is a reading process for magnetic state, and the relevant property is reading stability. Different from writing stability, where the device state is measured every hundreds or thousands of stimulations by a constant large current/voltage, reading stability of our proposal is determined by performing hundred-times of hysteresis loop measurements. Short-term reading stability is always much better than writing stability, as stimulated voltage is lower (**IEEE Trans. Magn., 2004, 40, 2631**). In our supplemented experiments, with power supply across lead 0 and 1 and sweeping in-plane field $H_x$, transverse voltage is measured between lead 2 and 4. Similarly, we monitored junction resistance as reference. We carried out 100 times measurements per day at one same device and repeated for 30 days. Notably, at first we used current supply of 5 μA (for normal readout as other researchers did) and changed to voltage supply of 300 mV at day 16 (for maximum readout).

**Figure R5a** shows the short-term stability at supply current of 5 μA. At 1$^{st}$ day and 1$^{st}$ test cycle, MTJ resistance ($R_{MTJ} \sim H_x$) and transverse voltage ($V_{xy} \sim H_x$) versus $H_x$ loop indicates $R_P$~4.75 kΩ, $R_{AP}$~8 kΩ, TMR~70%, transverse output voltage $\Delta V_{xy}$~0.07 mV. At 15th day and 1500th test cycle, $R_{MTJ}$ ($V_{xy}$) ~ $H_x$ loop presents little changes. **Figure R5b** shows the short-term stability at supply voltage of 300 mV. At 16th day and 1501th test cycle, $R_{MTJ}$ ($V_{xy}$) ~ $H_x$ loop indicates $R_P$ ~ 4.7 kΩ, $R_{AP}$ ~ 7.2 kΩ, TMR~53%, transverse output voltage $\Delta V_{xy}$ ~ 1.65 mV. TMR decrease than that at 5 μA is attributed to bias dependent spin tunneling (**Phys. Rev. B, 2002, 66, 174407**). At 30th day and 3000th test cycle, $R_{MTJ}$ ~ $H_x$ loop presents little changes, and $V_{xy}$ dropped by 0.01mV. This small drop can be attributed to measurement bias. **Figure R5c** presents the total trend of $R_P$, $R_{AP}$ and $\Delta V_{xy}$ during 30-day measurements. $R_{AP}$ experiences a drop at day 16, and $\Delta V_{xy}$ increases, because supply source is change from 5 μA to 300 mV. There is no obvious performance degradation after 3000 cycles test

in 30 days, indicating the excellent stability.

***Corresponding change in manuscript:*** *Yes*

*Location of Change: Part 2.3, Paragraph 5, Page#8*

The expression below has been added in Part 2.3: 'Towards applications, short-term stability is important for electronic device. In our proposal, the measurement is based on magnetic state readout process, which is quite different from writing stability. The reading stability of our device is similar with that of MTJ, always much better than writing stability. We carried out thousand-times of hysteresis loop measurements during 30 days, and results indicate there is no performance degradation (**Supplementary Material S8**).'

*Location of Change: Supplementary Material S8*

We have added **Supplementary Material S8** to explain short-term stability.

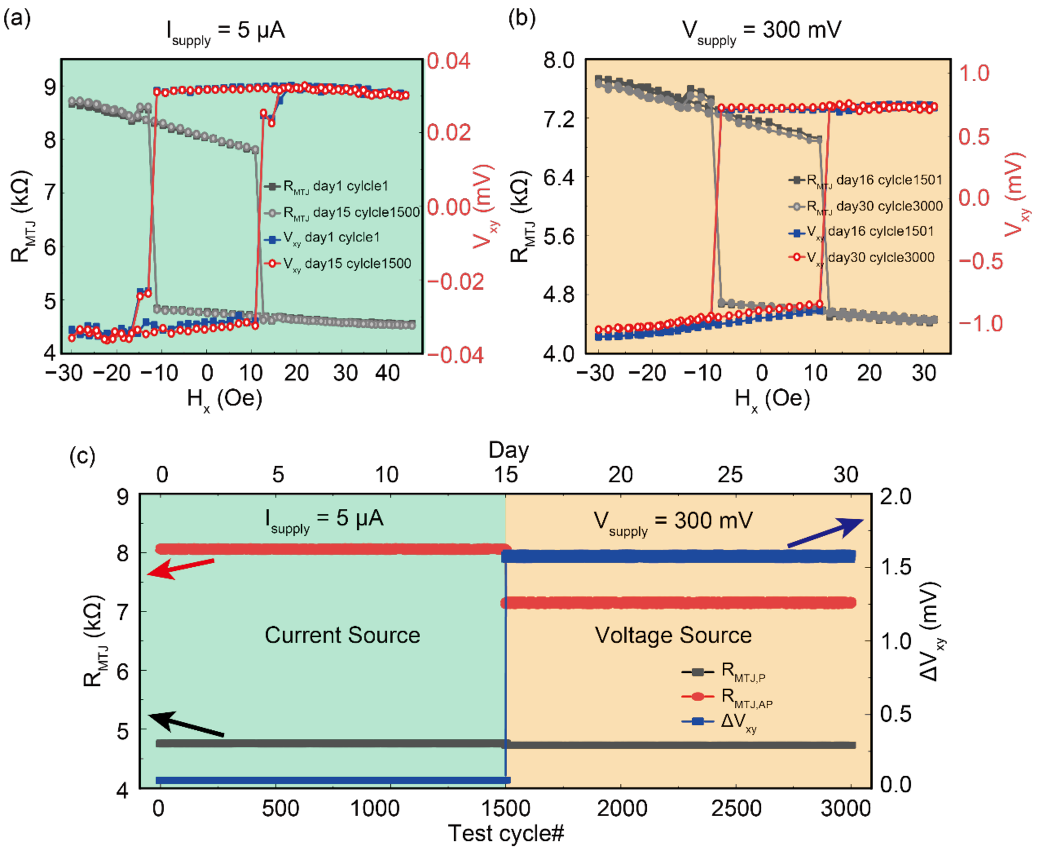


Figure R5. Evaluation of short-term stability. (a) Short-term stability at supply current of 5 μA. (b) short-term stability at supply voltage of 300 mV. (c) Total trend of $R_P$, $R_{AP}$ and $\Delta V_{xy}$ during 30-day measurements.

*Reviewer wrote*: (*Question 4)* In Figure 3, the spin-to-charge conversion signal exhibits asymmetry under positive and negative biases, which the authors attribute to the bias-dependent spin polarization rate. Are there more direct simulation data to support this explanation?

*Our Response*:

Thank you for your valuable suggestion regarding the bias-asymmetric spin-to-charge conversion (SCC) signal in Figure 3. We confirm that the observed asymmetry is strongly supported by direct simulation data and experimental results from published studies, which explicitly validate the bias-dependent spin polarization (SP) rate as the core mechanism.

On the one hand, we would like to validate the bias-dependent SP according to experimental reports. In our work, the observed SCC signal and TMR exhibit asymmetry under positive and negative biases and we speculated it originated from bias-dependent SP. Similar experimental results by Yuasa et al. (**Nat. Mater., 2004, 3, 868**) performed transport experiments on Fe/MgO/Fe MTJs. Their results showed that the TMR is distinctly bias-dependent: TMR reduces slower under positive bias than that under negative bias, with bias amplitude increases. As TMR is directly dependent on the SP, meaning that SP is also bias-related. Similar results with that of Yuasa are presented in our manuscript as **main text, Fig3d**, where $R_{MTJ,AP}$ reduces slower under positive bias than that under negative bias. This polarity-induced SP difference can lead to unequal SCC signal amplitudes, consistent with our experimental observation. Besides, this asymmetry is also observed by Liu. et. al in 2014 using W/MgO/CoFeB sample (**Nat. Phys., 2014, 10, 561**), where they suggested that its origination partially comes from bias-dependent SP.

On the other hand, first-principle calculations have indicated this bias-asymmetric SP. Xiang *et al.* calculated density of state (DOS) of majority and minority in MTJs, and obtained the theoretically different trend of SP with opposite energy (*i.e.* bias-dependent SP) (**Phys. Rev. B, 2002, 66, 174407**). Positive and negative biases activate different energy ranges of electronic states for tunneling, leading to unequal growth

rates of currents in parallel (P) and antiparallel (AP) magnetization configurations, thus causing asymmetric TMR and SP responses. Moreover, intrinsic interface asymmetry (barrier height/electronic structure differences) also leads to bias-asymmetric SP (**J. Phys. Condens. Matter, 2013, 25(49), 496005.**). MTJs inherently have asymmetric interfaces (*e.g.*, morphological roughness, oxidation differences, or interfacial modifiers). Positive and negative biases differentially regulate the effective barrier height at the two interfaces, amplifying disparities in interface transmission probabilities, which further exacerbates TMR and SP asymmetry.

In conclusion, the bias-asymmetric SCC signal in our work is validated by direct experiments and simulation data. These collectively confirm that the bias-dependent SP rate is the primary mechanism underlying the observed signal asymmetry, providing solid theoretical support for our explanation. In the revised manuscript, we have added the related discussion about this point.

***Corresponding change in manuscript:*** *Yes*

*Location of Change: Part 2.2, Paragraph 4, Page#6*

The expression below has been added in Part 2.2: ‘That is to say, as positive and negative biases activate different energy ranges of electronic states for tunneling, and regulate the effective barrier height at the two interfaces, amplifying disparities in interface transmission probabilities[44,45].’ and reference 44, 45 have been added.

*Reviewer wrote*: (*Question 5)* It is suggested that more details of device fabrication and simulation (such as the Hubbard U value) be provided in the Methods section.

*Our Response*:

Thank you for your detailed comments. It is important to present more details of fabrication and simulation in terms of reproduction of our research. We enrich these parts as blow.

On the one hand, we show more details about device fabrications. **Figure R6** shows the detailed process flow. For a nanoscale MTJ-enhanced readout device, we firstly patterned 500 nm × 2.5 μm cross-shape channels using standard electron beam

lithography (EBL) with maN2403 negative tone photoresist (300 nm thick after spin-coating at 3000 rpm for 60 s) and $Ar^+$ ion beam etching (IBE). The EBL exposure dose was optimized to 100 μC/cm² to ensure 500 nm-scale pattern fidelity, and post-exposure baking was performed at 110 °C for 90 s followed by development in maN Dev 525 for 30 s. During IBE milling, we monitored the secondary-ion mass spectra (SIMS) to precisely control the etching depth. The IBE process parameters for channel patterning were: 15° incident angle, 750 V acceleration voltage, 210 mA ion beam current, 150 mA neutralization current, -15 °C sample temperature, and 10 rpm sample stage rotation. After etching, we use acetone to remove the remaining photoresist. After that, a second round of EBL and IBE procedures were used to define elliptical junctions. The EBL exposure dose was optimized to 500 μC/cm² to ensure 60 nm-scale pattern fidelity. Thanks to the high-resolution and high-aspect-ratio properties of maN2403 photoresist, we successfully formed a photoresist pillar with tens-nanometer length and hundreds-nanometer height. The junction etching was divided into two phases: first, we defined its appearance by small angle etching (15°) until the bottom 3.5-nm-thick W signal appears in SIMS; second, large angle etching (75°) was utilized to meticulously clean the sidewall re-depositions across the barrier, under 140 mA ion beam current and 70 mA neutralization current.

Rather than removing the photoresist immediately, we covered the pillars with a 50-nm-thick silicon nitride ($Si_3N_4$) passivation layer by inductively coupled plasma chemical vapor deposition (ICP-CVD) under the conditions: 1 Pa chamber pressure, 350 W ICP power, 0 W RF bias power, 131 sccm $SiH_4$ flow, 8.6 sccm $NH_3$ flow, 126 sccm Ar flow, and 100 °C substrate temperature. Then lift-off process was conducted by soaking in N-mettohylpyrrolidone (NMP) at 90 °C for 2 h, followed by ultrasonic cleaning in IPA and deionized water for 2 min and 1 min respectively remove the photoresist. Vias through bottom electrodes were formed by standard ultraviolet lithography with MICROPOSIT S1813 photoresist and inductively coupled plasma reactive ion etching (ICP-RIE). The ICP-RIE parameters for $Si_3N_4$ etching were: 10 mTorr chamber pressure, 20 °C temperature, 5 sccm $SF_6$ flow, 5 sccm Ar flow, 100 W ICP power, and 3 W RF power, achieving an etching rate of 60 nm/min. Ti (10 nm)/Au

(100 nm) electrodes were finally formed by E-beam evaporation (EBE) with a deposition rate of 0.5 Å/s for Ti and 2 Å/s for Au, followed by lift-off in acetone.

On the other hand, we discuss simulation details. The physical quantities are represented using a linear combination of atomic orbitals (LCAO) basis with double-zeta plus polarization (DZP) functions. A 20×20×1 k-point mesh is employed in the self-consistent calculation, with the Hamiltonian matrix converged to a tolerance of $10^{-4}$ eV. Regarding the simulations employing PBE-GGA pseudopotentials, we did not apply a Hubbard U value because the physical and technical conditions that justify DFT+U are not met in this system. DFT+U is intended to remedy orbital-dependent self-interaction errors for strongly localized, partially filled d/f shells. In the W/CoFe/MgO/CoFe/W structure the CoFe layers are metallic and strongly hybridized with W and interface states so that the relevant 3d electrons behave as itinerant rather than atomic-like. Imposing a Hubbard U value would risk artificially shifting d-band energies, altering hybridization and Fermi-surface character, and thereby producing spurious changes in the symmetry-filtered transmission channels that control tunneling. DFT+U also complicates NEGF because U depends on local orbital occupancies that go out of equilibrium under bias, leading to convergence instability and potential spurious interface scattering if U is applied inconsistently. For these reasons we chose PBE-GGA without a Hubbard U as the most physically consistent and transparent baseline for the transport calculations.

In conclusion, details of Methods are important for reproduction. We enrich this part by providing more precise descriptions and parameters.

***Corresponding change in manuscript:*** *Yes*

*Location of Change: Part Methods, Page #11 and #12*

We have supplemented some device fabrication and ab-initio simulation details in Part **Methods**.

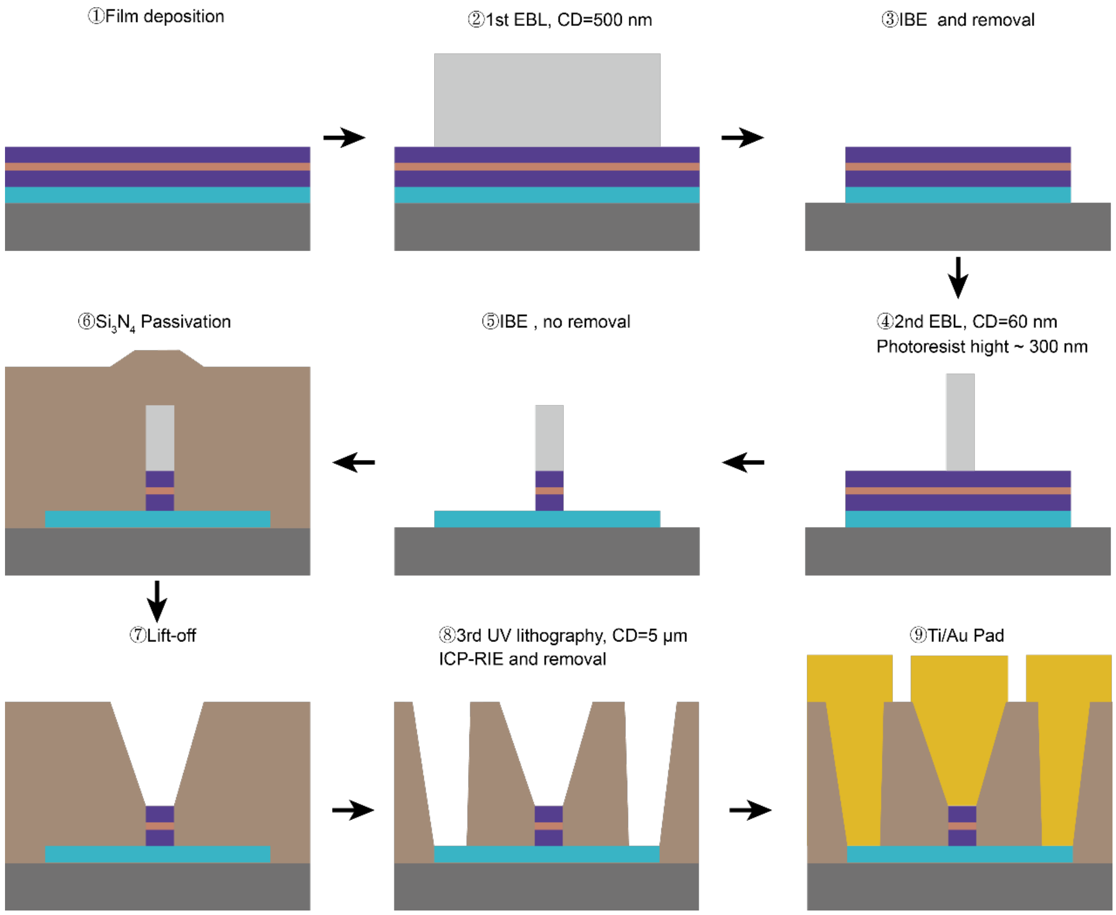

Figure R6. Process flow for device fabrication.

*Reviewer wrote*: (*Question 6)* The authors mention that the MTJ enables "instantaneous data exchange", but have not tested the timing characteristics of magnetic state switching. The lack of direct data undermines the rigor of the work.

*Our Response*:

Thank you for pointing out this critical comment. Regarding the concern that the "instantaneous data exchange" enabled by MTJ lacks timing characteristic tests, we supplement the explanation as below.

Firstly, consistent switching speed of the free layer guarantees high-speed response. MTJ in proposed device acts as an amplifier in reading process and enhances $V_{out}$, but plays no role in writing process. The MTJ-enhanced MESO shares the same FM free layer (CoFeB) as the traditional MESO, and both rely on ME-driven domain switching for magnetization reversal. Previous studies have pronounced that the ferromagnetic layer in traditional MESO can achieve nanosecond switching speed

(**Nature, 2019, 565, 35; IEEE IEDM, 2022, 36.4.1.**). In this work, the integration of MTJ does not alter the material composition, thickness, or magnetic anisotropy of the free layer—only optimizing the spin polarization rate through the spin filtering effect. Thus, the free layer switching speed of the MTJ-enhanced MESO is consistent with that of the traditional MESO, providing a physical basis for high-speed response.

Secondly, in-situ storage enables inherently synchronous data exchange. A core advantage of the MTJ-enhanced MESO lies in the in-situ integration of logic computation and data storage. The logic results of MESO are directly recorded in the MTJ through changes in the magnetic state of the free layer, eliminating the need for additional data transfer or conversion steps. This "computing-storage integration" architecture avoids transmission delays in traditional separated logic-storage architectures, where data exchange occurs synchronously with logic computation. This inherently realizes the "instantaneous" feature without requiring extra timing calibration.

We acknowledge the importance of timing tests for practical applications. In future work, we will quantify the free layer switching time (targeting sub-nanosecond switching verification) through pulsed magnetic field tests and high-speed electrical characterization. However, the current experiments emphasize large output signal and have confirmed the functional compatibility of MTJ and MESO output through TMR response, which is sufficient to support the conclusion that "integrating MTJ can achieve efficient data exchange."

***Corresponding change in manuscript:*** *Yes*

*Location of Change: Part 3 Paragraph 3 Page#10*

The expression ‘This device can operate as a logic unit with an intrinsically-integrated high-speed cache memory to resolves the bandwidth mismatch between logic unit and external memory’ **has been modified to** ‘This device can operate as a logic unit with an intrinsically-integrated high-speed cache memory, as a shared free layer CoFeB is used for logic and memory simultaneously, which resolves the bandwidth mismatch between logic unit and external memory’.

*Reviewer wrote*: (*Question 7)* In Fig. 2b, "the RA value increases with increasing MgO thickness, while the TMR ratio exhibits an initial increase followed by a decrease". As the thickness of the insulating layer increases, scattering intensifies, and the TMR should keep rising. Why does the TMR first increase and then decrease? What are the implications of the increased TMR (Tunneling Magnetoresistance) ratio and spin polarization in the MTJ structures for the efficiency of the MESO logic system?

*Our Response*:

Thank you for your valuable comments, which are crucial for in-depth explanation of the research mechanism and core value. Below is a detailed point-by-point response.

1. Reason for the initial increase followed by a decrease in TMR as MgO thickness increases.

TMR oscillation with varying MgO thickness can be attributed to coherent tunneling and superposition effect of wave functions. This oscillation was observed in 2004 by Yuasa *et al.* (**Nat. Mater., 2004, 3, 868**) and reproduced by other researchers (**Appl. Phys. Lett., 2023, 122, 112404**). The crystalline quality of MgO barrier is significantly improved at the initial stage of increasing MgO thickness. MgO forms a more regular (001)-oriented interface with the FM layer, and the density of defects such as oxygen vacancies is reduced. This interface optimization enhances the Δ1 band coherent tunneling, which only allows efficient tunneling of majority-spin electrons and suppresses the tunneling of minority-spin electrons, thereby greatly improving the spin polarization rate. Meanwhile, according to the theoretical model in "Theory for tunnel magnetoresistance oscillation" (**Phys. Rev. B, 2025, 111, L220406**), the superposition effect of wave functions between majority-spin Δ1 state and minority-spin Δ2 state induced by spin-flip scattering is weak at this stage, and tunneling transport is dominated by coherent tunneling. Thus, the optimization of interface quality becomes the dominant factor for the increase of TMR.

When the MgO thickness exceeds 1.1 nm, the electron tunneling probability of MgO decreases exponentially with increasing thickness. Moreover, electron scattering intensifies in the thick barrier, leading to a significant reduction in effective tunneling

current and a marked weakening of spin-polarized tunneling signals. Quantum interference effect would play a dominate role in TMR evolution (**Phys. Rev. B, 2025, 111, L220406**). Interfacial spin-flip scattering can induce the superposition and interference of wave functions between majority-spin Δ1 state and minority-spin Δ2 state. When the MgO thickness increases to a certain extent, this quantum interference no longer enhances the spin filtering effect, but instead reduces the difference in tunneling transmittance between the parallel (P) and antiparallel (AP) states, and then the trend would reverse with increasing MgO thickness. However, in the MgO thickness range of this experiment (0.9-1.4 nm), the obvious oscillation stage has not been entered, so it mainly shows a "first increase and then decrease" trend, which is consistent with the above multi-factor competition mechanism.

2. Implications of increased TMR ratio and spin polarization in MTJ structures for the efficiency of MESO logic systems.

These implications can be divided into two parts, implications under current source and implications under voltage source. For current source, efficiency between output charge current and supply charge current is defined as

$$\eta_I = \frac{\Delta V_{xy}/R_{13}}{2I_{supply}} \times 100\% = \frac{\Delta R_{scc}}{R_{13}} \times 100\% \quad \text{(R16)}$$

According to **Supplementary Material S11**, $\Delta R_{scc}$ is given by

$$\Delta R_{SCC} = \frac{SP\theta_{SH}\lambda_{SOC}}{\left(\frac{t_{FM}}{\rho_{FM}}+\frac{t_{SOC}}{\rho_{SOC}}\right)w_{SOC}} \times \frac{1-\frac{1}{cosh\left(\frac{t_{SOC}}{\lambda_{SOC}}\right)}}{tanh\left(\frac{t_{SOC}}{\lambda_{SOC}}\right)+\frac{\lambda_{SOC}\rho_{SOC}}{\lambda_{FM}\rho^{*}{}_{FM}}tanh\left(\frac{t_{FM}}{\lambda_{FM}}\right)} \quad \text{(R17)}$$

This means that $\eta_I$ is proportional to spin polarization rate SP, thus increases with TMR increasing.

According to **Supplementary Material S7**, efficiency between output voltage and supply voltage for voltage source is defined as

$$\eta_V = \frac{\Delta V_{xy}/2}{V_{supply}} \times 100\% = \frac{(1+TMR)R_{xy}^{P}-R_{xy}^{AP}}{2R_{MTJ}^{AP}} \times 100\% \quad \text{(R18)}$$

To get higher $\eta_V$, one should employ stacks with higher TMR and lower $R_{MTJ}^{AP}$, which means using MgO thickness at first peak of TMR oscillation curve.

In conclusion, TMR trend of firstly increasing and then decreasing is at the original stage of TMR oscillation curve, which can be attributed to coherent tunneling and

superposition effect of wave functions. Increasing TMR ratio can always increase the efficiency of MESO output under supply current source, but for voltage source, MESO output efficiency reaches maximum at first peak of TMR oscillation curve.

***Corresponding change in manuscript:*** *Yes*

*Location of Change: Part 2.1, Paragraph 1, Page #4*

Original expression 'Note that, TMR is expected to appear oscillations as ***t*** keeps increasing due to coherent tunneling effect' **has been modified to** 'Note that, TMR is expected to appear oscillations as t keeps increasing due to coherent tunneling and superposition effect of wave functions[30-32]', and reference 32 has been added.

*Location of Change: Part 2.3, Paragraph 3, Page #7 #8*

The following sentences have been added in Part 2.3: 'These demands prefer MgO thickness at first peak of TMR oscillation curve, which also brings maximum conversion efficiency from $V_{supply}$ to $V_{out}$ (see **Supplementary Material S7**)'.

*Location of Change: Supplementary Material S7*

We added **Supplementary Material S7** to explain TMR implications for supply-out conversion efficiency.

*Reviewer wrote*: (*Question 8)* In Fig.5c and 5d, the data of the analog devices are overly simplistic, lacking a large number of parameters. There is even no information on the lattice matching of the scattering region and the electrode region, as well as the spacing between various parts after device optimization, etc. Good work should be reproducible, and the author needs to complete all the data supplementation.

*Our Response*:

Thank you for pointing out this critical comment. We agree that a detailed description of our structure should be provided. Detailed structure of MTJ in the simulation is discussed as below.

To investigate the difference in spin polarization between CoFeB and MTJ, we consider W/CoFe/W and W/CoFe/MgO/CoFe/W structures in the ab-initio calculations, as shown in Fig. 5c and 5d. To minimize the lattice mismatch, we employ a face-

centered cubic structure for W [100] deposited on the CoFe [100] surface, while the tunneling layer MgO is oriented along [110]. The in-plane lattice constants of the two structures are set to 2.83 Å, consistent with bulk CoFe. The W/CoFe/W structure contains 19 monolayers (19 ML) of CoFe between the W leads, while the scattering region of W/CoFe/MgO/CoFe /W is composed of 5 ML CoFe, 5 ML MgO, and 5 ML CoFe. After relaxation, the optimized distances are 1.64 Å between the interfacial W and Co layers, as well as 1.97 Å between MgO and Co. As a result, the scattering region lengths of the two structures are comparable, with 26.33 Å for W/CoFe/W and 26.51 Å for W/CoFe/MgO/CoFe/W.

***Corresponding change in manuscript:*** *Yes*

*Location of Change: Part 2.4, Paragraph 4, Page #9*

The following sentences have been added in Part 2.4: 'Detailed structure in the simulation is presented in **Methods** and **Supplementary Material S12**'.

*Location of Change: Supplementary Material S12*

We added **Supplementary Material S12** to explain detailed structure of MTJ in the simulation.

*Reviewer wrote*: (*Question 9)* The subheading of Part 2.3 is: Mechanism Explorations. However, only the matching states of the wave functions of the majority electrons and the minority electrons in the Brillouin zone are described. This part merely describes the phenomenon and does not provide sufficient explanation of the physical mechanism. The author should improve this aspect.

*Our Response*:

Thank you for your valuable comment, which accurately points out the insufficiency in the explanation of the mechanism. We fully agree that the "Mechanism Explorations" part needs to delve into the physical essence rather than merely describe phenomena. We will supplement and improve it from the following aspects to clearly clarify the core physical mechanism of the giant readout signal.

Firstly, we declare that there are two phases in magnetic state readout: SCC phase

and spin polarization phase. High-performance W channel works at SCC phase, spin filtering functions at spin polarization phase under supply current source, and both spin filtering and current modulation function at spin polarization phase under supply voltage source.

For SCC phase, the key factor is performance of W channel. We performed spin-torque ferromagnetic resonance (ST-FMR) measurement on W/CoFeB (5nm) heterojunction, with different W thickness (**Supplementary Material S9**). It was found that 3.5 nm W presents as large spin Hall angle as -0.26, promising for high SCC signal.

To investigate spin polarization phase, we compared the output transverse signal in proposed MTJ-enhanced device and traditional FM/SOC-bilayer device under current source. With the same channel width, measurement signals of these two devices present 600 times difference. Based on the one-dimensional spin diffusion model in **Supplementary Material S11**, the quantitative impact of key parameters on the output signal is clarified: 1) The increase in spin polarization rate (P) from 0.2 (ordinary FM layer) to 0.92 (MTJ) directly leads to an approximately 4.6-fold enhancement of the SCC signal; 2) Because there is a phase change when the thickness of W changes from 6 nm to 3.5 nm, the W resistivity in sample 1 is about 60 μΩ·cm and that in sample 2 is about 367 μΩ·cm. The synergistic effect of thickness and resistivity of the W leads to approximately 7.9 times difference; 3) The $\theta_{SH}$ decreases for W from 0.26 at 3.5 nm to 0.04 at 6 nm, yielding 6.5 times difference. 4) FM thickness difference yields 1.7 times difference. These factors give about 400 hundreds-times of difference between these two scenarios, as depicted in **Fig 5b**, quite matching the experiments.

In addition, for measurements under voltage source $V_{supply}$, MTJ not only increases SP, but also modulates injection current between $I_{AP}$ and $I_P$. The resistance difference between the parallel (P) and antiparallel (AP) states of MTJ (TMR=70%) leads to significant change in spin injection. A high spin-polarized current ($I_P \approx 51$ μA) is injected into the SOC layer in the P state at 300 mV, forming a huge current difference ($\Delta I \approx 15$ μA) with the low spin-polarized current ($I_{AP} \approx 36$ μA) in the AP state. This current difference is superimposed with the transverse voltage generated by the SCC

effect, and the signal is amplified through the formula $\Delta V_{xy}= [(1+TMR)R_{xy}^{P} - R_{xy}^{AP}]V_{supply}/R_{MTJ}^{AP}$. The superposition of the above factors and the current modulation effect of MTJ ultimately achieves a breakthrough in the output voltage from the microvolt level (<20 μV) to the millivolt level (1.5 mV).

In conclusion, the revised "Mechanism Explorations" part has fully clarified the physical mechanism of MTJ-enhanced MESO readout signals from three levels: high-performance W channel, spin filtering under supply current source, and current modulation under supply voltage source.

***Corresponding change in manuscript:*** *Yes*

*Location of Change: Part 2.4, Paragraph 1 and 4, Page #8 and #9*

We have added expression below in *Part 2.4.*

‘As we mentioned in Part 2.1, this reading process is divided into spin polarization phase and SCC phase. For SCC phase, the key factor is performance of W electrode. We performed spin-torque ferromagnetic resonance (ST-FMR) measurement on W/CoFeB (5nm) heterojunction, with different W thickness (**Supplementary Material S9**). It was found that 3.5 nm W presents as large spin Hall angle as -0.26, promising for high SCC signal.’ (Page #8)

‘In addition, for measurements under voltage source $V_{supply}$, MTJ not only increases SP, but also modulates injection current between $I_{AP}$ and $I_P$. The current difference

$$\Delta I = I_P - I_{AP} = V_{supply}\left(\frac{1}{R_{MTJ}^{P}} - \frac{1}{R_{MTJ}^{AP}}\right) = TMR \cdot I_{AP} \tag{2}$$

brings different spin current injection to W channel and induces extra transverse output. This extra term directly enhances $\Delta V_{xy}$ and, after subscribing offset voltage, $\Delta R_{xy}$ is also enhanced (see **Supplementary Material S5**).’ (Page#9)

We are very grateful for your constructive comments and acknowledgement on this work. Your suggestions helped us improve the clarity and quality of this work, and lead the direction of future research. We sincerely hope that the point-by-point response

will effectively address your concerns and that the revised manuscript will fully meet your requirements. Thank you again for your patience and detailed comments.

***Reviewer #3***

***Comment #***

*Reviewer wrote*: In this manuscript, the authors present the integration of MESO and MTJ devices, achieving an output voltage of approximately 1.5 mV. This result indicates a measurable improvement in readout performance and represents a step toward the practical implementation of MESO technology. However, the evidence supporting the claimed "robust ISHE readout" remains limited, and additional quantitative analysis would strengthen the manuscript. With further clarification and experimental validation, the work could make a meaningful contribution to the field.

*Our Response:*

We greatly appreciate your positive assessment of this work, and your constructive suggestions which helped us improve the clarity and quality of this manuscript.

*Reviewer wrote*: (*Question 1)* From Eq. (1) derived by the author in the main text, $V_{supply}/R_{MTJ, AP}$ is equal to $I_{AP}$, and $I_{AP}$ seems dominant over the $\Delta V_{xy}$ in the MTJ device with a voltage supply source. How was the current amplitude under the AP state for the voltage source in Fig. 4a and 4b? The $\Delta R_{xy}$ in Fig. 4b is discrete in positive supply voltage, while flat in the negative supply voltage. Consider the variation of SP rate may not be enough to account for the large changes in the output signal, as shown in ref. 36. Does it originate from the voltage dependence of current?

*Our Response*:

Thank you for your detailed comments. $I_{AP}$ seems dominant over the $\Delta V_{xy}$ but actually not, and $\Delta R_{xy}$ trend under voltage supply is attributed to offset Hall voltage and data process. Below is a detailed point-by-point response.

We firstly clarify AP state current. Every supply voltage corresponds to a specific AP current when the field switches MTJ to AP state, and which is similar for P state. This means $I_{AP}$ is not dominant over the $\Delta V_{xy}$, although it seems to be in Eq. (1). For example, when $V_{supply}$ = 300 mV, $R_{01}=R_{MTJ}+R_{BE/2}$, where $R_{BE}$ representing the resistance of bottom electrode is around 2.2 kΩ, AP state current is around $I_{AP}$=0.3/(7.2+1.1) mA = 0.036 mA, and P state current is around $I_P$=0.3/(4.7+1.1) mA

= 0.051 mA. In the insert of **Figure R7(a)**, we highlight the corresponding current in blue when the source is constant voltage 300 mV. The difference between $V_{xy,P}$ and $V_{xy,AP}$ is around 1.5 mV with 300 mV supply, which is more than 3 times of that under 40 μA supply. This enhancement is attributed to the difference of $I_P$ and $I_{AP}$, not only $I_{AP}$. Given that $\Delta V_{xy}$ is proportional to the amplitude of the injection tunneling current, under a supply current of 51 μA ($I_P$ @ 300 mV supply), $\Delta V_{xy}$ is estimated to be around 0.51 mV, which is much less than that measured under 300 mV supply. This means current modulation by MTJ resistance state contributes a lot to transverse output voltage, rather than $I_{AP}$ dominates output. We supplemented the corresponding current $I_{AP}$ amplitude in **Figure R7(b)** as top axis.

Then, we explain $\Delta R_{xy}$ trend under voltage supply. $\Delta R_{xy}$ trend picture under voltage supply aims to prove the current modulation by MTJ from another perspective besides of output voltage $\Delta V_{xy}$, and this trend is attributed to offset Hall voltage and data process. We pronounce firstly that output voltage is estimated by $\Delta V_{xy} = V_{xy,max} - V_{xy,min} = (V_{SCC,max}+V_{offset}) - (V_{SCC,min} + V_{offset}) = V_{SCC,max} - V_{SCC,min}$. In this scenery, $V_{xy,max}$ is denoted by $V_{xy,P}$, and $V_{xy,min}$ by $V_{xy,AP}$. There is offset voltage $V_{offset}$ mixed in measured signal, which originates from device structural asymmetry, material/interface inhomogeneities, parasitic effects in the measurement system, and limitations of calibration methods. The true SCC signal $V_{SCC,max}$ and $V_{SCC,min}$ are equal to $\Delta V_{xy/2}$ and $-\Delta V_{xy/2}$, respectively, which is directly used for cascading. When we come to estimate the $\Delta R_{xy}$ trend under voltage supply, whether we modify the raw data to ensure $V_{offset}$ is subtracted or not would influence the presented results. Take data under 200 mV and -200 mV supply as example. Here, we show two different estimations. As shown in **Figure R7(c)** and **(d)**, raw transverse voltage data under 200 mV and -200 mV spreads across 1.2 mV to 2.8 mV and -3.6 mV to -2.0 mV, respectively, corresponding to $\Delta R_{xy}$ around 15 Ω@200 mV and 4 Ω@-200 mV. However, when we exclude $V_{offset}$ from raw data, the modified output transverse voltage spreads across -0.8 mV to 0.8 mV for both 200 mV and -200 mV supply, as shown in **Figure R7(e)**. As a result, the modified $R_{xy}$ v.s. $H_x$ loops are more closed for opposite voltage supply as shown in **Figure R7(f)**. The corresponding modified $\Delta R_{xy}$ is 42.4 Ω@200 mV and 39.2 Ω@-200 mV. We show

the modified $R_{xy}$ v.s. $H_x$ with supply voltage from -200 mV to 300 mV in **Figure R7(g)** and $\Delta R_{xy}$ trend vs supply voltage as **Figure R7(h)**. For modified Hall resistance signal, $\Delta R_{xy}$ curve is almost smooth for both positive and negative supply voltage. And, this trend is agreement with that shown in **Figure 3(c)** in the main manuscript.

In conclusion, $\Delta V_{xy}$ is not dominated by $I_{AP}$, but by both $I_{AP}$ and $I_P$. $\Delta R_{xy}$ shows very different trend is caused by offset Hall voltage and data process.

***Corresponding change in manuscript:*** *Yes*

*Location of Change: Part 2.3, Paragraph 2, Page#7*

Original expression 'As shown in Fig. 4b, the relationship between $\Delta R_{xy}$ and voltage supply exhibits no regularity, quite different from that depicted in Fig. 3c, meaning an extra term brought by TMR signal, in agreement with our analysis.' **has been modified to** 'As shown in **Fig. 4b**, the relationship between $\Delta R_{xy}$ and voltage supply exhibits no regularity, quite different from that depicted in **Fig. 3c**, this is because offset Hall voltage. When this offset is subscribed, $\Delta R_{xy}$ trend v.s voltage supply is similar with Fig. 3c, but with larger value (see **Supplementary Material S5**), meaning an extra term brought by TMR signal, in agreement with our analysis.'

*Location of Change: Part 2.4, Paragraph 4, Page#9*

We have added expression below in *Part 2.4.*

'In addition, for measurements under voltage source $V_{supply}$, MTJ not only increases SP, but also modulates injection current between $I_{AP}$ and $I_P$. The current difference

$$\Delta I = I_P - I_{AP} = V_{supply}\left(\frac{1}{R_{MTJ}^{P}} - \frac{1}{R_{MTJ}^{AP}}\right) = TMR \cdot I_{AP} \quad (2)$$

brings different spin current injection to W channel and induces extra transverse output. This extra term directly enhances $\Delta V_{xy}$ and, after subscribing offset voltage, $\Delta R_{xy}$ is also enhanced (see **Supplementary Material S5**).' (Page#9)

*Location of Change: Supplementary Material S5*

We added **Supplementary Material S5** to explain offset Hall voltage and data

process influence on $\Delta R_{xy}$.

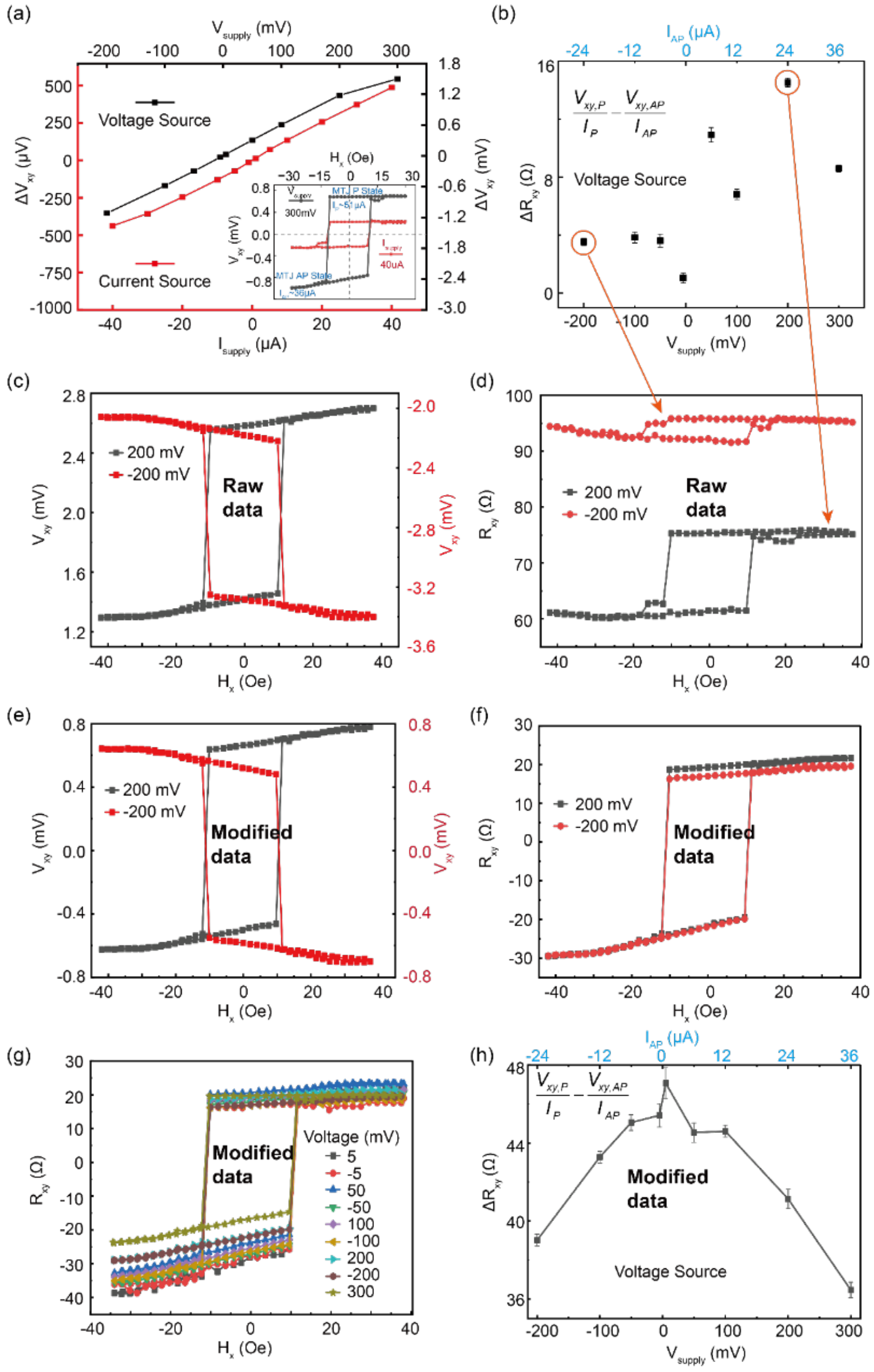


**Figure R7**. Offset Hall voltage influence. (a) Output voltage comparison between voltage supply and current supply. Inset: Transverse voltage under 300 mV v.s. that under 40 μA. (b) Resistance outputs from raw data at different supply voltages. (c) Raw data of transverse voltage v.s. field at ±200 mV supply. (d)Transverse resistance v.s. field from measurement raw data at ±200 mV supply. (e) Modified data of transverse voltage v.s. field at ±200 mV supply. (f)Transverse resistance v.s. field from modified

data at ±200 mV supply. (g) Transverse resistance v.s. field at different supply voltages. (h) Resistance outputs from modified data at different supply voltages.

*Reviewer wrote*: (*Question 2)* The output signal of the MTJ-enhanced device at $I_{supply}$ = 10 μA in Fig. 5a was quite smaller than the value of the EBL device using current sources in Fig. 3. Also, the squareness of the hysteresis loop was decreased in the device depicted in Fig. 5. What is the dimension of the microdot device?

*Our Response*:

Thank you for your detailed comment.

The signal in Fig. 5a (1.2 Ω) is smaller than the value in Fig. 3 (13.5 Ω), which originates from size scaling. The device used in Fig. 3 is 60 nm × 130 nm nanodot with 500-nm-width channel, while that in Fig. 5a is 1 μm× 2.5 μm microdot with 3-μm-width channel. The microdot design is shown in **Figure R8**. If we only considering the one-dimensional spin diffusion numerical model (see **Supplementary Material S11**), the channel width difference will yield about 6 times difference. Considering about the scaling of FM dot and contact area between FM and SOC layer, it is reasonable to get 10 times difference in measurements.

The squareness of the hysteresis loop was decreased in the device depicted in Fig. 5, which is originate from FM size increasing. With larger size, FM layer could present multi domain, and thus decreases the squareness of the hysteresis.

***Corresponding change in manuscript:*** *Yes*

*Location of Change: Part 2.4, Paragraph 2, Page #8*

To clearly present device under test, original expression 'We designed some control experiments using microdot devices with the same channel width of 3 μm.' **has been modified to** 'We designed control experiments using 1 μm × 2.5 μm microdot devices with the same channel width of 3 μm'.

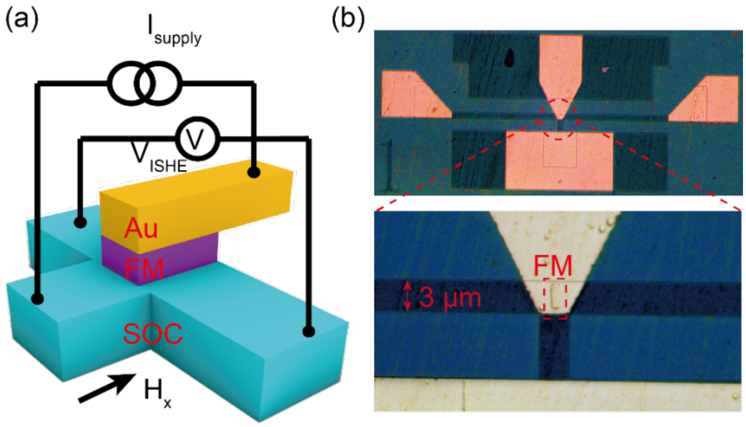


**Figure R8**. Microdot device structure. (a) Schematic of device measurement set-up. (b) microscope of top view.

*Reviewer wrote*: (*Question 3)* The substantially increased output signal originating from the MTJ modulation was investigated. However, in the mechanism explorations, the author claimed the difference between the MTJ sample and the control sample was due to the spin polarization (SP), resistivity, and spin Hall angle ($\theta_{SH}$). Given two conflicting statements, please explain which one is the main contributor, and how do you quantify it?

*Our Response*:

Thank you for raising this critical question, which helps clarify the mechanism more clearly. In the original version of mechanism explorations, difference between the MTJ sample and the control sample is aim to emphasize the importance of SP and spin-orbit-coupling (SOC) channel quality, and these measurements are carried out under current supply. Things are different under voltage supply because of current modulation of MTJ. In brief, spin filtering by MTJ is critical at current supply, both spin filtering and current modulation by MTJ are critical at voltage supply, and high-performance SOC channel is always critical. The difference of output signal under current source and voltage source is discussed as below.

There are two measurement mode in our work: current supply using a constant current injection and voltage supply using a constant voltage source. There are three critical factors in measurements: spin filtering of MTJ, current modulation of MTJ, high

SOC of W. For current supply, the resistance change of MTJ does not modulate injection current, only spin filtering and SOC work. For voltage supply, the resistance change of MTJ alters injection current, spin filtering, current modulation and SOC all function. Thus, under voltage supply, output signal is larger than that under current supply for similar current amplitude.

Next, we turn to quantify contributions of each part. For current source $I_{supply}$, according to one-dimensional spin diffusion numerical model(see **Supplementary Material S11**), $\Delta V_{xy} = V_{xy}^{P} - V_{xy}^{AP} = I_{supply}R_{xy}^{P} - I_{supply}R_{xy}^{AP} = I_{supply}\Delta R_{xy} = 2I_{supply}\Delta R_{SCC} \propto \frac{2I_{supply}SP\cdot\theta_{SH}}{w_{SOC}}$ (**Nat. Electron. 2020, 3, 309**), output voltage is proportional to spin polarization rate SP and spin Hall angle $\theta_{SH}$, and inversely proportional to SOC channel width $w_{SOC}$.

For voltage source, $\Delta V_{xy} = I_{AP}\Delta R_{xy} + TMR \cdot I_{AP}R_{xy}^{P}$ (see Supplementary Material S6), the first term is equal to the signal under a current source of $I_{AP}$ (spin filtering), and the second term is an extra signal by MTJ current modulation. For ideal device with no offset Hall voltage, assuming that $\Delta R_{xy} = 2R_{xy}^{P}$, and TMR=150%, the ratio of the first term to the second term is 4:3. This means there is no dominate factor, but improving TMR is helpful for both spin filtering and current modulation.

In conclusion, the original version manuscript may cause confusion in mechanism, but there is no actual conflict in these key factors. Some or all of these factors function at different measurement mode, and there is no truly dominant one. In the revised manuscript, we have clarified this point.

***Corresponding change in manuscript:*** *Yes*

*Location of Change: Part 2.4, Paragraph 1, 2 and 4, Page #8 and #9*

We have added expression below in *Part 2.4.*

'As we mentioned in Part 2.1, this reading process is divided into spin polarization phase and SCC phase. For SCC phase, the key factor is performance of W electrode. We performed spin-torque ferromagnetic resonance (ST-FMR) measurement on

W/CoFeB (5nm) heterojunction, with different W thickness (**Supplementary Material S9**). It was found that 3.5 nm W presents as large spin Hall angle as -0.26, promising for high SCC signal.' (Page #8)

'In addition, for measurements under voltage source $V_{supply}$, MTJ not only increases SP, but also modulates injection current between $I_{AP}$ and $I_P$. The current difference

$$\Delta I = I_P - I_{AP} = V_{supply}\left(\frac{1}{R^P_{MTJ}} - \frac{1}{R^{AP}_{MTJ}}\right) = TMR \cdot I_{AP} \tag{2}$$

brings different spin current injection to W channel and induces extra transverse output. This extra term directly enhances $\Delta V_{xy}$ and, after subscribing offset voltage, $\Delta R_{xy}$ is also enhanced (see **Supplementary Material S5**).' (Page#9)

Original expression 'At first, we compared the output transverse signal in proposed MTJ-enhanced device and traditional FM/SOC-bilayer device.' **has been modified to** 'To investigate spin polarization phase, we compared the output transverse signal in proposed MTJ-enhanced device and traditional FM/SOC-bilayer device.' (Page #8)

*Reviewer wrote*: (*Question 4)* The authors should clarify their rationale for selecting W (6 nm) as the control sample instead of W (3.5 nm). Given that increasing the W thickness typically enhances electrical conductivity while reducing the spin-to-charge conversion efficiency. Whether the robust ISHE readout can be maintained when replacing W (3.5 nm) with W (6 nm) in the MTJ-enhanced device?

*Our Response*:

Thank you for your detailed comments. We selected W thickness of 6 nm instead of 3.5 nm is based on the follow consideration. Firstly, according to **Supplementary Material S11**, for any material with known parameters, the output resistance signal $\Delta R_{SCC}$ can be inferred regardless of its thickness, and data of W(6)/CoFeB(5) is ready in our other experiments; Secondly, for bilayer W/CoFeB structure, over etching process demands thicker W. We explain in detail below.

Firstly, we compared output signals in 2 samples: Pt(6)/CoFeB(5) and

W(6)/CoFeB(5). We found that signal with W is larger than that with Pt, and polarities are opposite, as shown in **Figure R9(a)**. These experimental data can be reused in our control experiments in the main manuscript. By using one-dimensional spin diffusion numerical model and parameters shown in **Supplementary Material S11**, we can compare the results shown in Figure 5(a) quantitatively.

Secondly, as shown in **Figure R9(b)**, for W/MTJ sample, endpoint detector functions easily as SIMs signal of Mg is strong. Considering of 50% over-etching process, when the etching is across Mg, we operate carefully to found weak W signal. For W/CoFeB sample, SIMs signal for CoFeB and W are both weak, etching depth is hard to control. So, we selected thicker W of 6 nm as SOC material to ensure the quality of fabricated devices and eliminate the unrelated factors. As to maintaining robust ISHE readout in the MTJ-enhanced device with W (6 nm), this can be possible because the signal is certainly larger than that in W/CoFeB bilayer. But, signal with W (6 nm) is no doubt smaller than that with W (3.5 nm) from the point of spin Hall angle and resistivity.

***Corresponding change in manuscript:*** *Yes*

*Location of Change: Supplementary Material S10*

We added **Supplementary Material S10**, which explained the details of bilayer microdot readout device.

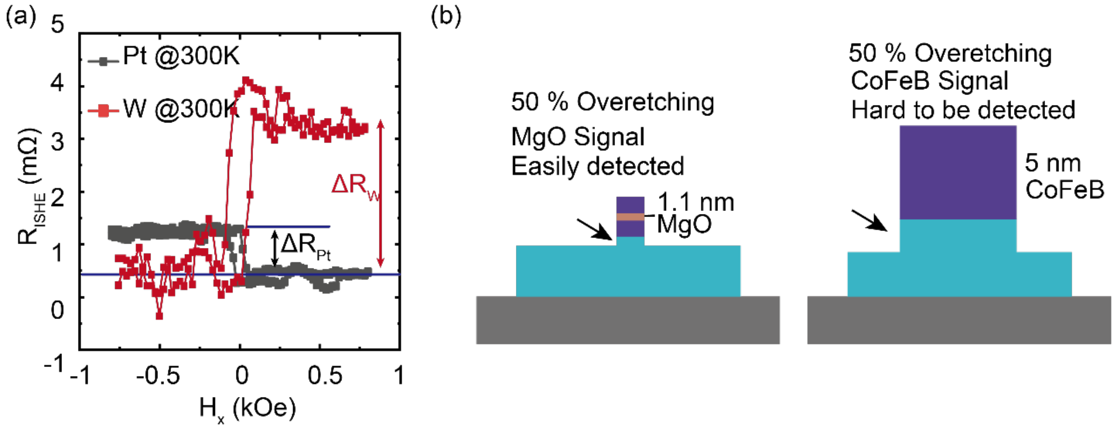


**Figure R9**. (a) Signal of microdot device with different channel material. (b) Over-etching in fabrications.

*Reviewer wrote*: (*Question 5)* Obviously, the top pinned CoFeB will exert the spin

transfer torque of the bottom free layer. Will the supply current switch the magnetization of the bottom CoFeB in the absence of an external field? Is it the reason why the measurements of the current source are only below 40μA?

*Our Response*:

Thank you for your insightful questions. The current density corresponding to the applied current is lower than the critical current density for spin-transfer torque (STT) switching, and the thickness and resistance-area product (RA) characteristics of the MgO barrier layer further weaken the effect of STT.

Firstly, the current density corresponding to the maximum current is lower than the STT critical value. STT-driven magnetization switching requires a sufficient spin-polarized current to pass through the magnetic tunnel junction (MTJ) and transfer torque. However, the current amplitude used in this study is relatively low, and the generated STT is insufficient to overcome the coercive force and magnetic anisotropy energy barrier of the free layer, making it impossible to induce a change in the magnetization direction. For example, the nano-device dimension is around 60 nm × 130 nm, the maximum current of the current source (voltage source) in the experiment is 40 μA (51 μA), and the corresponding current density $5.1\times10^{9}$ $A/m^2$ ($6.5\times10^{9}$ $A/m^2$) is much lower than the critical current density (~$10^{10}$ $A/m^{2)}$ for STT required to switch the magnetization of the bottom CoFeB free layer (**Nat. Mater., 2010, 9, 721**).

Secondly, the relatively thick MgO layer and large RA value weaken the STT effect. The optimal MgO layer thickness in the experiment is 1.1 nm, and the RA value of the MTJ is relatively high at this thickness. According to STT-related theories (**Phys. Rev. B, 2017, 96, 064437**), the larger the RA value, the weaker the effective torque transferred to the free layer. Meanwhile, the thick MgO barrier layer reduces the spin tunneling, further weakening the regulatory effect of STT on the magnetization state of the bottom CoFeB free layer. Therefore, the general thickness of MgO in a STT-MTJ is 0.8 nm.

In conclusion, in the absence of an external magnetic field, the supply current cannot switch the magnetization of the bottom CoFeB free layer. We have added more

sentences in the revised manuscript to clarify the impacting of STT during measurement.

***Corresponding change in manuscript:*** *Yes*

*Location of Change: Part 2.3, Paragraph 1, Page #7*

We have added expression in Part 2.3: 'Note that the corresponding current density ($6.5\times10^9$ A/m$^2$) is far away from the critical value to induce spin-transfer-torque switching of magnetization in our stacks.'

*Reviewer wrote*: (*Question 6)* Correct the typos in the manuscript. For example, On Page 7, line 202, the second term in square brackets did not match the Eq. 1 derived in the Supplementary Material on Page 4, line 77. Similarly, on Page 15, line 358, correct the unit in Figure 4a inset.

*Our Response*:

Thank you for your detailed comments. There are some typographical errors in the main text.

The same parameter in equation (*e.g.* MTJ resistance at AP state) is expressed using different notations ($R_{MTJ}^{AP}$ in Supplementary Material and $R_{MTJ,AP}$ in main text), leading to confusion in reading. We have modified this problem by standardizing all to the format of like $A_{name}^{condition}$, where A is parameter, subscript is name of A, and superscript is current condition. The voltage unit in Figure 4a inset is corrected to mV.

We have carefully polished the language and modified some spelling/grammar mistakes.

***Corresponding change in manuscript:*** *Yes*

*Location of Change: Part 2.3, Paragraph 2 and 3, Page #7 and Figure 4a, Page #16*

$R_{MTJ,AP}$ ($R_{xy,P}$) has been modified to $R_{MTJ}^{AP}$ ($R_{xy}^{P}$), and the voltage unit in Figure 4a inset is corrected to mV.

We have deleted expression 'by the formula, $\Delta R_{xy} = R_{xy,P} - R_{xy,AP} = V_{xy,P}/I_P - V_{xy,AP}/I_{AP}$' in Part 2.3, Paragraph 2, Page #7.

*Location of Change: Part 1, Paragraph 4, Page #3*

We have added expression in Part 1: ‘Thanks to the current modulation and spin filtering by MTJ, as well as high performance W SOC channel’ in Part 1, Paragraph 4, Page #3, to make language and logic clearer.

*Reviewer wrote*: (*Question 7)* The format of the references should keep the same style.

*Our Response*:

Thank you for your detailed comments. We have modified the format of references to keep the same style.

***Corresponding change in manuscript:*** *Yes*

*Location of Change: Part Reference*

We have modified the format of references to keep the same style.

We are very grateful for your constructive comments and acknowledgement on this work. Your suggestions helped us improve the clarity and quality of this work, and lead the direction of future research. We sincerely hope that the point-by-point response will effectively address your concerns and that the revised manuscript will fully meet your requirements. Thank you again for your patience and detailed comments.

**Response Letter**

Dear Editor and esteemed Reviewers,

We would like to express our sincere gratitude to you for the careful review of our revised manuscript entitled "Giant Spin-Orbit Magnetic State Readout Enhanced by a Magnetic Tunnel Junction" (ID: NCOMMS-25-83756B) and for the constructive and insightful comments that have further guided us to polish and improve our work. We greatly appreciate the time and effort you have devoted to evaluating our manuscript, and we are particularly thankful for the positive feedback and valuable suggestions from the reviewers, which not only point out the remaining key issues to be addressed but also help us to make the content of the manuscript more rigorous, clear and comprehensive.

We have carefully studied and fully understood all the remaining comments raised by the reviewers, and made in-depth revisions and detailed explanations for each point in the following revision. We have also highlighted the changes made in the main manuscript in red color so that they can be located easily (changes in blue color is the previous round of revision). We will spare no effort to ensure that the revised manuscript meets the high standards of Nature Communications.

Again, we would like to extend our heartfelt thanks to Editor Yang and all the reviewers for your professional guidance and valuable support. We look forward to your further comments on our revised manuscript.

Yours sincerely,

Prof. Weisheng Zhao, on behalf of the authors

National Key Laboratory of Spintronics, Hangzhou International Innovation Institute, Beihang University, Hangzhou, China

Email: weisheng.zhao@buaa.edu.cn

**Comments to Authors:**

***Reviewer #1***

***Comment #***

*Reviewer wrote*: *(Question 1)* The authors describe the proposed MTJ-enhanced MESO write process as follows: "An input voltage $V_{in}$ charges the capacitor of multiferroic material (ME layer, depicted in dark blue), switching the ferroelectric polarization." However, it is unclear how $V_{in}$ can switch the ferroelectric polarization. The ferroelectric polarization must be able to change depending on $V_{in}$, yet $V_{in}$ is always smaller than $V_{supply}$ and larger than GND, while the top terminal of the ME layer is always connected to $V_{supply}$. Therefore, a clear explanation is needed on how the ferroelectric polarization can change depending on $V_{in}$ (when $V_{in}$ increases, when $V_{in}$ decreases).

Our Response:

We greatly appreciate your detailed comment. We notice that your concerns are concentrated on circuit-level device operations, where accurate descriptions are insufficient in our manuscript. We recall the device and its circuit model below as **Figure RR1**, and explain how the ferroelectric polarization changes depending on $V_{in}$.

First of all, in MESO logic device, there is a spin injection (SI) layer between ferromagnetic (FM) layer and spin-orbit-coupling (SOC) layer (**Figure RR1a**) to optimize interface, match spin conductance and suppress spin backflow (**Nature, 2019, 565 (7737), 35–42; Nature, 2002, 416 (6882), 713–716; Nat. Phys., 2014, 10 (8), 561–566**). SI is usually selected to be an insulator or semiconductor, such as several nanometers of MgO or $Al_2O_3$, which yields large resistance between FM and GND. The writing process is based on ferroelectric (FE) polarization switching of magnetoelectric (ME) layer, and the initial charging voltage is ($V_{in}$ - $V_A$). As FE switching bidirectionally requires bipolar voltage, the remaining question is how can ($V_{in}$ - $V_A$) be bipolar. For cascading, $V_{in}$ is connected by last level $V_{out}$, and the question turns to be how can ($V_{out}$ - $V_A$) be bipolar. Considering heavy metal SOC layer with finite resistivity and there is another tunneling barrier MgO in MTJ structure in our proposed MTJ-enhanced MESO, the voltage of point A (SOC layer, **Figure RR1b**), $V_A$, is just

little larger than GND, but much smaller than $V_{supply}$. ($V_{out}$ - $V_A$) is actually the spin to charge conversion signal, which is inherently bipolar promised by SOC mechanism (**Figure RR1b**). When FM is spin right, for example, the converted charge flowing forward, leading to positive conversion voltage, and When FM is spin left, the converted charge flowing backward, leading to negative conversion voltage.

Now we considering W in experiments as SOC layer. In circuit model of MTJ-enhanced MESO as shown in **Figure RR1c**, vertical resistance of SOC layer $R_{soc,v}$ is given by $\rho\frac{l_v}{w_I l_I}$, where ρ is resistivity (~367 μΩ·cm), $w_I$ (60 nm) and $l_I$ (130 nm) are width and height of current path, respectively, $l_v$ is effective vertical length (several nanometers, $l_v$ is less than W thickness d). $R_{soc,v}$ is calculated to be less than 10 Ω. Given that $R_{MTJ}$ is several kΩ, even without SI layer, $V_A$ is almost short to GND. As comparison, horizontal resistance of SOC layer $R_{soc,h}$ given by $\rho\frac{l_h}{wd}$ is in kΩ, where $w$ and $d$ are width and height of current path, respectively, $l_h$ is effective horizontal length.

In conclusion, the FE polarization of ME layer is switched by ($V_{in}$ - $V_A$), and $V_A$ is almost short to GND, thus FE polarization is determined by $V_{in}$. When $V_{in}$ increases and exceeds the positive threshold voltage, FE polarization can be positive, and when $V_{in}$ decreases and exceeds the negative threshold, FE polarization is switched to be negative. Due to ME coupling, the ferromagnetism changes with FE polarization, and the writing of MESO can be accomplished.

***Corresponding change in manuscript:*** *Yes*

*Location of Change: Part 1, Paragraph 3, Page #2*

To make clear the MTJ-enhance MESO logic operation process, the below expression has been modified: '$V_{in}$ charges the capacitor of multiferroic material (depicted in dark blue), switching in sequence the ferroelectric polarization, interface exchange bias between ME layer and FM layer, and magnetization of FM layer' **has been modified to** '$V_{in}$ charges the capacitor of multiferroic material (depicted in dark blue) and when the voltage on ME layer exceeds its threshold, the ferroelectric

polarization, interface exchange bias between ME layer and FM layer, and magnetization of FM layer switch in sequence.'

*Location of Change: Part 1, Paragraph 3, Page #3*

The below expression has been modified: 'In addition, we also simplified the MTJ-enhanced MESO logic device into an equivalent circuit to analyze the its operation in the cascaded structure (**Supplementary Material S1**).' **has been modified to** 'In addition, we also simplified the MTJ-enhanced MESO logic device into an equivalent circuit to analyze the its operation in the cascaded structure and clarify the writing process in circuit level (**Supplementary Material S1**).'

*Location of Change: Supplementary Material S1*

We have added more analysis and modified some expression in **Supplementary Material S1** to clarify the writing process.

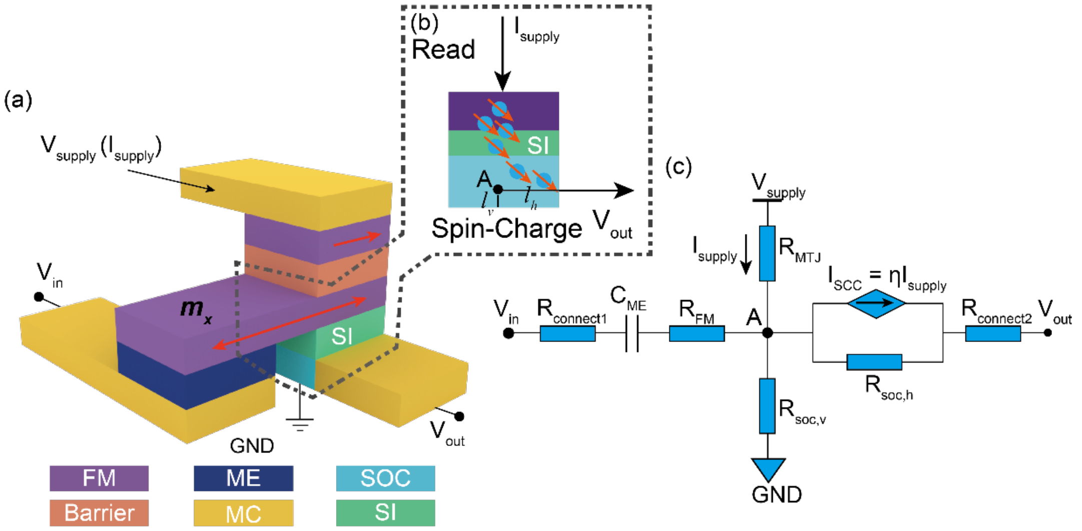


Figure RR1. Schematic of MTJ-enhanced MESO and its equivalent circuit diagram. (a) Schematic of MTJ-enhanced MESO logic device. (b) Reading process. (c) Equivalent circuit diagram MTJ-enhanced MESO logic device.

*Reviewer wrote*: (*Question 2)* In Figure R1, $V_{out}$ appears to be simply connected to GND. Figure R3 includes $R_{soc,v}$, but this resistance is expected to be much smaller than $R_{MTJ}$. In this case, $V_{out}$ is interpreted similarly to GND. Furthermore, Figure R1(d) states that $V_{out}$ can be either a positive or negative voltage depending on $V_{in}$. The authors also mention that $V_{out}$ is $-\Delta V_{xy}/2$ or $\Delta V_{xy}/2$. However, since the MTJ (FM layer, SI layer,

SOC layer) is connected to $V_{supply}$ and GND, $V_{out}$ cannot be a negative voltage. A clear explanation of this point is considered necessary.

*Our Response*:

Thank you for your valuable comment. We are sorry that some descriptions in **Figure R1**, **Figure R3** and **Supplementary Material S1** are confusing. It is true that $V_{out}$ is interpreted similarly to GND, and $V_{out}$ can be either a positive or negative voltage depending on $V_{in}$ (as $V_{in}$ determines free layer magnetization). $V_{out}$ can be negative, which is promised by SOC mechanism.

As we mentioned in *Response* to *Question 1*, the voltage of point A (position of SOC layer, **Figure RR1b**), $V_A$, is almost short to GND. Thus, spin to charge conversion signal ($V_{out}$ - $V_A$) is equal to $V_{out}$. Due to SOC and inverse spin Hall effect, When FM is spin right, for example, the converted charge flowing forward, leading to positive conversion voltage $V_{out}$ ($\Delta V_{xy}/2$), and When FM is spin left, the converted charge flowing backward, leading to negative conversion voltage $V_{out}$ ($-\Delta V_{xy}/2$). $V_{out}$ is a generated electric force by physical mechanism, not a voltage depending on voltage division.

***Corresponding change in manuscript:*** *Yes*

The corresponding modification is same with that in Response to Comment 1.

We are very grateful for your constructive comments from the point of electronic circuit. Most of our researchers major in material and device and your suggestions helped us improve the clarity and quality of this work. We sincerely hope that the point-by-point response will effectively address your concerns and that the revised manuscript will fully meet your requirements. Thank you again for your patience and detailed comments.

***Reviewer #2***

***Comment #***

*Reviewer wrote*: The authors have addressed all my comments point-by-point in their response, and I am satisfied with their revisions. I therefore recommend the manuscript for acceptance by the journal.

*Our Response:*

We greatly appreciate your acknowledgement of this work and your previous constructive comments. We are happy that our supplemented work and response addressed your concerns. Your suggestions helped us improve the clarity and quality of this work, and lead the direction of future research. Thank you again for your patience and recommendation.

***Reviewer #3***

***Comment #***

*Reviewer wrote*: I would like to thank the authors for their detailed responses. I am pleased to see that the concerns regarding the readout mechanism have been well-addressed. The integration of a Magnetic Tunnel Junction (MTJ) with the spin-orbit (SO) component is indeed a brilliant idea, and the rebuttal clearly demonstrates how this enhances the readout voltage effectively.

*Our Response:*

We greatly appreciate your positive assessment of this work, and your previous constructive suggestions which helped us improve the clarity and quality of this manuscript. We are happy that our supplemented work and response addressed your concerns.

*Reviewer wrote*: However, I still have a significant reservation regarding the magnetoelectric (ME) component of the proposed system. While the readout module is now well-defined, the authors have not sufficiently clarified what specific ME material system is claimed to support this MTJ-based MESO logic.

Specifically, do the authors intend to stick with $BiFeO_3$ (BFO) as the multiferroic element? If BFO is indeed the material of choice, I must point out that the Pulsed Laser Deposition (PLD) process typically required for high-quality BFO is unfriendly to standard CMOS processes. This raises a critical question about the practical scalability of the device: How do the authors envision making such a MESO structure commercially available and compatible with CMOS-acceptable processes?

While the enhanced readout voltage is a major step forward, the feasibility of the ME writing stage is equally critical for the device's viability. I would appreciate a more detailed discussion on the material selection for the ME layer and a proposed roadmap for its integration using industry-standard fabrication techniques.

*Our Response*:

Thank you for your insightful questions. Writing process of ME block is very critical for applications. The mechanism of writing is based on voltage-controlled

ferromagnetic order. To achieve this, the ME material is the key point.

Until now, room temperature single-phase ME materials are rare. According to experimental reports, $BiFeO_3$ (BFO) and $Cr_2O_3$ can be candidates. The Néel temperature of $Cr_2O_3$ is around 300 K (**Nat. Mater., 2010, 9 (7), 579–585; Nat. Mater., 2018, 17 (7), 577–580**), too low for daily application, but could be employed in specific situation, such as aerospace electronic device working at low temperature. Moreover, $Cr_2O_3$ film can be deposited using magnetron sputtering (**Appl. Phys. Lett., 2014, 104 (15), 152409; Appl. Phys. Lett., 2015, 106 (13); Nat Commun., 2022, 13 (1), 6745**).

BFO possesses high ferroelectric Curie temperature (~1100 K) and antiferromagnetic Néel temperature (~640 K) (**Nat. Mater., 2006, 5 (10), 823–829**). However, previous deposition of BFO film highly relies on Pulsed Laser Deposition (PLD) process (**Nat Commun., 2024, 15 (1), 1902**; **Nat Commun., 2020, 11 (1), 2836.**), unfriendly to standard CMOS processes. Recently, using magnetron sputtering, our collaborators have successfully grown La-Doped BFO (LBFO) film, and demonstrated large exchange bias in LBFO/CoFeB heterostructure at room temperature (**Adv. Phys. Res., 2025, e00113, doi: 10.1002/apxr.202500113**). This is a great progress for BFO applications, and puts forward a large step of MESO logic.

In addition, the surface roughness of 50 nm BFO is around 0.6 nm, which meets demands of MTJ stacks deposition with in-plane anisotropy (**Adv. Phys. Res., 2025, e00113, doi: 10.1002/apxr.202500113**). Thus, in the coming studies, the LBFO-based ME writing module and MTJ-enhanced readout module can be integrated together via micro-nano processing technology to construct MTJ-enhanced MESO logic as schematically shown in Figure 1(a) in the main text.

In conclusion, the most suitable ME material for daily usage is still BFO until now, and CMOS-acceptable deposition of BFO has been partially solved. Your valuable suggestions really help to point the direction of future research.

***Corresponding change in manuscript:*** *Yes*

*Location of Change: Part 3, Paragraph 4, Page#10*

We have added expression below in *Part 3.*

‘In addition, CMOS-compatible fabrication of our MTJ-enhanced MESO device is a key point before its practical application. Recently, La-doped $BiFeO_3$ (LBFO) ME films have been successfully grown using magnetron sputtering[53]. Large exchange bias in LBFO/CoFeB heterostructure at room temperature and excellent surface roughness of LBFO have been demonstrated. Thus, in the future studies, the LBFO-based ME writing module and MTJ-enhanced readout module can be integrated together via micro-nano processing technology to construct a MTJ-enhanced MESO logic.’ (Page#10)

Also, A new reference 53 is added.

We are very grateful for your constructive comments and acknowledgement on this work. Your suggestions helped to make the readout mechanism clear and friendly to audience, and lead the direction of future research. We sincerely hope that our response will effectively address your concerns on CMOS-compatible integration. Thank you again for your patience and detailed comments.

**Response Letter**

Dear Editor and Distinguished Reviewers,

We sincerely appreciate your thorough review of our revised manuscript, "Giant Spin-Orbit Magnetic State Readout Enhanced by a Magnetic Tunnel Junction" (ID: NCOMMS-25-83756C), as well as the constructive and insightful comments that have greatly helped us refine and strengthen our work. We are truly grateful for the time and expertise you have dedicated to assessing our manuscript, and we particularly value the positive feedback and thoughtful suggestions, which have identified key areas for improvement and enabled us to enhance the rigor, clarity, and completeness of our presentation.

We have carefully examined and fully addressed all remaining reviewer comments, implementing detailed revisions and providing point-by-point responses in the following sections. Modifications made in this round are highlighted in red for easy identification (revisions from the previous round are marked in blue). We have strived to ensure the revised manuscript aligns with the high academic standards of Nature Communications.

Once again, we extend our sincere thanks to Editor Yang and all reviewers for your professional guidance and invaluable support. We look forward to your further feedback on the updated version of our manuscript.

Yours sincerely,

Prof. Weisheng Zhao, on behalf of the authors

National Key Laboratory of Spintronics, Hangzhou International Innovation Institute, Beihang University, Hangzhou, China

Email: weisheng.zhao@buaa.edu.cn

**Comments to Authors:**

***Reviewer #1***

***Comment #***

*Reviewer wrote*: *(Question 1)* Thank you for providing such a sincere and detailed response to my challenging question from a circuit operation perspective. Most of my doubts have been resolved through your answers. However, I disagree with one part of the authors' response, so I am asking again. The authors answered as follows:

"$V_{out}$ can be negative, which is promised by the SOC mechanism."

"$V_{out}$ is an electric force generated by a physical mechanism, not a voltage dependent on voltage division."

However, the FM-SI-SOC is connected to $V_{supply}$ and GND. Considering the operation within the SOC layer, electrons flow from GND to $V_{supply}$ (opposite to the current flow). Yet, electrons aligned with a specific spin due to the spin-Hall effect, centered on node A, can be concentrated and flow toward one side of the SOC layer. Consequently, I believe the $V_{out}$ can increase or decrease relative to the A node voltage depending on the FM spin direction. However, from this operational perspective, I do not think the SOC mechanism can generate a voltage higher than $V_{supply}$ or lower than GND. This is because current flows through the SOC layer in a voltage division configuration.

As the author's response suggests, if the SOC mechanism can generate a voltage lower than GND (similar to a charge pump in a circuit), further explanation would be necessary.

Our Response:

We greatly appreciate your rigorous comment on operational perspective. You are right and we agree that $V_{out}$ can not be lower than GND because current flows through the SOC layer which is good conductor and connected to GND, although $V_{out}$ can increase or decrease relative to the A node voltage depending on the FM spin direction. We are sorry we explained this in a debatable way before. According to your suggestion, we analyze the operation mode of MTJ-enhanced MESO carefully. We find that "$V_{in}$ - $V_A$" can be positive or negative, acting on the magnetoelectric (ME) capacitor to drive writing operation. The detailed analysis unfolds as follows:

We recall the equivalent circuit diagram of our MTJ-enhanced MESO below as **Figure R1(a).** The MESO writing process is based on ferroelectric (FE) polarization switching of ME layer, and the initial charging voltage is ($V_{in}$ - $V_A$). For cascading, $V_{in}$ is connected by last level $V_{out}$, *i.e.* $V_{in}$ = $V_{out}$, and the charging voltage is ($V_{out}$ - $V_A$). We can find that ($V_{out}$ - $V_A$) is actually the spin to charge conversion signal due to SOC mechanism. When FM is spin right, for example, the converted charge flows forward, leading to positive cascading voltage ($V_{out}$ - $V_A$), *i.e.* $+\Delta V_{xy}/2$, and When FM is spin left, the converted charge flows backward, leading to negative cascading voltage ($V_{out}$ - $V_A$), *i.e.* $-\Delta V_{xy}/2$. The absolute value for cascading is always $\Delta V_{xy}/2$, let alone $V_{out}$ is higher or lower than GND. This voltage is greatly influenced by spin Hall angle ($\theta_{SH}$) of SOC layer, spin polarization rate (SP) of FM, and current modulation by MTJ, as discussed in the main text.

We then conduct finite element simulations to directly show voltage distribution of device and output cascading voltage ($V_{out}$ - $V_A$) as function of external field. In experiment, $V_{xy}$ is defined as voltage difference between node 2 and node 4, as shown in **Figure R1(b)**, and in the concept of MTJ-enhanced MESO, $V_{out}$ denote the voltage of node 2 (represented by $V_{out}(2)$ in simulations). We use a single FM layer with SP = 0.9, and SOC channel with $\theta_{SH}$ = -0.3, corresponding to the effective parameters of MTJ-enhanced MESO in the main text. Results are shown in **Figure R1(c)~(f)**. **Figure R1(c)** presents the body voltage distribution under 100 Oe magnetic field and 100 μA current supply. There is obvious color difference between measurement node 2 and node 4, indicating SOC-induced ISHE voltage. **Figure R1(d)** and **Figure R1(e)** are corresponding cross section voltage distribuations in SOC layer of *xy* plane and *xz* plane, respectively, and black arrows denote electric field distribution. **Figure R1(f)** shows normalized voltage of node 2 ($V_{out}(2)$), node 4 ($V_{out}(4)$), node A ($V_A$), anomalous Hall voltage ($V_{AHE}$) generated in ferromagnet and total measured transverse voltage $V_{xy}$ ($V_{xy}$ = $V_{out}(4)$ - $V_{out}(2)$). Both the black curve of $V_{xy}$ and red curve of ($V_{out}(2)$ - $V_A$) spread over positive and negative voltage region. Output voltage $V_{out}(2)$ (blue curve) and $V_{out}(4)$ (green curve) shows opposite polarity, corresponding to SOC machanism. $V_A$ is always constant, larger than GND but lower than $V_{supply}$. $V_{AHE}$ is far smaller than $V_{xy}$.

In conclusion, the converted charge can flow toward node A or output node, leading to positive or nagetive cascading voltage ($V_{out}$ - $V_A$), which can drive the writing operation of MTJ-enhanced MESO.

***Corresponding change in manuscript:*** *Yes*

*Location of Change: Supplementary Material S1*

The below expression has been modified: ‘Considering several-nanometers-thick SOC layer with finite resistivity, $R_{SOC,v}$ is very small, and the voltage $V_A$ of point A (position in SOC layer) is almost short to GND. Thus, in cascaded devices, the voltage ($V_{in}$ - $V_A$) applied to ME, is equal to ($V_{out}$ - $V_A$), and approximately equal to $V_{out}$. Due to SOC and inverse spin Hall effect, When FM is spin right, for example, the converted charge flowing forward, leading to positive conversion voltage $V_{out}$ ($+\Delta V_{xy}/2$), and When FM is spin left, the converted charge flowing backward, leading to negative conversion voltage $V_{out}$ ($-\Delta V_{xy}/2$). Therefore, for an ideal device with no asymmetry, inhomogeneities and parasitic effects, the voltage between $V_{out}$ and GND is $-\Delta V_{xy}/2$ or $\Delta V_{xy}/2$.’ **has been modified to** ‘Due to SOC and inverse spin Hall effect, when FM is spin right, for example, the converted charge flows forward, leading to positive cascading voltage ($V_{out}$ - $V_A$), *i.e.* $+\Delta V_{xy}/2$, and When FM is spin left, the converted charge flows backward, leading to negative cascading voltage ($V_{out}$ - $V_A$), *i.e.* $-\Delta V_{xy}/2$. Therefore, for an ideal device with no asymmetry, inhomogeneities and parasitic effects, the voltage between $V_{out}$ and $V_A$ is $-\Delta V_{xy}/2$ or $\Delta V_{xy}/2$.’

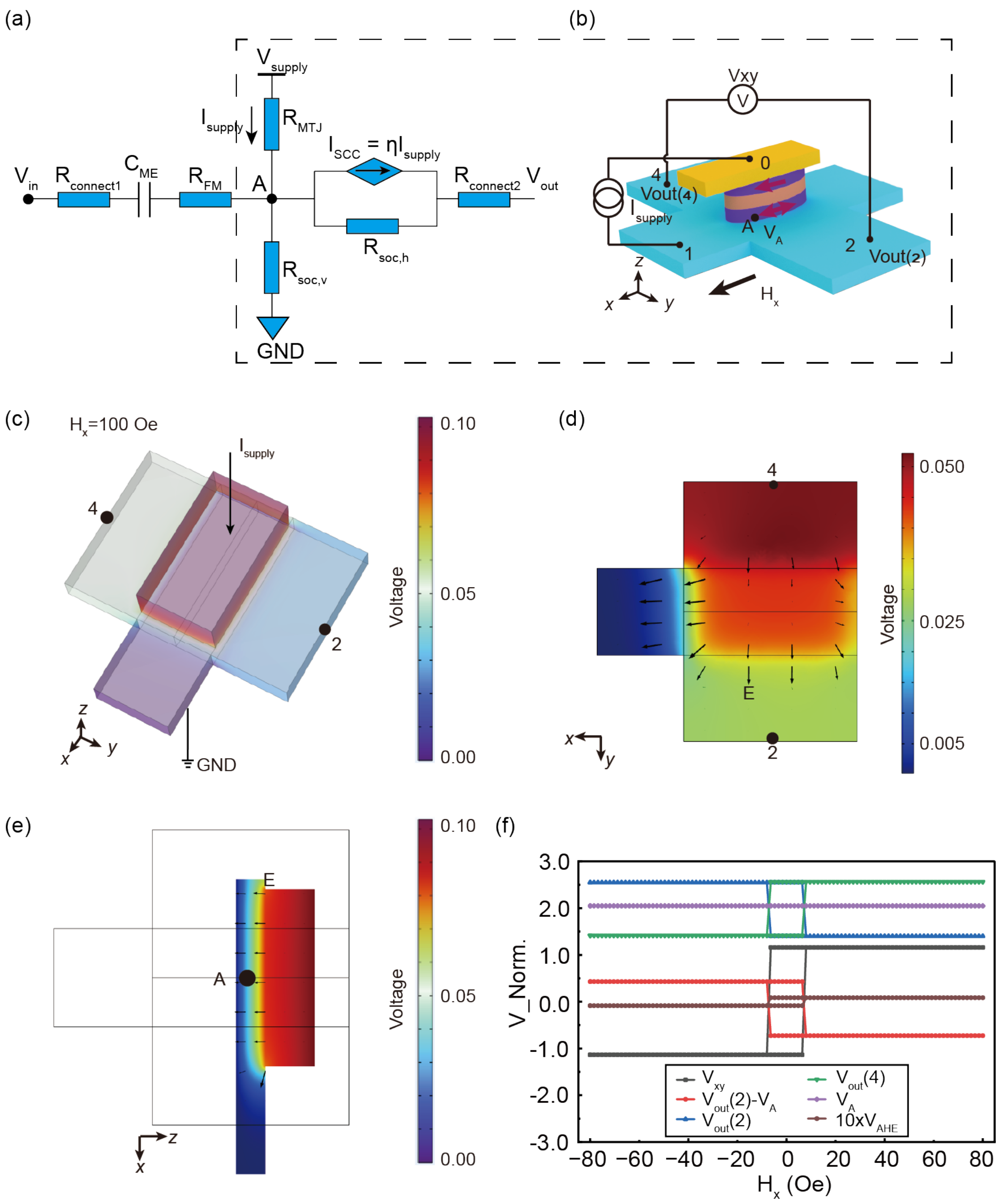


Figure R1. (a) Equivalent circuit diagram MTJ-enhanced MESO logic device. (b) Measurement set-up reflecting to the equivalent circuit. (c)~(f) Finite element simulation results of spin-orbit readout device: (c) body voltage distribution, (d) *xy*-plane voltage distribution, (e) *xz*-plane voltage distribution, and (f) normalized voltage versus magnetic field.

***Reviewer #2 No further comment #***

***Reviewer #3***

***Comment #***

*Reviewer wrote*: The authors have satisfactorily addressed the concerns raised in my previous review. The revisions have improved the clarity and rigor of the manuscript, and I find the current version to be strong and suitable for publication. I therefore recommend acceptance in Nature Communications.

*Our Response:*

We greatly appreciate your acknowledgement of this work and your previous constructive comments. We are happy that our supplemented work and response addressed your concerns. Your suggestions helped us improve the clarity and quality of this work, and lead the direction of future research. Thank you again for your patience and recommendation.

**Response Letter**

**Comments to Authors:**

***Reviewer #1***

***Comment #***

*Reviewer wrote*: Thank you. I have no further questions regarding this manuscript.

Our Response:

We greatly appreciate your rigorous comment on operational perspective. Your suggestions helped us improve the clarity and quality of this work, and lead the direction of future research. Thank you again for your patience.
